# The HIF-1α antisense long non-coding RNA drives a positive feedback loop of HIF-1α mediated transactivation and glycolysis

**DOI:** 10.1038/s41467-021-21535-3

**Published:** 2021-02-26

**Authors:** Fang Zheng, Jianing Chen, Xiaoqian Zhang, Zifeng Wang, Jiewen Chen, Xiaorong Lin, Hongyan Huang, Wenkui Fu, Jing Liang, Wei Wu, Bo Li, Herui Yao, Hai Hu, Erwei Song

**Affiliations:** 1 Guangdong Provincial Key Laboratory of Malignant Tumor Epigenetics and Gene Regulation, Sun Yat-Sen Memorial Hospital, Sun Yat-Sen University, Guangzhou, 510120 China; 2 Medical Research Center, Sun Yat-Sen Memorial Hospital, Sun Yat-Sen University, Guangzhou, 510120 China; 3 Breast Tumor Center, Sun Yat-Sen Memorial Hospital, Sun Yat-Sen University, Guangzhou, 510120 China; 4 State Key Laboratory of Oncology in South China, Sun Yat-Sen University Cancer Center, Collaborative Innovation Center for Cancer Medicine, Guangzhou, 510060 China; 5 Chongqing Key Laboratory of Molecular Oncology and Epigenetics, the First Affiliated Hospital of Chongqing Medical University, Chongqing, 400016 China; 6 Department of Oncology, Sun Yat-Sen Memorial Hospital, Sun Yat-Sen University, Guangzhou, 510120 China; 7 Department of Biochemistry and Biophysics, School of Basic Medical Sciences, Peking University Health Science Center, 38 Xueyuan Road, Beijing, 100191 China; 8 Bioland Laboratory, Guangzhou, 510005 China; 9 Fountain-Valley Institute for Life Sciences, 4th Floor, Building D, Guangzhou Institute of Biomedicine and Health, Chinese Academy of Sciences, 190 Kaiyuan Avenue, Huangpu District, Guangzhou, 510535 China

**Keywords:** Cancer metabolism, Cell biology, Long non-coding RNAs

## Abstract

Hypoxia-inducible factor-1 (HIF-1) is a master driver of glucose metabolism in cancer cells. Here, we demonstrate that a *HIF-1α* anti-sense lncRNA, HIFAL, is essential for maintaining and enhancing HIF-1α-mediated transactivation and glycolysis. Mechanistically, HIFAL recruits prolyl hydroxylase 3 (PHD3) to pyruvate kinase 2 (PKM2) to induce its prolyl hydroxylation and introduces the PKM2/PHD3 complex into the nucleus via binding with heterogeneous nuclear ribonucleoprotein F (hnRNPF) to enhance HIF-1α transactivation. Reciprocally, HIF-1α induces HIFAL transcription, which forms a positive feed-forward loop to maintain the transactivation activity of HIF-1α. Clinically, high HIFAL expression is associated with aggressive breast cancer phenotype and poor patient outcome. Furthermore, HIFAL overexpression promotes tumor growth in vivo, while targeting both HIFAL and HIF-1α significantly reduces their effect on cancer growth. Overall, our results indicate a critical regulatory role of HIFAL in HIF-1α-driven transactivation and glycolysis, identifying HIFAL as a therapeutic target for cancer treatment.

## Abstract (web summary)

HIF1alpha is reported to drive tumourigenesis through activating glycolysis. Here, the authors show that HIFAL, the HIF1alpha antisense long non-coding RNA, and HIF1alpha form a positive feed-forward loop which is essential for HIF1a-mediated metabolic reprogramming and oncogenic role.

## Introduction

Hypoxia is one of the major feature of the tumor microenvironment, which induces massive production of angiogenic factors, chemokines and bioactive mediators to promote tumor progression and metastasis^1,2^. The competence of tumor cells to endure oxygen depletion is largely due to accumulation of Hypoxia-inducible factor 1 (HIF-1), a transcription factor consisting of an O_2_-responsive HIF-1α subunit and a constitutively expressed HIF-1β subunit^3^. The activation of HIF-1α contributes to the Warburg effect through a switch from oxidative phosphorylation to glycolysis. Upon hypoxia, HIF-1 binds to the hypoxia response elements (HREs) of target genes to drive their transcription^4^. HIF-1 target genes, including the genes coding glycolytic receptors and enzymes, including glucose transporter GLUT1, hexokinase II (HKII), lactate dehydrogenase A (LDHA), and pyruvate dehydrogenase kinase 1 (PDK1)^5–8^, switch the tumor cells from oxidative to anaerobic glycolysis in order to adapt to tumor hypoxic condition^5,9^. Therefore, glycolysis is an important target of HIF-1 and can serve as a marker of HIF-1 mediated transactivation. Targeting HIF-1 emerges as an ideal strategy to suppress glycolysis for cancer treatment. Although numerous inhibitors are under development to selectively intervene the HIF-1 pathway^10^, including an LNA-based anti-sense oligonucleotide (EZN-2968) that inhibits HIF-1α mRNA and demonstrates limited anti-tumor effect in phase I trial^11,12^, targeting HIF-1α per se seems not to be effective in reversing glucose metabolic reprograming and appears toxic^7^. In addition, inhibiters of HIF-2 that disrupting HIF/ARNT dimer formation have also been developed for cancer treatment^13,14^. In this context, it is tempting to suggest that inhibiting HIF-1 mediated transactivation, rather than directly targeting the transcriptional factor itself, could be more promising for cancer treatment strategy, but such approaches are still lacking. Therefore, there is a pressing need to elucidate the regulatory mechanisms of HIF-1 transactivation to develop effective strategies against hypoxia-mediated tumor progression.

It has been shown that pyruvate kinase isozymes M2 (PKM2) acts as the essential co-activator to stimulate HIF-1 transactivation in tumor cells^6,15^. Particularly under hypoxic condition, PHD3 binds to PKM2 to induce its prolyl hydroxylation in the cytoplasm. Then, the PKM2/PHD3 complex is transported into the nucleus and assists recruitment of HIF-1 as well as p300 to form a transcriptional complex at the hypoxia response elements (HRE)^6^. Although PKM2 phosphorylation at S37 by ERK1^16^ and hydroxylation at P403 and P408 by PHD3 is associated with its nuclear translocation^6^, the underlying mechanism of inducing the nuclear transportation of PKM2/PHD3 complex remain largely unknown.

Long noncoding RNAs (lncRNAs) are a class of non-protein-coding RNA transcripts that are longer than 200nt and are involved in numerous physiological and pathological processes through epigenetic regulation and related signal transduction^17–20^. Recently, accumulating evidence has suggested that lncRNAs may act as key regulators in cellular signal transduction pathways by interacting with major signaling proteins. For instance, our previous study showed that NKILA lncRNA suppresses NF-κB activation by interacting with the NF-κB/I-κB complex, and thus inhibits cancer metastasis^21^. Moreover, we and others have recently shown that lncRNAs participate in the metabolic reprograming of glucose in cancer cells. For example, linc-p21^22^ and HISLA^23^ stabilize HIF-1α protein by blocking its interaction with VHL and PHD2 respectively, and thus enhance glycolysis in tumor cells. Herein, we investigated whether lncRNAs may regulate HIF-1-driven transactivation under hypoxic conditions, and whether lncRNAs may serve as therapeutic targets to inhibit the glycolysis of tumor cells and HIF-1-mediated cancer progression. Our study revealed a lncRNA HIFAL played a critical regulatory role in HIF-1α-driven transactivation and glycolysis, supporting HIFAL as a therapeutic target for cancer treatment.

## Results

### HIFAL is essential for maintaining HIF-1 transactivation

**Fig. 1:**
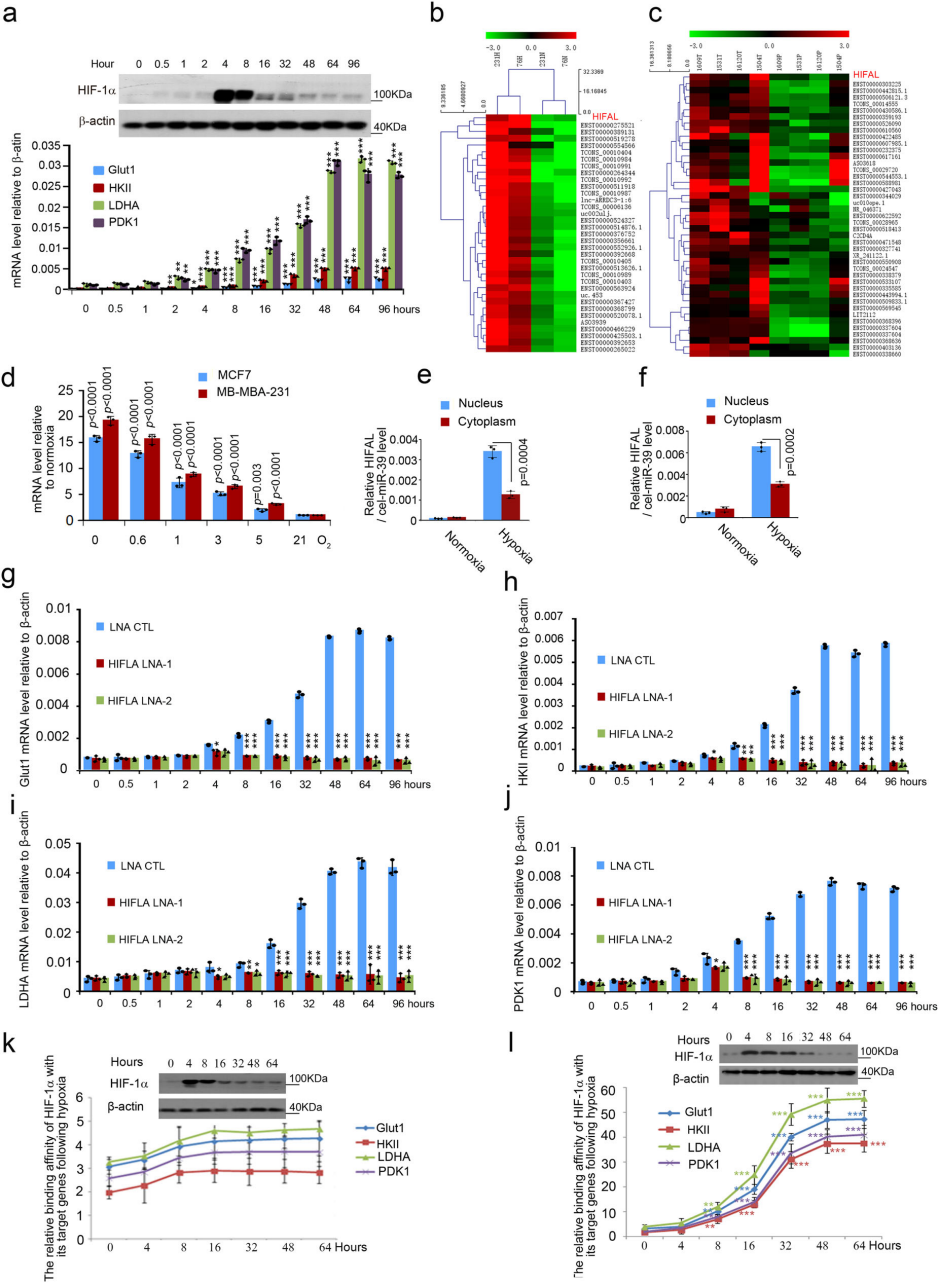
LncRNA HIFAL is essential for maintaining HIF-1α triggered transcription under hypoxia. **a** The kinetics of HIF-1α protein level under hypoxia, determined by immunoblotting, was not parallel with that of its target gene transcription by qRT-PCR in MDA-MB-231 cells. *p* = 0.0055, *p* = 0.0062, *p* = 0.0038 at 2 h of HKII, LDHA, PDK1; *p* = 0.0219 at 4 h of GLUT1; *p* < 0.0001 at 8h-96h of GLUT1, HKII, LDHA, PDK1. **b** Hierarchical clustering for the lncRNAs differentially expressed in MDA-MB-231 and 76 N cells, cultured under normoxia (20%) or hypoxia (0.6%) conditions (fold change > 10, *p* < 0.05, two-sided paired t-test). **c** Microarray analysis of lncRNA expression in 4 pairs of breast cancer and adjacent normal tissues. (*p* < 0.05, two-sided paired t-test). **d** HIFAL level increases upon hypoxia, as measured by qRT-PCR. **e**, **f** HIFAL translocates into the nucleus upon hypoxia. The relative level of HIFAL in cytoplasmic and nuclear extract of MCF-7 (**e**) and MDA-MB-231 (**f**) cells were detected by qRT-PCR. Exogenous cel-miR-39 was added into the cytoplasmic or nuclear-fractionated RNA as an independent control. **g**–**j** After knocking down HIFAL, the kinetics of HIF-1α protein level under hypoxia was parallel with that of its target gene transcription. QRT-PCR was used to examine the mRNA expression of indicated genes GLUT1 (**g**), HKII (**h**), LDHA (**i**), PDK1 (**j**) in MDA-MB-231 cells (CTL vs LNA1 *p* = 0.015, *p* = 0.03, *p* = 0.04, *p* = 0.038, respectively, at 4 h in **g**, **h**, **i**, **j**. CTLvsLNA2 p = 0.02 at 4 h in **g**. CTLvsLNA1, *p* < 0.0001, *p* = 0.0012, *p* = 0.0062, *p* < 0.0001; CTLvs LNA2, *p* < 0.0001, *p* = 0.0011, *p* = 0.0159, *p* = 0.0002 at 8 h in **g**, **h**, **i**, **j**. CTLvsLNA1, CTLvsLNA2, *p* < 0.0001at 16h-96h in **g**, **h**, **i**, **j**. The HIFAL levels in cells are shown in the upper panel of (**g**). **k**, **l** Binding kinetics of HIF-1α to its target genes keeps increasing after hypoxia, which is dependent on HIFAL. The HIF-1α ChIP experiments were performed in the HIFAL KO (**k**) or WT MDA-MB-231 cells (**l**) at indicated time points after hypoxia and normalized by the corresponding HIF-1a protein levels. *P* = 0.0027, *p* = 0.0092, *p* = 0.0025, *p* = 0.0032 at 8 h, *p* < 0.0001 at 16h-64h of GLUT1, HKII, LDHA, PDK1. For **a**, **d**–**l**, *p* values were determined by two-sided unpaired t-test. Graphs show means ± SD of experimental triplicates. ***p < 0.001, **p < 0.01, *p < 0.05. Source data are provided as a Source Data file.

To investigate the contributions of HIF-1α in regulating glycolysis of cancer cells under hypoxia, we evaluated the dynamics of HIF-1α protein and its target genes related to glucose metabolism, including GLUT1, HKII, LDHA, and PDK1^5^, following prolonged hypoxia. HIF-1α protein peaked at 4 h after the MDA-MB-231and MCF-7 breast cancer cells were placed under hypoxic conditions, and gradually reduced to background level at 48 h (Fig. 1a, Supplementary Fig. 1a), which was in agreement with findings from other groups^24–26^. Additionally, the chromatin-associated HIF-1α decreased along with the prolonged hypoxia (Supplementary Fig. 1b). In line with down-regulation of the HIF-1α protein, HIF-1α mRNA level decreased after hypoxia (Supplementary Fig. 1c). Surprisingly, in contrast to HIF-1α reduction, the mRNA levels of HIF-1α target genes, including GLUT1, HKII, LDHA, and PDK1, kept increasing for 48 h and plateaued up to 30 folds of the basal levels following hypoxia (Fig. 1a).

To explain the discrepancy of dynamic changes in HIF-1α protein and its target gene expression under hypoxia, we hypothesized that a relatively stable co-activator complex was formed at the promoters of HIF-1α target genes to maintain the transactivation activities of the transcription factor even though HIF-1α protein was reduced. Since lncRNAs play an important role in the formation of the protein complex, we compared the lncRNA expression profiles in breast cancer cells under normoxic or hypoxic conditions. A panel of lncRNAs was found to be overexpressed in the hypoxic breast cancer cells (Fig. 1b and Supplementary Fig. 1d). To further determine which of these hypoxia-related lncRNAs are involved in breast cancer development, we searched for the lncRNAs that were also increased in the cancerous mammary tissues as compared with the normal ones (Fig. 1c). An antisense lncRNA of *HIF-1α* (ENST00000554254.1), which we named HIFAL (**HIF A**ntisense **L**ncRNA), was most prominently upregulated in the hypoxic cells and in breast cancer tissues (Fig. 1c and Supplementary Fig. 1d). In addition to HIFAL, two other HIF antisense lncRNAs were identified (Supplementary Fig. 1e), including a natural antisense of HIF-1α transcript (Supplementary Fig. 1e, HIF-AS2) that is involved in negatively regulating HIF-1α expression^27,28^ and another HIF-1α antisense transcript (Supplementary Fig. 1e, HIF-AS1) with unknown functions^29^. More interestingly, the lower the oxygen levels in cell culture condition, the higher the HIFAL expression was detected (Fig. 1d and Supplementary Fig. 1f–h). We amplified HIFAL by 5′ and 3′ RACE, and identified it as a lncRNA of 659 nucleotides (Supplementary Table 1). In addition, HIFAL was enriched in the nuclei upon culturing under hypoxia (Fig. 1e, f and Supplementary Fig. 1i–n). These observations suggested that HIFAL plays a role in regulating the hypoxia response of tumor cells.

To further evaluate whether HIFAL influences HIF-1α transcription, we silenced HIFAL expression using the locked nucleic acid-based antisense oligonucleotides (LNAs) (Supplementary Fig. 1o). Interestingly, at the first 4 h following hypoxic treatment when HIF-1α level increased and peaked, silencing HIFAL does not affect basal levels of the HIF-1α target gene expression. However, after 4 h of hypoxia, the mRNA level of these target genes could not increase and be maintained in HIFAL knockdown cells (Fig. 1g–j). The expression of the hypoxia-inducible genes can also be driven by HIF-2α, which is more stable than HIF-1α under hypoxia. However, a previous study had revealed that HIF-1α but not HIF-2α stimulates glycolytic gene expression^30^. To examine this, the HIF-2α inhibition by RNAi or inhibitor (CAS 882268-69-1) was used. We found that HIF-2α inhibition could not prevent the increase of the HIF-1α targeted glycolic genes expression in prolonged hypoxia (Supplementary Fig. 1p–r). Together, these data suggest that under hypoxic conditions, HIFAL was essential for maintaining high transcription of HIF-1α target genes even after the initial HIF-1α elevation has dropped. To further determine whether HIFAL regulates HIF-1α binding to its target genes, we performed ChIP assay for HIF-1α at various time points following hypoxia and adjusted the results to HIF-1α protein levels. Knocking out HIFAL in MDA-MB-231 cells by deleting its promoter with Cas9 dramatically reduced the amplitude and the duration of HIF-1α binding to its target genes upon hypoxia (Fig. 1k), which could be rescued in the HIFAL wildtype (WT) MDA-MB-231 cells (Fig. 1l). More importantly, the increased binding capacity of HIF-1α with its target genes, rather than HIF-1α expression per se, was associated with the elevation of HIF-1α transcriptional activities (Fig. 1a). On the other hand, mRNA decaying of HIF-1 target genes was not affected by HIFAL knockout in MDA-MB-231 cells following hypoxic treatment (Supplementary Fig. 1s). These results suggested that HIFAL is essential for the binding of HIF-1α protein with its target genes and the related transcriptional activities.

### HIFAL induces propyl hydroxylation of PKM2 through recruiting PHD3

**Fig. 2:**
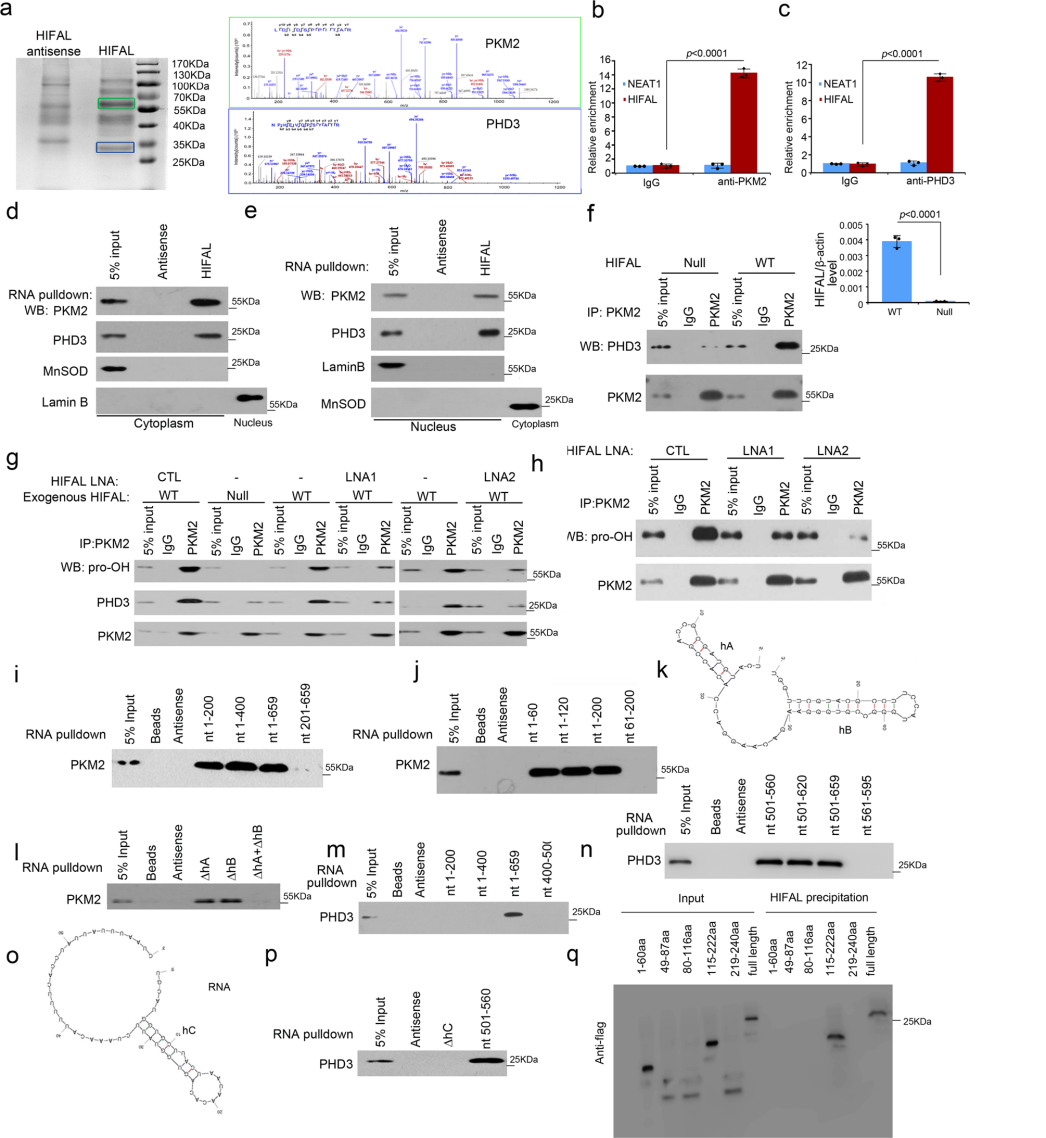
HIFAL promotes the binding of PKM2 to PHD3 and enhances hydroxylation of PKM2 by PHD3. **a** PKM2 and PHD3 were identified as HIFAL binding proteins under hypoxia. The bands in frames, which were specifically precipitated by HIFAL, were submitted for mass spectrometry. The antisense of HIFAL was used as the negative control. **b**, **c** RNA immunoprecipitation assay shows the binding of PKM2 or PHD3 with HIFAL under hypoxia. QRT-PCR was used to detect the RNA level of HIFAL or Neat1in the precipitates. **d**, **e** HIFAL binds to PKM2 and PHD3 in both cytoplasm and nucleus. **f** Enforced HIFAL expression increases the binding of PKM2 with PHD3. HIFAL plasmid was transiently transfected into HIFAL null MDA-MB-231 cells (right panel shows the relative HIFAL level) for 48 h, and then cultured under hypoxia. **g**, **h** HIFAL-LNA reduces the hydroxylation of PKM2 and the binding of PKM2 with PHD3. The HIFAL null MDA-MB-231 cells were transfected with WT HIFAL and treated with HIFAL-LNAs and then cultured under hypoxia (**g**). Knockdown of endogenous HIFAL reduces PKM2 hydroxylation (**h**). **i**–**k** RNA pulldown using sequentially deleted HIFAL fragments shows the binding region of HIFAL with PKM2. nt 1–200 (**i**) and 1–60 (**j**) fragments contain the binding region of HIFAL with PKM2. (**k**) The structure of HIFAL fragment (nt 1–60) predicted by Mfold and RNAfold. **l** Double deletion of hA and hB (ΔhA + ΔhB) abolishes the binding of HIFAL fragment (nt 1–60) with PKM2. **m**–**o** RNA pulldown using sequentially deleted HIFAL fragments shows the binding region of HIFAL with PHD3. nt 501–659 (**m**) and 501–560 (**n**) fragments contain the binding region of HIFAL with PHD3. (**o**) The structure of HIFAL fragment (nt 501–560) predicted by Mfold and RNAfold. **p** Deletion of hairpin c (ΔhC) abolishes the binding of HIFAL fragment (nt 501–560) with PHD3. **q** The truncated PHD3 mutant harboring 115–222aa retains the binding ability with HIFAL. Full length or the PHD3 fragments was transfected into HEK293 cells for HIFAL RNA-pull down assay. For **b**, **c**, **f,** bar graphs represent means ± SD of experimental triplicates, and *p* values were determined by two-sided unpaired t-test. Source data are provided as a Source Data file.

Many of the lncRNAs exert their molecular functions by interacting with proteins^17,31^. To screen for HIFAL interacting proteins, we employed RNA pull-down assays followed by mass spectrometry analysis (Fig. 2a). Among all the proteins that were pulled down by HIFAL, PKM2, and PHD3 aroused our interest since they are major components in the HIF-1α transcriptional complex^6^. We confirmed the interaction of HIFAL with PKM2 and PHD3, respectively, by using RNA pull-down assay, followed by western blotting (Supplementary Fig. 2a) and RNA immunoprecipitation with the antibodies against PKM2 or PHD3 in the MDA-MB-231 cells cultured under hypoxia (Fig. 2b, c). Notably, HIFAL was enriched by around 10 folds in the precipitates with PKM2 or PHD3 antibodies (Fig. 2b,c). Furthermore, invitro binding of the recombinant PKM2 and PDH3 proteins with the purified biotin-labeled HIFAL was also confirmed using RNA pull down assays (Supplementary Fig. 2b). These results suggest that HIFLA may serve as a scaffold to recruit PKM2 to PHD3.

It has been reported that PHD3 binds to and hydroxylates PKM2 in the cytoplasm, and the complex is subsequently translocated into the nucleus to join the HIF-1α transcriptional complex, which enhances and maintains the transactivation activities of HIF-1α^6^. To define the location where HIFAL interacts with PKM2 and PHD3, we employed fractioning of cytoplasm or nucleus and found that HIFAL bound to PKM2 and PHD3 in both the cytoplasm and the nucleus (Fig. 2d, e). Knocking out HIFAL in MDA-MB-231 cells abolished the association of PKM2 with PHD3 and the prolyl hydroxylation of PKM2, which could be rescued by ectopic HIFAL expression (Fig. 2f, g). Furthermore, silencing the exogenous HIFAL in HIFAL-null MDA-MB-231 cells by HIFAL-LNAs dramatically recapitulated the effect of HIFAL knockout (Fig. 2g and Supplementary Fig. 2c). Similarly, targeting the endogenous HIFAL by its LNAs dramatically abolished the binding of PKM2 with PHD3 and the prolyl hydroxylation of PKM2 (Fig. 2h and Supplementary Fig. 2d–f), which was consistent with the shPHD3-mediated decrease in PKM2 prolyl hydroxylation (Supplementary Fig. 2g). Consistently, in vitro catalytic experiment, prolyl hydroxylation of the purified PHD3 towards PKM2 was inefficient, and was dramatically enhanced in the presence of HIFAL (Supplementary Fig. 2h). Previous studies raised the conflicting observations about PHD3 mediated prolyl hydroxylation of PKM2^6,32^. To further validate the PKM2 prolyl hydroxylation, the purified PKM2 protein was incubated with PHD3 and the in vitro hydroxylation experiment was performed. Mass spectrometry (MS) analysis revealed the hydroxylation of Por408, but not Por403 in PKM2 (Supplementary Fig. 2i). Consistently, the Pro408Ala mutant or Pro408/403Ala double mutant, but not Pro403Ala mutant abolished anti-hydroxylation immunoblotting of PKM2 (Supplementary Fig. 2j). On the other hand, silencing HIFAL with LNAs (Supplementary Fig. 2k–n) or knocking out the lncRNA with Cas-9 (Supplementary Fig. 2o) did not affect the mRNA and the protein expression of PKM2, PHD3 and HIF-1α. Additionally, the silencing of PKM2 but not PKM1 decreased the expression of HIF-1α target genes, while the mRNA level of HIF-1α was not affected (Supplementary Fig. 2p). Furthermore, silencing PKM2 or PHD3 did not affect the binding of HIFAL to PHD3 or PKM2 (Supplementary Fig. 2q, r), respectively. These results demonstrate that HIFAL fosters the formation of PKM2/PHD3 complex and promotes PHD3 mediated PKM2 hydroxylation to facilitate HIF-1-related transcription.

To elucidate how HIFAL lncRNA interacts with PKM2 and PHD3, a series of HIFAL deletion mutants were generated to determine the binding motifs of the lncRNA with PKM2 and PHD3. RNA pull-down assay demonstrated that HIFAL mutants retaining nt 1–60 remained the capability to bind to PKM2 as efficiently as the full-length HIFAL, whereas other mutants completely lost their binding capacity (Fig. 2i, j), suggesting that nt 1–60 of HIFAL carries the motif that interacts with PKM2. Additionally, immunoprecipitation (IP) of PKM2 also specifically retrieved HIFAL (nt 1–60) (Supplementary Fig. 3a). The interaction of PKM2 with HIFAL (nt 1–60) was confirmed by electrophoretic mobility shift assay (EMSA) using HIFAL mutant (nt 1–60) (Supplementary Fig. 3b). We next employed two independent sets of software, Mfold^33^ and RNAfold^34^, to predict the secondary structure of HIFAL (nt 1–60), and identified two hairpins within nt 1–60, which we named hairpin A (hA, nt 4–19) and hairpin B (hB, nt 30–57), respectively (Fig. 2k). To further evaluate the contribution of hA and hB to PKM2 binding, we used another set of nt 1–60 fragments, in which each of the hairpins was deleted individually. Either ΔhA (deleting hairpin A) or ΔhB (deleting hairpin B) was capable of binding to PKM2 with similar potency, but the fragment deleting both hairpins (ΔhA + ΔhB) failed to bind PKM2 (Fig. 2l). This was confirmed by EMSA using ΔhA or ΔhB (Supplementary Fig. 3c). Taken together, these results demonstrate a specific interaction between PKM2 and hairpin A or hairpin B of the HIFAL lncRNA.

Similarly, we identified the motif on HIFAL that interacts with PHD3. HIFAL fragments retaining nt 501–560 bound to PHD3 as efficiently as the full-length HIFAL, whereas other fragments completely lost their binding capacity (Fig. 2m, n), suggesting that nt 501–560 of HIFAL harbors the motif that interacts with PHD3. Additionally, immunoprecipitation (IP) of PHD3 specifically retrieved HIFAL (501–560nt) (Supplementary Fig. 3d), and EMSA assays detected a mobility shift delay of HIFAL (nt 501–560) with PHD3 (Supplementary Fig. 3e). Furthermore, one hairpin structure within nt 501–560 was predicted by Mfold and RNAfold, which we named hairpin C (hC, nt 506–532) (Fig. 2o). To determine whether hC is the motif that binds to PHD3, a series of deletion fragments of HIFAL were used. RNA pull-down assay demonstrated that ΔhC (deleting hairpin C) failed to bind to PHD3 (Fig. 2p), which was further confirmed by EMSA using nt501–560 fragment harboring ΔhC (Supplementary Fig. 3f). To further explore how HIFAL recruits PHD3 to PKM2 and induces HIF DNA binding, we constructed a series of PHD3 fragments and found that the PHD3 mutant harboring 115–222aa retained the binding ability with HIFAL (Fig. 2q). Therefore, HIFAL interacts with PKM2 and PHD3 with distinct motifs harboring different hairpin structures to form a HIFAL/PKM2/PHD3 complex.

### HIFAL drives nuclear translocation of PKM2/PHD3 by binding to hnRNPF

**Fig. 3:**
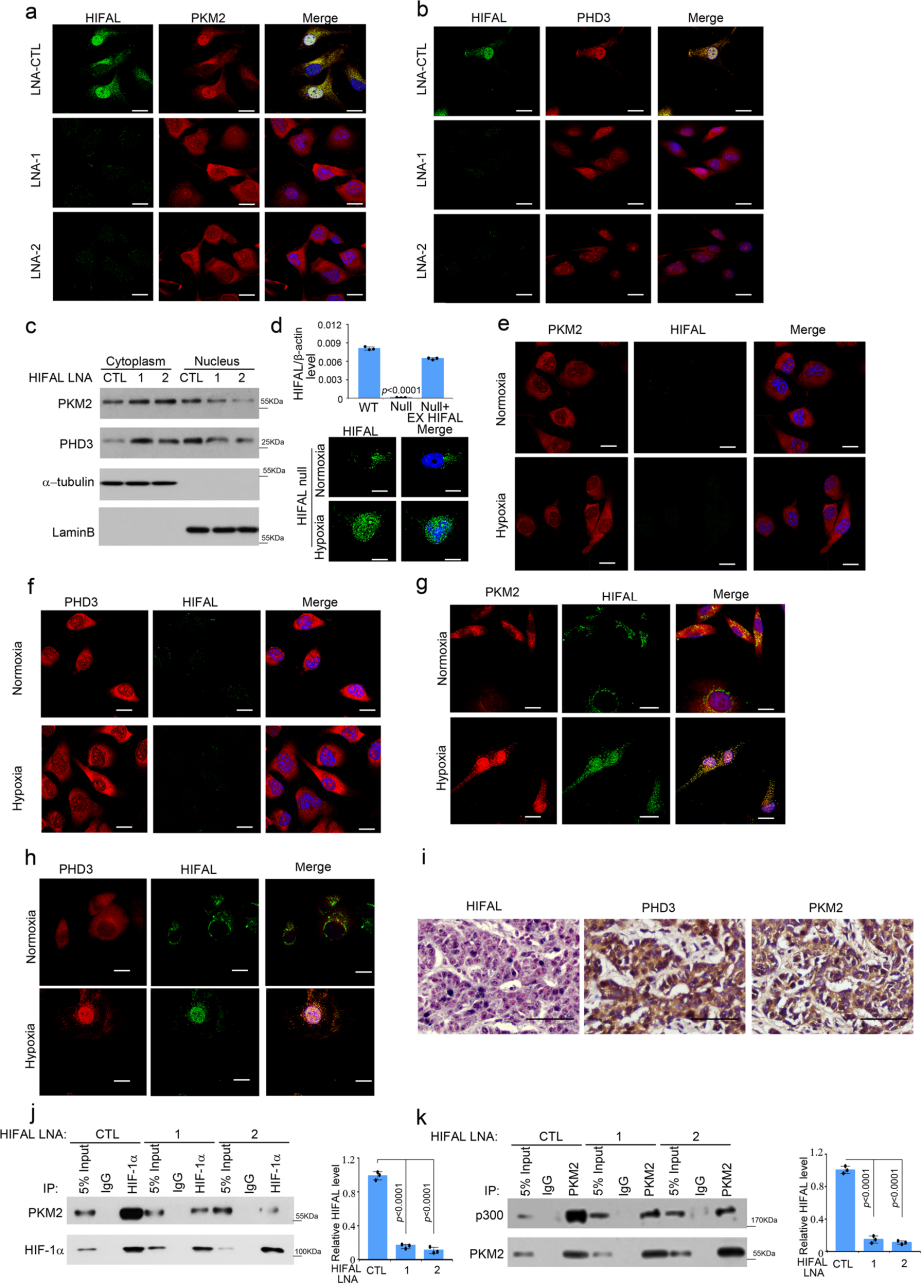
HIFAL nuclear translocation upon hypoxia drives PKM2/PHD3 complex into the nucleus. **a**–**c** Knockdown of HIFAL prevents the nuclear translocation of PKM2 and PHD3. Cells were transiently transfected with HIFAL-LANs for 48 h, and then cultured under hypoxia for 24 h. HIFAL (green) was detected by digoxin labeled probes. PKM2 (**a**) or PHD3 (**b**) (red) were immunostained with antibodies. The cell lysates were fractionated and the cytoplasmic or nuclear expression of PKM2 and PHD3 was evaluated by western blotting (**c**). Scale bars, 10 μM. **d** Exogenous HIFAL translocates into the nucleus upon hypoxia. The HIFAL null MDA-MB-231 cells were transiently transfected with exogenous HIFAL RNA for 48 h and then hybridized with digoxin labeled HIFAL probes (lower panel). The level of endogenous or exogenous (EX) HIFAL in MDA-MB-231WT or HIFAL null cells was detected by qRT-PCR. Scale bars, 10 μM. **e**, **f** HIFAL knockout prevents the nuclear translocation of PKM2 and PHD3 under hypoxia. The HIFAL null MDA-MB-231 cells were cultured under normoxia or hypoxia and then subjected to immunofluorescence staining of PKM2 (**e**) and PHD3 (**f**) and ISH of HIFAL. Scale bars, 10 μm. **g**, **h** Exogenous HIFAL induces the translocation of PKM2 (**g**) and PHD3 (**h**) into nucleus upon hypoxia. Scale bars, 10 μM. **i** Nuclear HIFAL expression correlates with nuclear PKM2 and PHD3 expression in breast cancer tissues. Representative image showed HIFAL expression detected by ISH and PKM2 and PHD3 expression detected by immunohistochemistry. Scale bars, 50 μM. **j**, **k**, Knockdown of HIFAL reduces PKM2 binding with HIF-1α and p300. Cells were transiently transfected with HIFAL-LANs for 48 h, and then cultured under hypoxia. Knockdown of HIFAL reduces PKM2 binding with HIF-1α (**j**) and p300 (**k**). The right panel shows the relative HIFAL level in the MDA-MB-231 cells. For **d** (upper), **j** (right), **k** (right), bar graphs represent means ± SD of experimental triplicates, and *p* values were determined by two-sided unpaired t-test. Source data are provided as a Source Data file.

Since we showed that HIFAL was mainly upregulated in the nucleus under hypoxia and could bind with PKM2 and PHD3, we hypothesized that HIFAL assisted nuclear translocation of the PKM2/PHD3 complex under hypoxic conditions. To this end, we compared the dynamics of HIFAL expression in the cytoplasm/nucleus and the nuclear translocation of PKM2/PHD3 complex under hypoxia. We found that following hypoxia, the nuclear level of HIFAL increased in consistence with the nuclear translocation of the PKM2/PHD3 complex (Supplementary Fig. 3g, h). More importantly, silencing HIFAL expression by its targeting LNA significantly reduced the nuclear translocation of the PKM2/PHD3 complex under hypoxia (Fig. 3a–c and Supplementary Fig. 3i), suggesting that HIFAL was required for the translocation of the PKM2/PHD3 complex into the nuclei.

To further investigate whether HIFAL translocated into the nucleus together with the complex of PKM2/PHD3, we directly transfected biotin-labeled exogenous HIFAL lncRNA into HIFAL-null MDA-MB-231 cells^35^ (Fig. 3d). The exogenous HIFAL was present in the cytoplasm after transfection under normoxia and translocated into the nuclei upon hypoxia, confirming that hypoxia enhanced the nuclear translocation of HIFAL lncRNA (Fig. 3d). Moreover, silencing PKM2 or PHD3 did not affect the nuclear transfer of HIFAL, suggesting that the translocation of HIFAL was independent of PKM2 or PHD3 (Supplementary Fig. 3j, k). More importantly, in HIFAL null cells, the PKM2/PHD3 complex could not be transferred into the nuclei under hypoxia (Fig. 3e, f), while transfection of exogenous HIFAL retrieved the hypoxia-induced nuclear translocation of PKM2/PHD3 complex (Fig. 3g, h). These observations were further consolidated by the in situ hybridization for HIFAL and immunohistochemistry for PKM2 or PHD3 in breast cancer tissues, which showed co-localization of HIFAL with PKM2 and PHD3 in the nuclei of cancer cells (Fig. 3i and Supplementary Fig. 3l, m). Furthermore, knocking down HIFAL efficiently repressed the formation of the transcriptional complex containing HIF-1α and p300 (Fig. 3j, k). Together, our data suggest that HIFAL is essential for the nuclear translocation of PKM2/PHD3 complex and the formation of HIF-1 transcriptional complex driven by the hydroxyl-PKM2 in the nucleus.

**Fig. 4:**
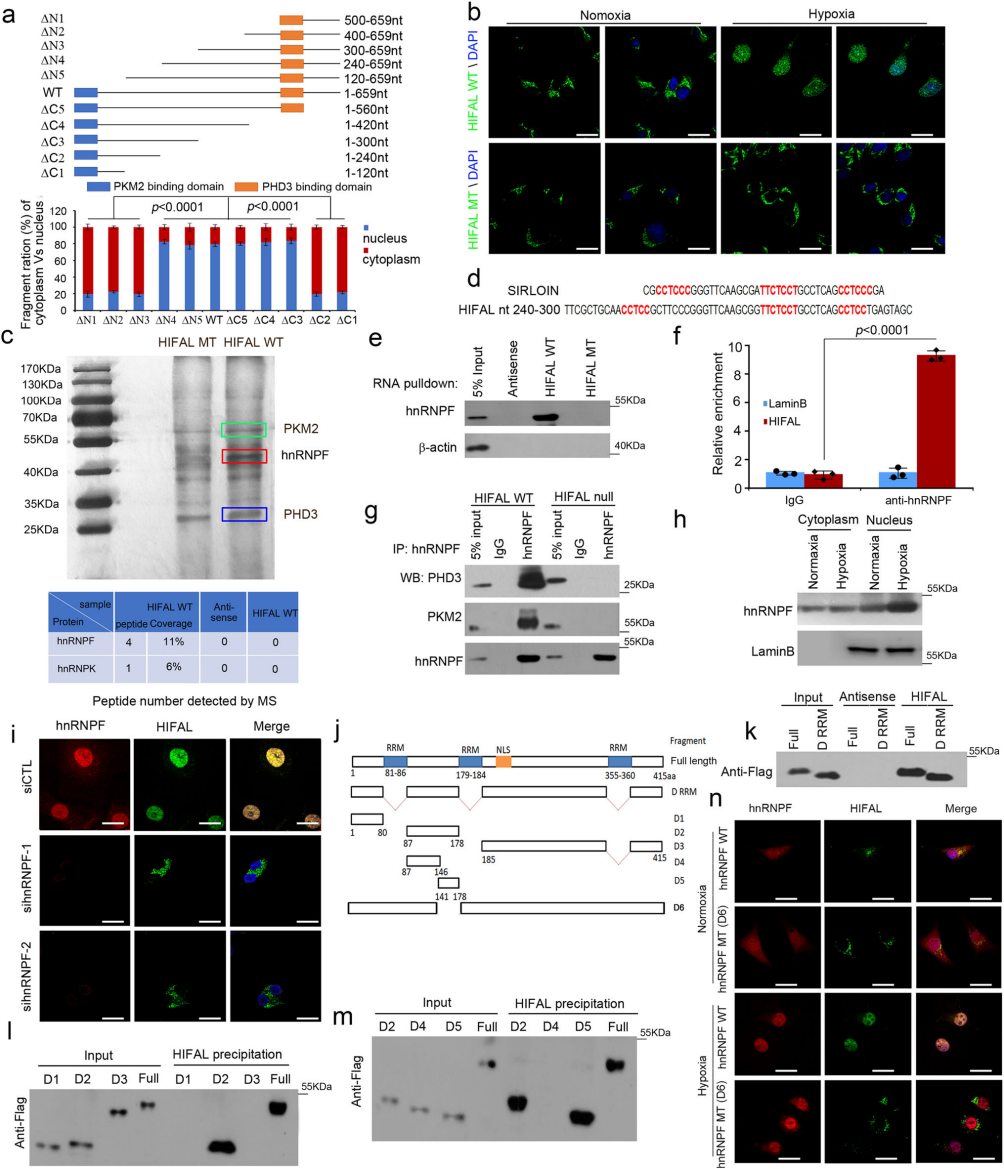
HIFAL translocates into the nucleus via binding with hnRNPF. **a**, **b** HIAFL fragment nt 240–300 is essential for its nucleus translocation. **a** Various HIFAL fragments were transfected into HIFAL null MDA-MB-231 cells. RNA in the cytoplasmic or nuclear fractions was isolated for HIFAL detection. *P* values were determined Ordinary one-way ANOVA with Dunnett’s post hoc test. Bar graphs represent means ± SD of fifteen random fields. **b** Deletion of HIFAL fragment nt 240–300 abrogates its translocation into the nucleus upon hypoxia. The biotin-labeled exogenous wild type or mutated HIFAL without nt 240–300 was transfected into HIFAL null MDA-MB-231 cells, and detected by FITC labeled anti-biotin antibody. Scale bars, 10 μM. **c** Wildtype but not mutated HIFAL binds to hnRNPF, as detected by mass spectrum identifications. The peptide number of hnRNPF or hnRNPK detected by mass spectrum was shown in the table (lower). **d** The region for HIFAL nuclear translocation contains similar RNA sequence with SIRLOIN. The conserved sequence of SIRLOIN or HIFAL nt 240–300 was highlighted in red. **e**, **f** HnRNPF binds with WT but not mutated HIFAL without the nuclear translocation region (nt 240–300), as detected by RNA pulldown (**e**) and RNA immunoprecipitation assay (**f**). Bar graphs represent means ± SD of experimental triplicates. *P* values were determined by two-sided unpaired t-test. **g** Binding of PKM2 or PHD3 with hnRNPF depends on HIFAL. The immunoprecipitation of hnRNPF was performed in HIFAL WT or null MDA-MB-231 cells. **h**, Hypoxia induces hnRNPF overexpression and nuclear translocation. **i** Knockdown of hnRNPF abolishes HIFAL nuclear translocation. HIFAL (green) was detected by digoxin labeled probes. Scale bars, 10 μM. **j** Diagram of the full length hnRNPF and its serial truncated mutants. **k** The RRM truncated hnRNPF mutant retains the ability of binding with HIFAL. Full length or the RRM deletion mutant (D RRM) of hnRNPF was transfected into HEK293 cells for HIFAL RNA-pull down assay. **l**, **m** HnRNPF 141–178aa region contains the HIFAL binding domain. **n** hnRNPF mutant without the HIFAL binding-domain cannot induce the nuclear translocation of HIFAL. The hnRNPF null cells were transiently transfected with hnRNPF WT or D6 mutant and cultured under hypoxia. Scale bars, 10 μM. Source data are provided as a Source Data file.

It has been shown that lncRNA motifs interacting with nuclear localization proteins may determine the nuclear localization of lncRNAs. JMJD5, a Jumonji C domain-containing dioxygenase, has been reported to enhance nuclear localization of PKM2/PHD3 complex under hypoxic or normoxic conditions^15^. To rule out whether the effect of HIFAL was dependent on JMJD5, we silenced JMJD5 expression in the MD-MBA-213 cells and found that it did not affect HIFAL translocation into the nuclei upon hypoxia (Supplementary Fig. 3n, o). Therefore, we further investigated whether HIFAL may harbor motifs that bind to nuclear proteins responsible for its nuclear translocation under hypoxia and thus bring the HIFAL/PKM2/PHD3 complex into the nucleus. To this end, we generated a series of truncated HIFAL fragments and transfected them into HIFAL-null MDA-MB-231 cells (Fig. 4a). Under hypoxic conditions, HIFAL fragments harboring nt 240–300 were mainly located in the nucleus, while those without nt 240–300 were found in the cytoplasm (Fig. 4a). Direct transfection of the biotin labeled wild-type HIFAL RNA, but not the mutant RNA that lacks nt 240–300, resulted in nuclear localization of the lncRNAs in the HIFAL-null MDA-MB-231 cells under hypoxic conditions (Fig. 4b and Supplementary Fig. 3p). These data suggested that a HIFAL motif within nt 240–300 is responsible for its nuclear translocation under hypoxia.

To further identify the nuclear-translocating protein that binds to HIFAL nt 240–300, we performed RNA pull-down and subjected the HIFAL associated proteins to mass spectrometry. A protein about 50 kDa, identified as hnRNPF, specifically bound to wild-type, but not mutant HIFAL without nt 240–300 (Fig. 4c). More interestingly, we found that HIFAL^nt240–300^ contained the SIRLOIN like sequence (Fig. 4d), which was shown to bind to hnRNPK and was responsible for the nuclear translocation of lncRNAs^36^. Indeed, RNA pull-down using a biotinated-RNA probe and RNA-IP using an anti-hnRNPK or an anti-hnRNPF antibody confirmed that either hnRNPK or hnRNPF could bind to the wild-type, but not the mutant HIFAL (Fig. 4e, f and Supplementary Fig. 4a, b). However, hnRNPF showed higher binding capacity than hnRNPK with HIFAL in both the RNA pull-down and RNA immunoprecipitation assays (Fig. 4e, f and Supplementary Fig. 4a, b). Furthermore, both hnRNPK and hnRNPF could form a complex with PKM2 or PHD3 only in the presence of HIFAL, but could not bind with PKM2 or PHD3 in the HIFAL-null cells (Fig. 4g, and Supplementary Fig. 4c). Since HIFAL was introduced into the nucleus under hypoxia, we further evaluated whether the expression of hnRNPK and hnRNPF was influenced by hypoxia. HnRNPF expression was upregulated in the nucleus under hypoxia, while hnRNPK was not (Fig. 4h and Supplementary Fig. 4d), suggesting that the hnRNPF was induced and translocated into the nucleus upon oxygen deprivation. More importantly, silencing hnRNPF by siRNAs inhibited the nuclear translocation of HIFAL under hypoxia (Fig. 4i and Supplementary Fig. 4e), while knocking down hnRNPK only slightly suppressed its nuclear translocation (Supplementary Fig. 4f, g).

Although hnRNPF was shown to bind the G-rich sequence via the RRM domains^37^, no G-rich sequence within the nuclear localization sequence (nt 240–300) of HIFAL was identified, suggesting that hnRNPF may bind HIFAL with a different domain to facilitate the nuclear import of PKM2/PHD3 complex. To identify which of the hnRNPF domains was responsible for HIFAL binding, a serial of hnRNFP fragments were generated (Fig. 4j). We found that the hnRNFP mutant without the G-rich binding domain retained the ability of binding with HIFAL (Fig. 4k), and the hnRNFP fragment of 141–178 aa was essential for the binding of hnRNPF with HIFLA (Fig. 4l, m). In addition, we confirmed the binding of HIFAL with truncated hnRNPF (141–178 aa) by EMSA experiment (Supplementary Fig. 4h). Consistently, transfection of hnRNPF mutant (D6) without the HIFAL-binding domain (141–178 aa) could not induce nuclear translocation of HIFAL in the hnRNPF knockout cells (Fig. 4n and Supplementary Fig. 4i). To confirm whether hnRNPF mobilized HIFAL lncRNA to the nucleus, we transfected exogenous HIFAL into hnRNPF KO or WT cells (Supplementary Fig. 5a, b) and found that the exogenous HIFAL RNA could be translocated into the nucleus of the hnRNPF WT cells, but not for the hnRNPF KO cells, under hypoxia, and the lncRNA was thus accumulated in the cytoplasm (Supplementary Fig. 5a, b). Collectively, hypoxia-induced the expression of hnRNPF to mediate nuclear translocation of the HIFAL/PKM2/PHD3 complex.

### HIF-1 transcriptional complex induces HIFAL expression

**Fig. 5:**
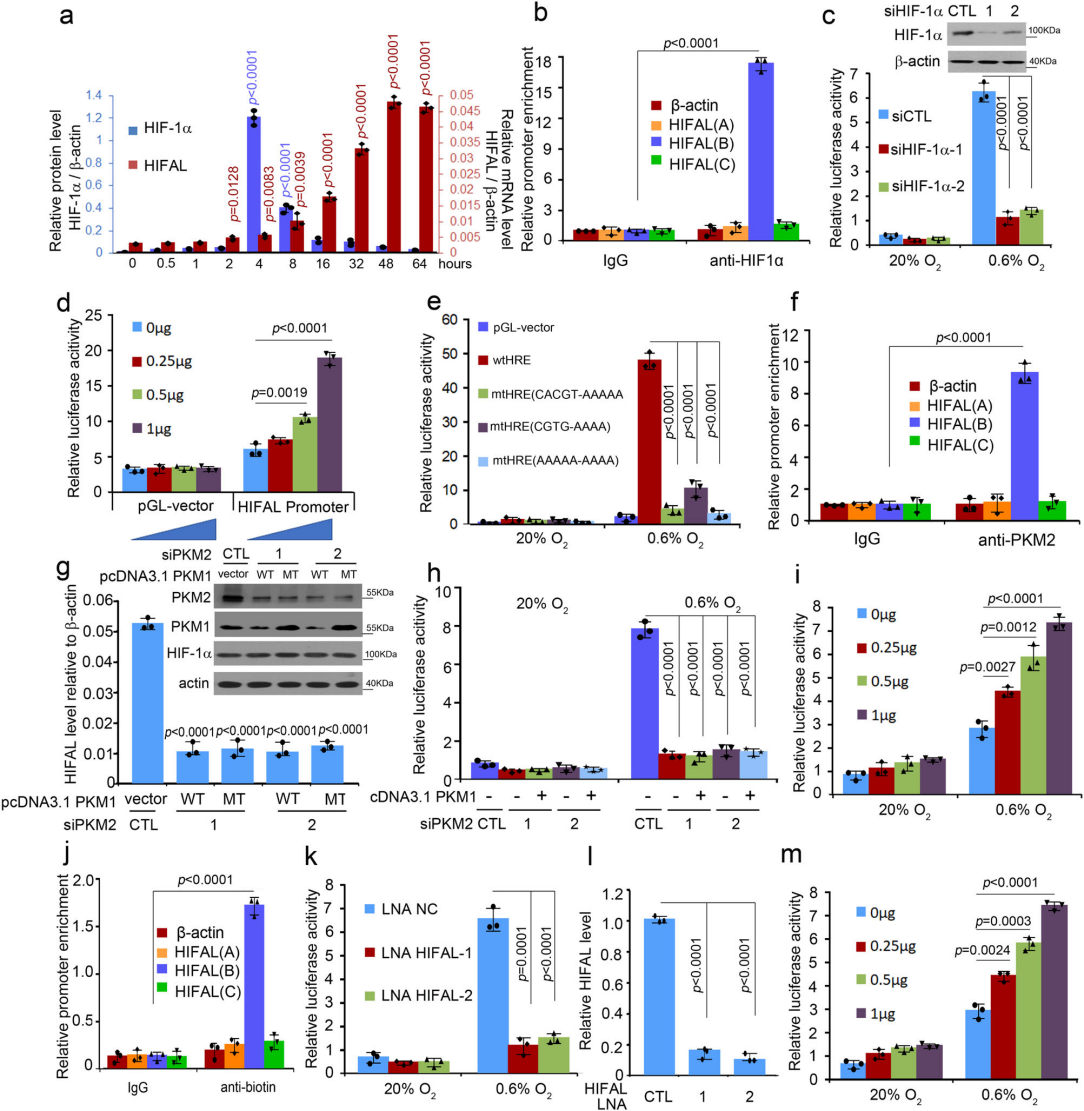
HIFAL transcription is activated by HIF-1α transcriptional complex containing HIFAL itself in a feed-forward loop. **a** HIFAL transcription is not in line with HIF-1α protein level after hypoxia. **b** HIF-1α binds to the HIFAL promoter under hypoxia. MDA-MB-231 cells were cultured under hypoxia for 24 h, then collected for ChIP assay. Primers spanning the HIFAL locus were used to confirm the binding specificity of HIF-1α at the promoter/HRE element. [HIFAL (B)], −916~−719bp [HIFAL (A)], and +578~+749 bp [(HIFAL(C)] of the promoter/HRE element. The β-actin promoter was used as the negative control. **c** Hypoxia-induced HIFAL transcription is inhibited by HIF-1α knockdown. MDA-MB-231 cells transiently transfected with siHIF-1α were collected to detect the HIFAL promoter transcription activity under hypoxia. **d** HIF-1α activates HIFAL transcription in a dose-dependent manner under hypoxia.HIF-1α plasmid (0, 0.25, 0.5, 1 μg) was transfected into MCF-7 cells for the detection of HIFAL transcriptional activity. **e** HRE mutation abolished the hypoxia induced HIFAL transcription activity. MDA-MB-231cells transfected with indicated wild type or mutant HIFAL promoter were collected to detect the promoter transcription activity. **f** PKM2 binds to the HIFAL promoter under hypoxia. MDA-MB-231 cells were collected for ChIP analyses. **g** Knocking down PKM2 reduces HIFAL level upon hypoxia, which could not be rescued by PKM1 overexpression. **h** PKM2 siRNA decreases the hypoxia-induced HIFAL promotor transcriptional activity, which could not be rescued by PKM1 overexpression. **i** PKM2 activates the HIFAL transcription in a dose-dependent manner under hypoxia. PKM2 plasmid (0, 0.25, 0.5, 1 μg) was transfected into MCF-7 cells for the detection of HIFAL transcriptional activity. **j** HIFAL binds to HIFAL promoter under hypoxia. MDA-MB-231 cells were transfected with biotinlabeled HIFAL or antisense, then collected for ChIP analyses. **k**, **l** Knocking down HIFAL decreases the hypoxia-induced HIFAL promotor transcriptional activity. The transcription activity of HIFAL promotor was shown in (**k**). The HIFAL levels are shown in (**l**). **m** HIFAL activates the transcriptional activity of HIFAL promotor in a dose-dependent manner under hypoxia. HIFAL plasmid (0, 0.25, 0.5, 1 μg) was used for the transfection into MCF-7 cells. For **b**, **f**, **j**, *p* < 0.0001 versus IgG precipitation. Graphs show means ± SD of experimental triplicates, *p* values were determined by two-sided unpaired t-test (**a**–**m**). Source data are provided as a Source Data file.

Given that HIFAL expression was induced by hypoxia, we sought to determine whether HIFAL transcription was regulated by HIF-1α. Similar to other HIF-1α target genes (Fig. 1a), HIFAL level began to increase at 4 h following hypoxia and plateaued at 48 h, whereas HIF-1α protein level peaked at 4 h post hypoxia and then steadily decrease with time (Fig. 5a). Furthermore, the ChIP assay using an anti-HIF-1α antibody demonstrated the enrichment of HIF-1α at HIFAL promoter (Fig. 5b). Consistently, the luciferase reporter assay showed that knocking down HIF-1α expression almost abolished the hypoxia-induced transcriptional activities of HIFAL promoter (Fig. 5c). On the other hand, enforced expression of HIF-1α in breast cancer cells increased the transcriptional activity of HIFAL promoter in a dose-dependent manner (Fig. 5d). Moreover, hypoxia treatment failed to enhance the transcriptional activities of HIFAL promoters with HRE mutation, which lost its binding affinity with HIF-1α (Fig. 5e). Together, these data suggested that HIFAL transcription is driven by HIF-1 complex.

Since the HIFAL/PKM2/PHD3 complex is involved in HIF-1α driven transcription, we further explored the role of the complex in HIFAL transcription. ChIP assay analysis confirmed the binding of PKM2 to HIFAL promoter (Fig. 5f). PKM2 knockdown decreased HIFAL expression (Fig. 5g) and almost abolished the hypoxia-induced HIFAL promoter activities determined by luciferase reporter assays (Fig. 5h), which could not be rescued by enforced expression of PKM1, another member of the PKM family. Accordingly, siPKM2 decreased the expression of GLUT1, HKII, LDHA, PDK1, which could not be rescued by ectopic expression of PKM1 with siRNA-resistant mutation (Supplementary Fig. 2p). In addition, PKM2 overexpression enhanced the promoter activity of HIFAL in a dose-dependent manner (Fig. 5i). To explore whether HIFAL lncRNA promotes its own transcription, a biotin-labeled HIFAL lncRNA or an anti-sense control was directly introduced into cells by lipofectamine^35^, and a ChIP assay was performed using an anti-biotin antibody. Interestingly, HIFAL promoter DNA was enriched by approximately 20 folds as compared with the anti-sense control in the ChIP assay (Fig. 5j). Moreover, silencing HIFAL expression abolished the hypoxia-induced transcriptional activity of HIFAL promoter as determined by luciferase reporter assays (Fig. 5k, l), while enforced expression of HIFAL in the breast cancer cells increased the transcriptional activity of HIFAL promoter (Fig. 5m). These results suggested that HIFAL lncRNA enhances its own transcription driven by HIF-1α under hypoxic condition in a feed-forward manner by forming a stable HIFAL/PKM2/PHD3 complex at the HRE of its own promoter.

### HIFAL promotes the assembly of HIF-1 transactivation complex and glycolysis

**Fig. 6:**
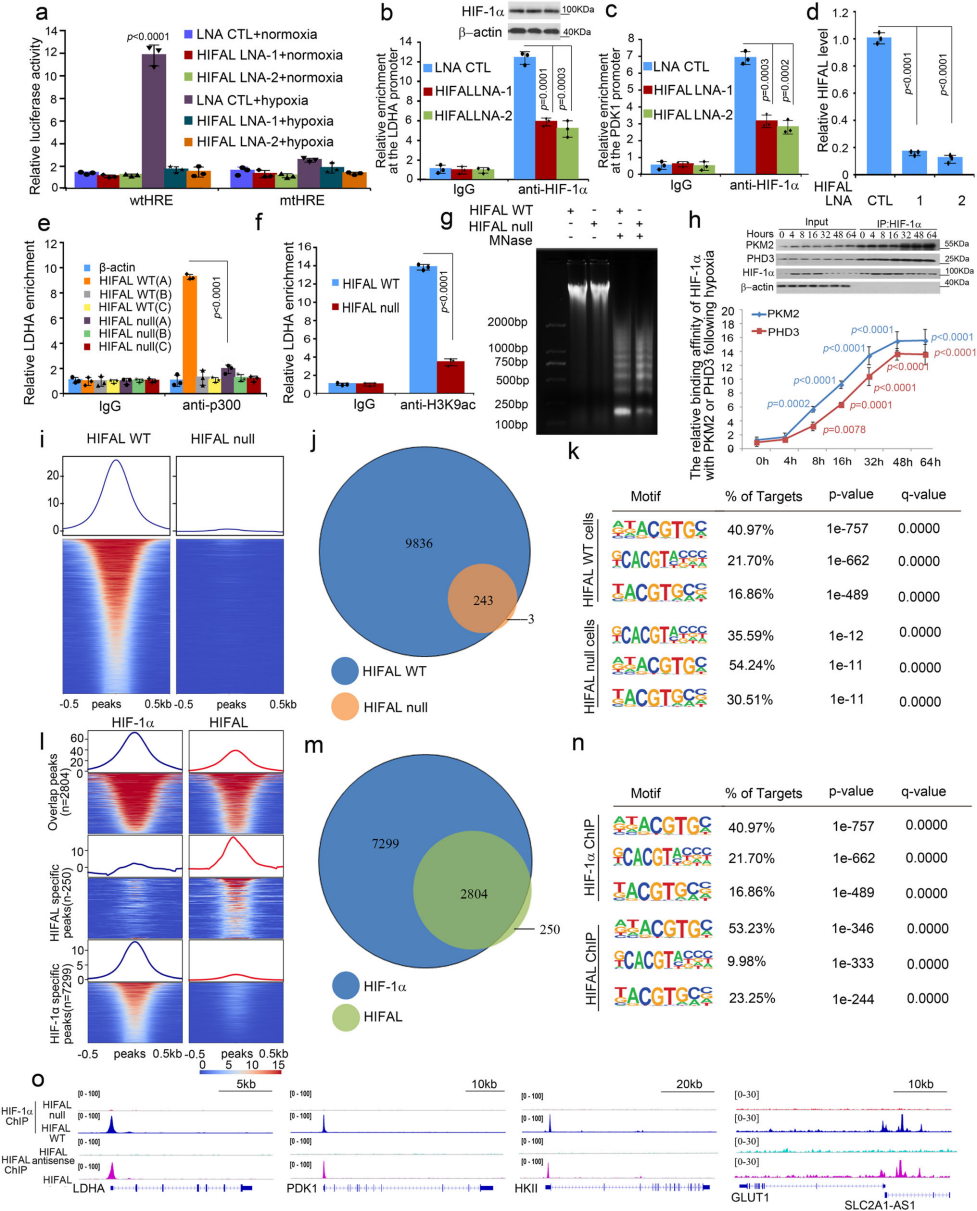
HIFAL is essential for the HIF-1α transcription activities. **a** The HRE element transcription activity is inhibited by HIFAL-LNA upon hypoxia. **b**–**d** HIFAL knockdown decreases the binding of HIF-1α to the promoter of *LDHA* (**b**) and *PDK1* (**c**). HIFAL levels are shown in (**d**). **e**, **f** HIFAL knockout decreases the binding of p300 to the promoter of LDHA (**e**) and the histone H3K9 acetylation on the LDHA promoter (**f**). Primers spanning the *LDHA* locus [*LDHA*(A)], −873~−697bp[*LDHA*(B)] and +631~+823 bp[(*LDHA*(C)] were used. **g** HIFAL knockout decreases the chromatin sensitivity to micrococcal nuclease. **h**, The binding kinetics of HIF-1α to PKM2 and PHD3 keeps increasing after hypoxia in the presence of HIFAL. HIF-1α co-immunoprecipitation (Co-IP) assay was performed at indicated time points after hypoxia and normalized to the corresponding HIF-1a protein levels. **i** Distribution profiles of HIF-1α binding genes flanking the transcription start sites (TSS) in HIFAL WT or null MDA-MB-231 cells under hypoxia as determined by ChIP-seq. The 10 kb regions centered on all the TSSs of HIF-1α binding genes were shown. Red indicates enrichment, while blue indicates no enrichment. **j**, HIF-1α cannot bind to most of its target genes in the HIFAL null condition. **k** Binding motifs enriched within 1 kb of the promoter region. **l** A dual heatmap illustrating the co-occupancy peaks of HIF1a and HIFAL ChIP-seq in MBA-MD-231 cells. Peaks were rank from the highest amount of HIF-1α/HIFAL to the lowest, and were flanking with ±0.5 kb sequence of TSS. Red indicates enrichment, while blue indicates no enrichment. **m** The enrichment peaks of HIFAL and HIF-1α overlap significantly. Under the most stringent criteria, 2804 out of the 3054 (91.8%) HIFAL peaks are also bound by HIF-1α. **n** Binding motifs enriched within ±0.5 kb of the promoter region. HIF-1α and HIFAL shares the most frequent target sequence. **o** Representative HIF-1α target glycolysis gene loci are similarly enriched by both HIF-1α and HIFAL in HIFAL WT or null MDA-MB-231 cells. Graphs show means ± SD of experimental triplicates, *p* values were determined by two-sided unpaired t-test (**a**–**f**, **h**). Source data are provided as a Source Data file.

The above data revealed a central regulatory role of HIFAL lncRNA in HIF-1α-driven transcription and anaerobic glycolysis. To further evaluate the contributions of HIFAL in HIF-1α mediated transactivation, we employed luciferase reporter plasmids with HRE promoters. As expected, the luciferase activities driven by wild-type, but not the mutant HREs were tremendously increased under hypoxia, but were abrogated by silencing HIFAL expression with LNAs (Fig. 6a), suggesting that HIFAL is involved in HIF-1α-mediated transactivation. It is well established that PHD3/PKM2 assists to recruit HIF-1α to the HREs of target gene promoters upon hypoxia to initiate their transcription^6^, and our above data showed that HIFAL formed a stable complex with PHD3/PKM2 and induced the complex into the nucleus. Therefore, we further explored whether HIFAL contributes to the enrichment of HIF-1α at its target gene promoters by ChIP assays with an anti-HIF-1α antibody. As a result, knocking down HIFAL expression significantly reduced HIF-1α occupancy at the promoters of *LDHA* and *PDK1* genes under hypoxia, but did not affect HIF-1α enrichment at the promoter of β-actin (Fig. 6b–d and Supplementary Fig. 6a). In line with these observations, knocking out PKM2 or PHD3 also resulted in the decreased enrichment of HIF-1α to its target genes (Supplementary Fig. 6b–e). PKM2 dephosphorylates phosphoenolpyruvate to pyruvate with an ATP generation. To determine whether PKM2 enzymatic activity is required for the HIFAL mediated feed-forward loop of HIF-1α transactivation, the catalytically inactive PKM2(K270M) was used. Luciferase reporter assays demonstrated that PKM2(K270M) functioned similarly as WT PKM2, which increased HIF-1α transcriptional activity in the presence of HIFAL (Supplementary Fig. 6f), suggesting the enzymatic activity of PKM2 was not essential for HIFAL-mediated feed-forward loop of HIF-1α transactivation. Meanwhile, a series of PKM2 mutants were generated to investigate the effect of prolyl hydroxylation of PKM2 on HIFAL-mediated HIF-1α transactivation. The overexpression of Pro408Ala or Pro403/408Ala mutants could not increase the HIFAL-mediated HIF1α transactivation, whereas Pro403Ala mutant, as well as WT PKM, could do so with similar efficiency. These results suggested that the prolyl hydroxylation of Pro408 in PKM2 was essential for the HIFAL-mediated HIF1α transactivation (Supplementary Fig. 6g).

Furthermore, we investigated whether HIFAL contributes to the recruitment of p300, a histone acetyltransferase, to the HREs of HIF-1α target genes, since it was reported that p300 serves as a coactivator of HIF-1 transactivation and is recruited to HREs by PKM2^6,38^. ChIP assays revealed that p300 efficiently bound to the HER of *LDHA* promoter under hypoxia, which was greatly suppressed by HIFAL KO (Fig. 6e). Moreover, HIFAL KO dramatically reduced the p300-mediated histone H3 acetylation at lysine-9 (H3K9ac) at the HRE of *LDHA* promoter under hypoxia (Fig. 6f). To further explore whether HIFAL-mediated p300 recruitment contributes to loosen the chromatin and facilitate the transcription of HIF-1α target genes as reported elsewhere^39^, micrococcal nuclease chromatin sensitivity assays in the DNA agarose electrophoresis showed that knocking out HIFAL prevented chromatin degradation upon micrococcal nuclease treatment, suggesting that HIFAL could increase the open chromatin region for transcription (Fig. 6g). In agreement with HIFAL-mediated HIF-1α binding to its target genes (Fig. 1k, l), the binding of PKM2 or PHD3 to HIF-1α (Fig. 6h) in the HIFAL WT cells continuously increased from 4 h following hypoxia and reached the plateau around 48 h (Fig. 6h). However, in the HIFAL null cells, the binding kinetics of PKM2 and PHD3 to HIF-1α was not increased after hypoxia (Supplementary Fig. 6h). These data suggested that HIFAL promotes HIF-1-driven transactivation by introducing the PKM2/PHD3 complex into the nucleus and fostering the formation of HIF-1 transcriptional complex at the HRE of HIF-1 target gene promoters. Furthermore, RNA pull-down assay displayed that HIFAL could bind to HIF-1α in nuclear fraction, but not in cytoplasmic fraction under hypoxia. When knocking down PKM2, the interaction of HIFAL with HIF-1α in nuclear extraction reduced dramatically (supplementary Fig. 6i, j). In addition, knocking down PKM2 reduced the enrichment of HIFAL in the promotors of HIF-1 target glycolytic genes (supplementary Fig. 6k). These results suggested that HIFAL was recruited to the HIF-1α targeting promoter and enhanced the HIF-1α mediated transcription in a PKM2 dependent manner.

To evaluate the impact of HIFAL on the transcription activity of HIF-1α, a genome-wide occupancy of HIF-1α was determined by ChIP-seq in either the HIFAL WT or null MDA-MB-231 cells under hypoxia. Knocking out HIFAL resulted in 97% loss of the HIF-1α binding to approximately 10,000 of its target genes (Fig. 6i–k). The ChIP-seq also revealed the most frequent binding motifs of HIF-1α genome-wide in HIFAL WT cells. Knocking out HIFAL resulted in the changed preference of HIF-1α binding to these motifs (Fig. 6k). In addition, more than 90% of the HIFAL target genes were included in the HIF-1α target genomes, as evaluated by genome-wide HIF-1α and HIFAL ChIP-seq assays (Fig. 6l, m), confirming the similarity of the most frequent binding sequences between HIF-1α and HIFAL (Fig. 6n). We further validated the binding of HIFAL on HIF-1α targeting loci revealed by ChIP-seq. 11 of 16 selected HIF-1α targeting loci could be confirmed by ChIP-PCR (Supplementary Fig. 6l). Focusing on the HIF-1 target glycolytic genes, including *LDHA, PDK1, GLUT1, and HKII*, their occupancy pattern of ChIP-seq by HIF-1α and HIFAL were very similar (Fig. 6o).

**Fig. 7:**
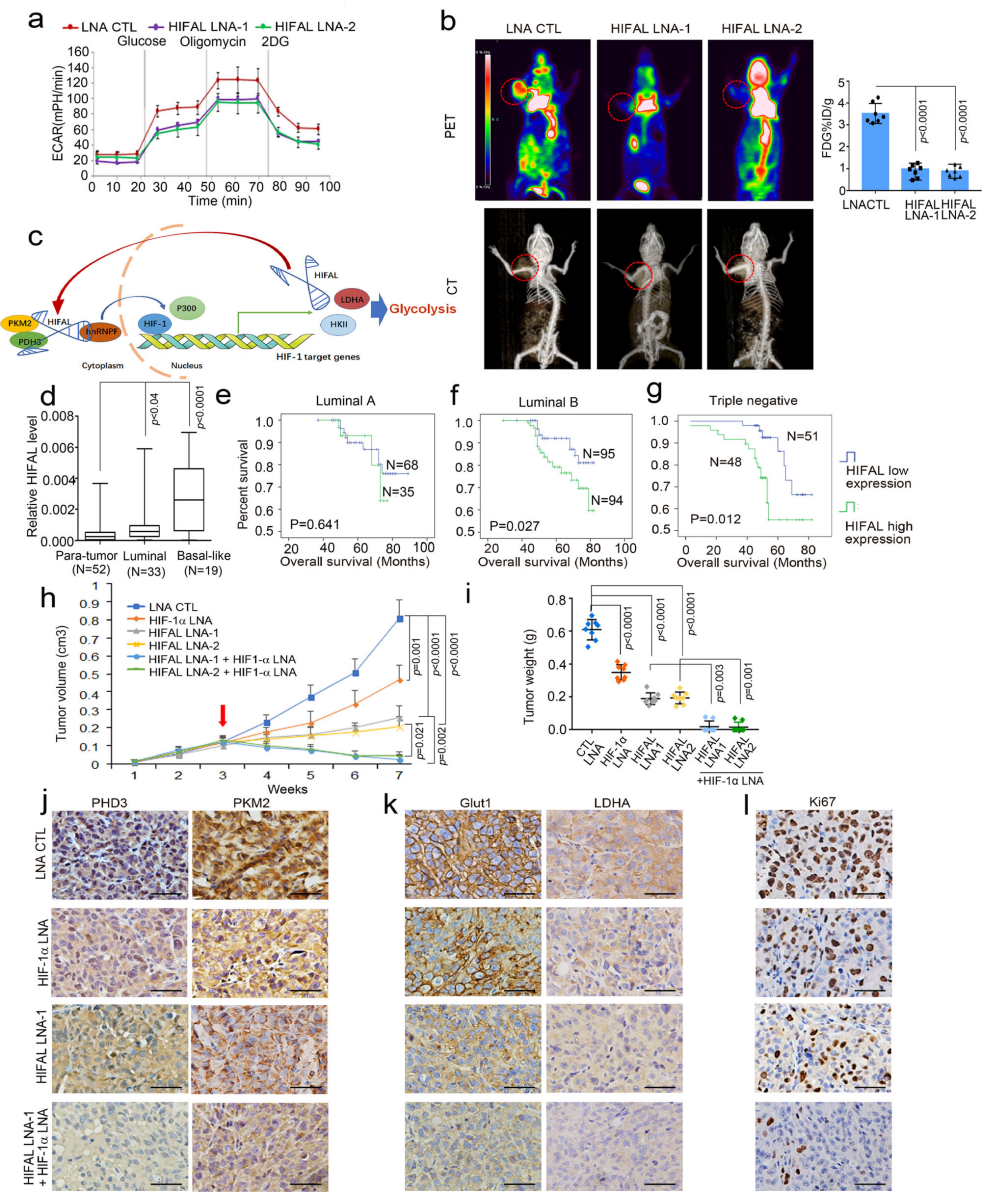
HIFAL is essential for HIF-1α dependent glycolysis and knockdown of HIFAL and HIF-1α synergistically reduces tumor growth in vivo. **a** HIFAL knockdown decreases glycolysis in vitro. ECAR was measured, including the glycolytic reserve. The curves are means ± SD of experimental triplicates. **b** Knockdown of HIFAL decreases breast tumor glycolysis. Mice were scanned for 18 FDG-uptake by FDG-PET and CT after the treatment of control and HIFAL LNAs. The MDA-MB-231 tumors were circled with dashed red lines in the scan images. The 18F-FDG-uptake in 7 mice per group were shown in the right plots. **c** A schematic model demonstrates the role of HIFAL in assembling the HIF-1 transactivation complex and drives the transcription of HIF-1 target genes. HIFAL recruits PKM2/PHD3 complex, introduces their nuclear transportation by binding with hnRNPF and enhances the transcription of HIF-1 target genes by assembling the HIF-1α co-activators. Reciprocally, HIF-1α induces HIFAL transcription, which forms a positive feed-forward loop to maintain the HIF-1α transactivating activities. **d** HIFAL is overexpressed in breast cancer tissues, especially in basal-like cancers, as detected by q-PCR in 52 paired tumor and para-tumor tissue. **e**–**g** High HIFAL level correlates with poor overall survival of breast cancer patients in luminal A (**e**), luminal B cancers (**f**) and basal like cancers (**g**), Kaplan–Meier, log-rank test. **h**, **i** Knockdown of HIFAL and HIF-1α synergistically reduces tumor growth of MDA-MB-231 xenografts in nude mice. HIFAL-LNAs and/or HIF-1α-LNAs were injected intraperitoneally after the tumor volumes reached 100 mm^3^. The growth curves (**h**) and tumor weights (**i**) are means ± SD of 8 mice in each group, *p* values were determined by one-way ANOVA + Dunnett’s post hoc tests. **j** Knockdown of HIFAL suppresses nuclear translocation of PHD3 and PKM2 in xenografts detected by immunohistochemistry. **k**, **l** Knockdown of HIFAL and HIF-1α decreases glycolysis and the proliferation of tumor cells. Immunohistochemistry for PHD3, PKM2, GLUT1, LDHA, and ki67 was performed in xenografts (**j**–**l**). Scale bars, 20 μM. *P* values were determined by two-sided unpaired t-test (**b**, **d**). Source data are provided as a Source Data file.

To evaluate the functional roles of HIFAL in HIF-1-mediated glycolysis, the extracellular acidification rate (ECAR) using a seahorse instrument was employed. Under the hypoxic condition, HIFAL knockdown in MDA-MB-231 cells significantly reduced the ECAR that was mainly determined by lactate production from glycolysis (Fig. 7a). In consistent, the glucose uptake and the lactate production were also reduced when HIFAL expression was knocked down by LNAs in breast cancer cells under hypoxia (Supplementary Fig. 6m–p), whereas enforced expression of HIFAL increased the glucose uptake and the lactate production in these cells (Supplementary Fig. 6q–r). To further evaluate the fundamental role of HIFAL in HIF-1-mediated glycolysis, the glucose uptake in breast tumors as indicated by 18F-FDG was determined by PET-CT. In an immunocompromised mouse model bearing MDA-MB-231 breast tumor xenografts, knockdown of HILFA by LNAs dramatically reduced the uptake of 18F-FDG scanned by PET-CT as compared with the tumors treated with the antisense control (Fig. 7b). The cell viability assay also showed that knockdown of HIFAL in MDA-MB-231 cells decreased the in vitro cell viability under hypoxia, but did not affect cell proliferation under normoxia (Supplementary Fig. 6s). However, enforced expression of HIFAL in MCF-7 cells did not affect the in vitro cell viability in normoxia (Supplementary Fig. 6t). Together, these findings suggest that HIFAL enhances glycolysis upon hypoxia.

### Targeting both HIFAL and HIF-1α synergistically abolishes tumor growth

To further evaluate the clinical significance of HIFAL in breast cancer progression, we performed in situ hybridization for HIFAL expression level in 52 cases of breast cancer and paired normal tissues. HIFAL expression was significantly lower in the normal breast tissue compared to tumor tissues (Fig. 7d). Notably, abundant HIFAL expression was observed in basal-like cancers from our cohort and TCGA datasets (Fig. 7d and Supplementary Fig. 7a–d). High-level HIFAL was associated with poor outcome in TCGA datasets (Supplementary Fig. 7e). Multivariate Cox regression analysis indicated that high HIFAL expression is an independent prognostic factor for poor survival of breast cancer patients (*p* < 0.01, Supplementary Table 2). Moreover, the stratified analysis revealed that high-level HIFAL expression in breast cancer tissues was significantly associated with advanced disease staging, higher histopathological grading, enhanced tumor size, as well as lymph node and distant metastasis in both luminal and triple-negative breast cancers (Table S3, 4, 5). In addition, the high HIFAL level correlated with poor overall survival in triple-negative and luminal B breast cancers but not in luminal A breast cancers (Fig. 7e–g).

We next evaluated the in vivo effects of HIFAL overexpression on tumorigenesis using xenograft mouse models. MCF-7 cells with enforced expression of wild-type, mutant HIFAL (loss of PKM2 binding motif) or HIFAL antisense were implanted subcutaneously into the fat pads of nude mice. The tumors with wild-type HIFAL grew dramatically faster than those of the other groups (Supplementary Fig. 7f–h). To further evaluate the therapeutic potential of targeting HIFAL in vivo, we implanted MDA-MB-231 cells in nude mice to establish breast tumor xenografts. When tumor volume reached 100 mm^3^, mice were treated with intraperitoneal injection of HIFAL LNAs. Knockdown of HIFAL significantly suppressed the tumor growth in vivo, measured by tumor growth curve and tumor weights (Supplementary Fig. 7i–k), as compared with the controls.

As was indicated in the above data, the dynamics of HIF-1α protein level did not coincide with the expression of HIF-1 target genes (Fig. 1a), suggesting that HIF-1 effects become less dependent on HIF-1α level during prolonged hypoxia, which may limit the therapeutic effect of HIF-1α inhibitor as a single agent in cancer treatment. Also, our above data showed that targeting HIFAL abrogated the transcription of HIF-1 target genes upon prolonged hypoxic treatment (Fig. 1g–j), we sought to test whether simultaneous inhibition of HIF-1α and HIFAL would exert synergistic effects to prevent tumor growth and thus employed an LNA-based anti-sense oligonucleotide^11^ to inhibit the expression of HIF-1α mRNA as reported in previous phase I clinical trial. Surprisingly, combined targeting HIF-α mRNA and HIFAL lncRNA almost greatly abolished breast tumor growth in xenograft model (Fig. 7h, i and Supplementary Fig. 7l–n) and was much more effective than targeting HIF-1α mRNA or HIFAL lncRNA alone. Immunohistochemistry revealed that PKM2/PHD3 mostly remains in cytoplasm upon treatment with the HIFAL targeting LNA (Fig. 7j and Supplementary Fig. 7o). Furthermore, the protein levels of HIF-1 target genes were suppressed upon treatment with HIF-1α LNA or HIFAL LNA alone as compared with the control group, but was nearly completely abolished in the combined treatment group (Fig. 7k). Consistently, HIFAL LNA or HIF-1α LNA reduced tumor cell proliferation, denoted by Ki67 staining, in the xenografts as compared with the control animals, while combined treatment with both LNAs further suppressed the cancer cell proliferation (Fig. 7l). Collectively, these data suggest that targeting HIFAL lncRNA in combination with HIF-1α inhibition emerges as a promising strategy to inhibit cancer growth.

## Discussion

HIF-1α is a master regulator in hypoxia-induced transcription and metabolic switch. Under normoxic conditions, HIF-1α is hydroxylated at proline (Pro) by PHD2 and binds to the von Hippel-Lindau (VHL), a tumor suppressor protein, leading to its own proteasomal degradation^40^. In contrast, the prolyl hydroxylation of HIF-1α is inhibited under hypoxia, thus HIF-1α protein level accumulates and peaks serval hours after oxygen is exhausted^41^. We and others discovered that the level of HIF-1α gradually decreased to almost the basal level after it reaches its peak although the cells are still in hypoxia^24–26^. However, our study went one step forward by revealing that once triggered by HIF-1α, the transcription of HIF-1α target genes continues to elevate even though the HIF-1α level declines, suggesting that the continuation of transactivation becomes less dependent on HIF-1α. HIF-1α transactivation is coordinated with a series of coactivators, such as CBP/p300 and PKM2. It has been shown that CBP/p300 is required to modify chromatin conformation by acetylating the histone and facilitating binding of the core transcriptional complex to target gene promoters^38,42^. On the other hand, PKM2, acts as key a glycolytic enzyme to drive the Warburg effect^43^. Under hypoxia, PHD3 binds to PKM2 to hydroxylase it at Pro-403/408^6^, which then recruits CBP/p300 to the promoters of HIF-1α target genes to facilitate HIF-1α binding by inducing histone H3 acetylation. These studies supported PKM2 as a key regulator of glycolytic metabolism and cancer progression. In addition, PKM2 and PHD3 are HIF-1α target genes^6,44^, suggesting that a positive feedback loop containing PKM2 and PHD3 is involved in assembling the HIF-1α transactivation complex. Therefore, we conclude that the transactivation complex becomes highly efficient to drive the transcription once fully assembled, as only basal level of HIF-1α protein is needed during prolonged hypoxia. However, the role of PKM2 in glycolytic metabolism and cancer progression remains controversial. Several reports have shown that PKM2 acts as a transcriptional co-activator in HIF-1 and β-catenin related transcription^6,45^. In the present study, we revealed that HIFAL serves as a scaffold to recruit PHD3 to PKM2 and induces the nuclear translocation of PKM2/PHD3 complex to activate HIF-1 transcription in human breast cancer. Together, these findings highlighted that the HIFAL was essential for PKM2 to drive HIF-1α mediated transcription in cancer development.

More importantly, our present study has presented the evidence for the central role of a lncRNA in the positive feedback loop of assembling the HIF-1α transactivation complex. LncRNAs have been shown to participate in multiple cell signaling transduction by functioning not only as guides, decoys or scaffolds to modulate protein-DNA or protein-protein interactions^46^, but also as enhancers to affect gene transcription from the enhancer regions (enhancer RNA) or their neighboring locus (noncoding RNA activator)^47^. Our present data revealed that upon abolishing HIFAL expression in the hypoxic cells, the transcription of HIF-1α target genes fail to keep increasing upon prolonged hypoxia and becomes dependent on the level of HIF-1α protein. Furthermore, our mechanistic study demonstrated that HIFAL acts as the scaffold to link PHD3 to PKM2, facilitating PHD3 to catalyze the hydroxylation of PKM2. Additionally, HIFAL harbors a motif within nt 240–300 and can binds to hnRNPs, including hnRNPF and hnRNPK, which assists the lncRNA and its bound complex of PHD3/PKM2 to translocate into the nucleus. It has been shown that the hnRNPs family contains a large number of RNA-binding proteins (RBPs) that contribute to nucleic acid metabolism including mRNA stabilization, alternative splicing, and translational and transcriptional regulations^48^. In agreement with our present findings, a recent study showed that certain lncRNAs harbor short fragments, named SIRLOIN, which allows them to bind to hnRNPK and therefore introduce them into nucleus^36^. Together with our findings, these observation supports that lncRNAs can act as signal transducers beyond the reported manners, such as guides, decoys or scaffolds, by inducing signal regulators into nucleus. Our findings suggested that the nuclear transportation of HIFAL relies on interaction with hnRNPF. Upon hypoxic conditions, hnRNPF overexpression is induced and undergoes nuclear relocation, thereby introducing the PKM2/PHD3 complex into the nucleus and probably guiding the complex to bind to the promotor of HIF-1α targeting genes. Eliminating the expression of HIFAL not only disassembles the PKM2/PHD3 complex, but also abolishes the nuclear transportation of PKM2 and PHD3. Furthermore, more than 90% of the HIFAL target genes overlapped with the HIF-1α target genes, as determined by CHIP-seq assays. Thus, HIFAL was essential for the assembly of the HIF-1 transactivation complex and acts as a key component in the positive feedback loop of HIF-1 transactivation (Fig. 7c) by recruiting and directing PKM2/PHD3 complex into nucleus.

Interestingly, we found that HIFAL expression is also driven by the HIF-1α transcription complex. Moreover, HIFAL is involved in its own transcription, therefore constituting a feed-forward loop driving HIFAL transcription and further enhancing the positive feedback loop of HIF-1 transactivation. Silencing HIFAL leads to 90% reduction in HIF-1 binding to its target genes, and thus dramatically decreases the efficiency of HIF-1 transactivation and suppresses glycolysis in the tumor cells under hypoxia. In contrast, our previous study showed that NKILA lncRNA forms a negative feedback loop with NFκB signaling, suggesting that lncRNAs may regulate their own expression by forming feedback loops with transcription factor circuits^21^. Furthermore, in analyzing the clinical significance of HIFAL in breast cancer progression, we found that high HIFAL level was associated with aggressive cancer phenotypes in both luminal and triple-negative breast cancers. However, the high HIFAL expression only correlated with poor outcome in the luminal B and triple-negative subgroups, but not in the luminal A subgroup. This result is consistent with the observation that the advanced breast cancers, as well as subgroups of triple-negative and luminal B breast cancers, are more dependent on glycolysis, whereas luminal A breast cancers are less dependent on glycolysis.

It has been well known that increased PKM2 and constitutive activation of HIF-1 commonly occurs in human tumor as a result of consistent hypoxia in cancer microenvironment^49,50^, leading to numerous target gene expression to switch from oxidative to glycolytic metabolism^5,9^. Activation of the hypoxia-inducible factor (HIF-1) has been linked to pro-tumorigenic responses, tumor angiogenesis, metastasis, and drug resistance in cancer development^51^. Although no HIF-1 specific inhibitor is clinically available to date, targeting HIF-1 related transcription is considered as a promising strategy for cancer treatment. Recently, an LNA-based anti-sense oligonucleotide which specifically binds and inhibits the expression of HIF-1α mRNA has shown limited anti-cancer effect in phase I trial^12^. Herein, we applied the same HIF-1α targeting LNA as well as the HIFAL targeting LNA in animal experiments. Either of the LNAs showed mild therapeutic effect in breast cancer xenografts, suggesting that using single drug to target the HIF-1 transactivation may have only limited anti-cancer effect. Notably, applying HIF-1α LNA to treat the tumor derived from HIFAL null cancer cells significantly suppresses tumor growth in mice (Supplementary Fig. 7l–m). In line with these results, the combination of HIF-1α LNA with the LNA targeting HIFAL significantly abolishes xenografted tumor growth.

Together, our results revealed that HIF-1 related transactivation is not only dependent on HIF-1α, but also on the positive feedback loop composed of the HFILA/PKM2/PHD3 complex. HIFAL transcription is triggered by the HIF-1 related axis, which includes the HIFAL/PKM2/PHD3 complex and demonstrates a feed-forward loop enforcing HIFAL expression and to further enhance HIF-1 transactivation, suggesting a central role of HIFAL in driving HIF-1 mediated glycolysis. Therefore, our data indicate that combined treatment of targeting both HILFA and HIF-1 is a promising strategy to treat cancer patients.

## Methods

### Cell lines

Breast cancer cell lines (Table 1) were obtained from American Type Culture Collection (ATCC) and cultured in DMEM medium supplemented with 10% fetal bovine serum (Gibco) in a 5% CO_2_ incubator at 37 °C. For hypoxic culturing, 0.6% O_2_ was used.

**Table 1:** Key resources table.

REAGENT or RESOURCE	SOURCE	IDENTIFER
Antibody		
Mouse HIF-1α	BD	610959
Rabbit PKM2	CellSignaling Technology	4053 S
Rabbit PHD3	Abcam	ab30782
Mouse hnRNPK	Santa cruz	Sc53620
Rabbit hnRNPF	Abcam	Ab50982
Mouse JMJD5	Abcam	Ab10639
Rabbit Hydroxyproline	Abcam	Ab37067
Rabbit LaminB1	Cell Signaling Technology	12255 S
Mouse MnSOD	BD	611580
Rabbit HIF-2*α*	Cell Signaling Technology	59973 S
Rabbit H3K27me3	Cell Signaling Technology	9733 S
Flag	Abcam	Ab205606
Mouse β-actin	abcam	Ab6276
Rabbit PKM1	proteintech	15821-1-AP
Biotin-antibody	Invitrogen	033700
Alexa Fluor Goat anti-mouse 594	Invitrogen	A11032
Alexa Fluor Goat anti-mouse 555	Invitrogen	A28180
Digoxin-antibody	Abcam	Ab419
Rabbit LDHA	Cell Signaling Technology	3582
Rabbit Ki67	Abcam	Ab15580
Rabbit GLUT1	Abcam	Ab115730
Bacterial and Virus Strains		
One Shot Stbl3	Invitrogen	C7373–03
pLentiCRISPR v2 plasmid	^57^	18011CZ
pGL3-luciferase reporter vector	Jeneray biotech	VQP0124
PcDNA3.1 vector	Jeneray biotech	Addgene#69352
DH5α competent cells	TAKARA	9057
Chemicals, Peptides, and Recombinant Proteins		
Puromycin	Gibco	A11138–03
10X T4 Ligation Buffer	New England Biolabs	#B0202
Pierce™ IP Lysis Buffer	Thermo Fisher Scientific	87787
Trizol	Invitrogen	15596–026
Dynabeads M-280 Streptavidin magnetic beads	Invitrogen	11205D
recombinant myc-labeled PHD3	Origene	TP310319
His-labeled PKM2	Origene	TP721212
EZ-Magna ChIP A/G Recombinant protein	Merck Millipore	17–10086
NEBNext Ultra II Q5 Master Mix	New England Biolabs	M0544L
Colorimetric peptide assay	Invitrogen	23275
16%Formaldehyde (w/v)	Thermo Fisher Scientific	28908
Pierce chip-grade protein A/G magenetics beads	Invitrogen	26162
Deoxynucleotide (dNTP) Solution Mix	New England Biolabs	N0447L
10 MM Bio-16-UTP 1 TUBE EACH	Invitrogen	AM8452
Recombinant RNasin® Ribonuclease Inhibitor	Promega	N2515
Halt™ Protease Inhibitor Cocktail (100X)	Pierce	87786
Critical Commercial Assays		
Superscript First-Strand cDNA Synthesis Kit	Invitrogen	18080–051
SYBR Premix Ex Taq II kit	TAKARA	DRR081A
DIG Luminescent Detection Kit for Nucleic Acids	Roche	11363514910
Cell fractionation assay PARIS^TM^ kit	Ambion	AM 1921
SMARTer RACE 5’/3’ Kit	Clontech	634860
Magna RNA Immunoprecipitation Kit	Millipore	17–700
TranscriptAid T7 High Yield Transcription Kit	Ambion	AM1333
MagnaChIP HiSens Chromatin IP Kit	Merck Millipore	17–10461
Lightshift^TM^Chemiluminescent RNA EMSA kit	Thermo Fisher Scientific	20158
Glucose Uptake Colorimetric Assay Kit	Biovision	k676
lactate Colorimetric/Fluorometric Assay kit	Biovision	k607
XFglycolytic stress test kit	Agilent Technologies	103020100
Experimental Models: Cell Lines		
Human: MDA-MB-231	N/A	N/A
Human:MCF-7	N/A	N/A
Human:76 N	N/A	N/A
Human:293 T	N/A	N/A
Human: T47D	N/A	N/A
Human:SK-BR3	N/A	N/A
Human:BT549	N/A	N/A
Human:MDA-MB-468	N/A	N/A
Experimental Models		
Balb/c nu/nu mouse (3–4 weeks old female)	Vitonlihua Laboratory Animal Center	11400700211483
Oligonucleotides		
GampeRHIFAL LNA1	Exiqon	CATTCTGGGACGGAGA
GampeRHIFAL LNA2	Exiqon	GTAACATGGTGATAAT
GampeR HIF1α-1 LNA	Exiqon	TGGCAAGCATCCTGTA
GampeR Control LNA	Exiqon	AACACGTCTATACGC
LNA mRNA Detection Probe	Exiqon	ACGGAGTAATGTTGGGTAAGCT
beta-actin, hsa/mmu/rno, 5-dig labeled	Exiqon	E61201
HIFAL and hnRNPF sgRNA (See Table S1for sequences)	Invitrogen	N/A
Sequences of siRNAs (See Table S1 for sequences)	Invitrogen	N/A
Primers for qRT-PCR (See Table S1 for sequences)	Invitrogen	N/A
Primers for ChIP assay (See Table S1 for sequences)	Invitrogen	N/A
Primers for 5’ and 3’RACE (See Table S1 for sequences)	Invitrogen	N/A
Deposited Data		
ChIP-seq reads	http://www.bioinformatics.babraham.ac.uk/projects/fastqc/	^55^
adapter sequences	http://hannonlab.cshl.edu/fastx_toolkit/index.html	^56^
Software and Algorithms		
SPSS version 16.0	Chicago, IL	N/A
LaserConfocal Scan soft Zen2012	Carl Zeiss	N/A
TCGAbiolinks	http://bioconductor.org/packages/release/bioc/html/TCGAbiolinks.html	^61^
All spectra of modified peptides	http://prospector.ucsf.edu/prospector/cgi-bin/msform.cgi?form=msproduct	^62^

### Mice

In total 5–6 weeks female Balb/c-nu/nu mice were purchased from Beijing vitonlihua Laboratory Animal center and housed under standard conditions of the room temperature range between 20 and 26 °C, the relative environmental humidity of 50–70%, the semi-natural light cycle of 12:12 or 10:14 h light: dark. All animal studies were carried out according to the Institutional Animal Care and Use Committee at the Medical School of Sun Yat-Sen University and laboratory animal facility has been accredited by AAALAC (Association for Assessment and Accreditation of Laboratory Animal Care International) and the IACUC (Institutional Animal Care and Use Committee) of Guangdong Laboratory Animal. Monitoring Institute approved all animal protocols used in this study.

### Patients and sample collection

Paraffin-embedded and fresh samples of paired para-tumor and breast cancers tissue were obtained from the breast tumor center, Sun Yat-Sen Memorial Hospital, Sun Yat-Sen University, without any treatment before surgery. All samples were collected with signed informed consent from patients and this study was approved by the institutional review board (IRB) of Sun Yat-Sen Memorial Hospital, Sun Yat-Sen University.

### Immunoblotting

The following primary antibodies (Table 1) were used in immunoblotting: HIF-1α (1:1000, 610959, BD), PKM2 (1:1000, 4053 S, CST), PHD3 (1:1000, ab30782, Abcam), hnRNPK (1:1000, sc53620, santa cruz), hnRNPF (1:500, ab50982, abcam), JMJD5 (1:1000, ab10639, abcam), hydroxyproline (1:500, ab37067, abcam), β-actin (1:1000, ab6276,abcam), laminB (1:1000,12255 S,CST), MnSOD (1:1000,611580,BD), HIF-2α(1:1000,59973 S.CST), PKM1(1:1000,15821-1-AP, proteintech).

### Microarray

Total RNA was extracted using Trizol reagent. CapitalBio Technology Human LncRNA Array v4 was used to analyze the expression profile microarray of lncRNA in the light of the manufacturer’s protocol. After hybridization, the Agilent Microarray Scanner scanned the processed pictures. The raw results of the expression profiling microarray were analyzed and further standardized quantile and displayed as log2 transformation by the GeneSpring software. MeV4 .7 was used to generate the heatmap according to the intensity.

### LNAs, siRNAs, and constructs

For knockdown experiments, cells were transiently transfected with LNAs antisense oligonucleotides (Exiqon) using Lipofectamine 2000 (Invitrogen, Carlsbad, CA) in the light of the manufacturer’s protocol. The LNAs antisense oligonucleotides and siRNA sequences are listed in Supplementary Table 1.

For overexpression experiments, a biotin-labeled HIFAL lncRNA or an antisense control was directly introduced into cells by lipofectamine transfection. Briefly, a biotin-labeled HIFAL lncRNA or an antisense control in the opti-MEM, was been added into the diluted lipofectamin3000 and incubated for 20 min. Then RNA-lipid complex were added in cells^35^. Antisense control, wildtype or mutant of HIFAL was also cloned into pcDNA3.1 or MSCV vector for overexpression. ΔhA (deleting hairpin A, nt 4–19) or ΔhB (deleting hairpin B, nt 30–57), ΔhC (deleting hairpin C, nt 506–532) sequences are listed in Supplementary Table 1.

For the binding of HIFAL and hnRNP F experiments, wildtype or mutant of hnRNP F was cloned into pcDNA3.1 vector. The mutated sequences of hnRNP F are listed in Supplementary Table 1.

### Quantitative RT-PCR and Northern blot

Total RNA was extracted from fresh tissues and cultured cells using Trizol (15596–026, Invitrogen, Carlsbad, CA) in accordance with the manufacturer’s protocol. Superscript First-Strand cDNA Synthesis Kit (18080–051, Invitrogen, Carlsbad, CA) was used to reverse transcribe 500 ng total RNA into cDNAs. Quantitative RT-PCR was performed using SYBR Premix Ex Taq II kit (DRR081A, TAKARA,Otsu, Shiga, Japan) on LightCycler 480 System (Roche, Basel, Switzerland). Northern blot assays were performed using DIG Luminescent Detection Kit for Nucleic Acids (11363514910, Roche, Basel, Switzerland) in the light of the manufacturer’s instructions. The primer sequences were listed in Supplementary Table 1.

### HIFAL expression in cytoplasm and nucleus

Cell fractionation assay in accordance with PARIS^TM^ kit (Cat number: AM 1921) as followed: First, Cells washed twice with ice-cold PBS were resuspended with 300 μl cold Cell Fractionation Buffer (10 U/ml RNase inhibitor) on ice for five minutes and then were centrifuged at 500 *g* in 4 °C centrifuge for 5 min. Then the supernatant of the extract was shifted into a new microcentrifuge tube and centrifuged again at 500 *g* centrifuge for one minute in 4 °C. The cytoplasmic fraction (the supernatant) was shifted again into a new tube. Then, the nuclear fraction was washed once in cold cell fractionation buffer and resuspended and centrifuged 500 × *g* at 4 °C for one minute. Remove and discard the supernatant. The nuclear extract was lysed with 300 μl cell disruption buffer and RNA was extracted from the nuclear pellet in accordance with the manufacturer’s protocol.

For assessing the nuclear and cytoplasm HIFAL abundance, we added the synthetic cel-mir-39 (at a final concentration of 25fmol)^52^ as exogenous internal reference in the nuclear and cytoplasm lysate which was mixed with the equal volume of 2X Lysis/Binding Solution. QRT-PCR was performed using Mir-x^TM^ miRNA First-Strand Synthesis and TB Green^TM^ qRT-PCR(Takara).

### Rapid Amplification of Cloned cDNA Ends (RACE)

RACE was conducted using SMARTer RACE 5’/3’ Kit Components (634860, Clontech) in the light of manufacturer’s instructions. Primers used in RACE are listed in Supplementary Table 1.

### RNA immunoprecipitation and RNA pulldown

For RNA immunoprecipitation, lysates of MDA-MB-231 cells after hypoxia for 24hs were immunoprecipitated using anti-PKM2 and anti-PHD3 primary antibody. RNA immunoprecipitation was performed using Magna RNA immunoprecipitation RNA-Binding Protein Immunoprecipitation Kit (17–700, Millipore, Billerica, MA) following the manufacturer’s protocol.

For RNA pull-down, transcriptAid T7 High Yield Transcription Kit (Invitrogen, USA) was used to transcribe the biotin-labeled RNAs. Bio-16-UTP was added in the in vitro transcription. Briefly, 5 pmol of bio-labeled RNA was heated in RNA folded structure buffer (0.1 M KCl, pH 7, 10 mM MgCl_2_, 10 mM Tris) for two minutes at 95 °C, then on ice for three minutes and then put for thirty minutes at room temperature. Folded RNA (5 μg) was then blended with cell lysates (5 mg) in 500ul Pierce™ IP Lysis Buffer (87787, Thermo Fisher Scientific) and incubated for 1 hr at room temperature. Dynabeads M-280 Streptavidin magnetic beads (50 μl, invitrogen, USA) were added into the binding reaction sample and further suspended for one hour. Washed beads were boiled in 1X protein loading buffer. The retrieved proteins were separated and analyzed by Western blot.

Mass spectrum followed with RNA pull down was performed to identify the proteins interacting with HIFAL. Three micrograms of bio-labeled RNA were heated for two minutes at 95 °C, then on ice for three minutes, provided with RNA folded structure buffer (0.1 M KCl, pH 7, 10 mM MgCl_2_, 10 mM Tris) and transferred to at RT for thirty minutes in order to form normal secondary structure. 1 mg of cell lysis in RIP buffer were mixed with folded RNA and suspended at room temperature for 1 h. Then 60 microliters Streptavidin agarose beads (Invitrogen) were added into the interacting reaction sample and mixed for 1 h at room temperature. The beads were washed rotationally five times by Handee spin columns (Thermo), and then boilded in protein loading buffer. The pull-down protein was analyzed by western blot^53^. Silver staining was conducted in terms of the manufacturer’s protocols with silver staining kit (LC6100, Thermo Fisher Scientific).

### Immunoprecipitation

Cells were completely lysed in IP buffer containing protease inhibitors, 25 mM Tris HCl pH 7.4, 1% NP-40, 5% glycerol, 1 mM EDTA, and 150 mM NaCl. The lysates were incubated on ice for five minutes with periodic mixture, then transferred into new microcentrifuge tubes and centrifuged for ten minutes at 12,000 g. The extracted supernatants were shifted into new microcentrifuge tubes in order to measure the protein concentration using the BCA method. The protein was divided equally to perform the immunoprecipitation. Antibody against PHD3 (1:100) or PKM2(1:100) was added into the cell lysates for immunoprecipitation overnight at 4 °C, and then incubated with rabbit or mouse IgG antibody (1:100) as control for 1 h. The Dynabeads Protein A (10002D, Invitrogen) was added and further incubated at room temperature for one hour. The immunocomplexes were washed five times by IP lysis buffer and boiled in 1 × loading buffer for western blot.

### Chromatin immunoprecipitation (ChIP)

ChIP was performed with MagnaChIP HiSens Chromatin IP Kit (17–10461, Merck Millipore) following the manufacturer’s protocol. In brief, cells were cultured under hypoxia for 32 h and then cross-linked with formaldehyde at 37 °C for 10 min, quenched with glycine, and then sonicated to generate 300–600 bp DNA fragments using an Ultrasonic Cell Disruptor (Diagenode, Liège, Belgium). The antibodies against HIF1α (610959, BD), PKM2 (4053 S, CST), biotin-ab (033700, Invitrogen), p300 (ab54984, Abcam), H3K9ac (ab4441, Abcam), were used to for immunoprecipitation. The binding of the HIFAL promoter to HIF1α, PKM2, biotin-ab, or IgG was quantified using quantitative PCR with primers. Chip primers sequences were listed in Supplementary Table 1.

### Biotin-HIFAL Chromatin immunoprecipitation

TranscriptAid T7 High Yield Transcription Kit (Invitrogen, USA) was used to transcribe biotin-labeled RNAs. Bio-16-UTP was added in the in vitro transcription. In vitro transcriptional RNA concentration was measured by nanodrop 2000. MDA-MB-231 cells (10 cm plate) were transfected with the folded RNA (15 μg) for 8 h and were cultured under hypoxia for 32 h. ChIP was performed in light of the manufacturer’s instructions.

### ChIP-seq library preparation and Illumina sequencing

Illumina sequencing libraries were generated using five nanograms of input DNA or ChIP-enriched DNA according to a modified version of the Illumina ChIP-seq instruction. Briefly, the DNA End-Repair Kit was used to end-repair DNA fragments.

Klenow fragment (New England Biolabs) was used to add a single “A” base. Then the fragments were ligated to Illumina Indexed adaptors (NEBNext® Multiplex Oligos for Illumina kit) using T4 DNA ligase (New England Biolabs). The magnetic bead was used to enrich the ligated products and remove the unligated adaptors. The enriched ligated products were then subjected to 16-cycle PCR (NEBNext® Multiplex Oligos). PCR product was purified by magnetic bead. The library was quantified by PCR using Qubit fluorometer (Invitrogen). Two barcoded libraries were mixed and sequenced to 150 bp in a single lane following standard procedures for cluster amplification and sequencing by synthesis on an Illumina HiSeq2000.

### ChIP-seq data analysis and visualization

FASTQCv.11.8 was used to assess the quality of raw 150-nucleotide ChIP-seq reads (97% bases ≥Q30). the FASTX toolkit filtered adapter sequences out (~ 13%). Bowtie 0.12.7 with zero-mismatches was used to compare reads with the reference human genome (GRCh37/hg19) and non-unique comparations were discarded^54^. HIFAL CHIP-seq peak calling was a computational method used to identify areas in the genome that have been enriched with aligned reads. For HIFAL ChIP-seq experiments, MBA-MD-231 cells which are transfected with biotin-HIFAL were divided in input and anti-biotin pull-down group. The biotin-binding site are compared against the input group to determine if the site of enrichment was likely to be HIFAL CHIP-seq peaks. Enriched HIF-1α and biotin ChIP peak regions were determined using MACS2v2.1.1.3 with both ChIP and control (input) samples^55^. Motif enrichment was performed using HOMERv4.1.1^56^. Overlapped unique peaks were identified by using BED toolsv2.29.0. Heatmaps were generated using deep tools, compute matrix and plot heatmap functions with the following parameters: “compute matrix reference-point -S *big Wig -b 500 -a 500 –missing DataAsZero” and “plot heatmap–kmeans 1–zMin 0–zMax 15”. Metagenes were established by acquiring the mean value of reads per 150 bp bin covering all regions as displayed.

### Luciferase reporter assay

To detect the transcript activity of HIFAL by HIF-1α, we cloned the HIFAL promoter sequence between -1 to -2000 into pGL3-enhancer vector (Millipore). HEK293 cells were transiently transfected with pRL-TK-renilla-luciferase plasmid and the wildtype or the mutated promoter constructs. After 48 h, cells were harvested and luciferase activity was measured by Dual-Luciferase Reporter Assay System in accordance with the manufacturer’s instructions (Promega, WI, USA). The division of firefly luciferase activity with renilla luciferase of the same sample was calculated to get the transfection efficiency in order to normalize the data. The mutation was generated as following:

wt: …TGCGCCCGAG**CACGT**ACTGAGG**CGTG**GCCTGCCGCGCGCCGG…

mutHRE: …TGCGCCCGAG**AAAAA**ACTGAGG**CGTG**GCCTGCCGCGCGCCGG…

mutHRE: …TGCGCCCGAG**CACGT**ACTGAGG**AAAA**GCCTGCCGCGCGCCGG…

mutHRE: …TGCGCCCGAG**AAAAA**ACTGAGG**AAAA**GCCTGCCGCGCGCCGG…

### Plasmids encoding Cas9 and sgRNA

To obtain clustered regularly interspaced short palindromic repeats (CRISPR)/CRISPR-associated protein-9 (Cas9) mediated knockout of HIFAL, we designed 4 guide RNAs (gRNAs) targeting the first exon of HIFAL gene segments, which were cloned into pLentiCRISPR v2 plasmid^57^. CRISPR/Cas9 carriers were co-transfected into HEK 293FT cells to generate lentiviral particles, with envelope and packaging plasmids psPAX2 and pMD2.G.

### In vitro binding assay

Briefly, purified recombinant myc-labeled PHD3 (TP310319, Origene), His-labeled PKM2 (TP721212, Origene), and folded HIFAL were pooled in IP lysis buffer and were incubated for one hour at room temperature. The immunocomplexes were analyzed by immunoprecipitation and immunobloting.

### In situ hybridization (ISH) and fluorescent in situ hybridization (FISH)

The probe targeting HIFAL was designed by Exiqon and was listed in Supplementary Table 1. For ISH, the samples were dewaxed and rehydrated, and then digested for 5–15 min with 0.05% trysin at 37 °C, then fixed for 5 min in 4% paraformaldehyde, and hybridized with the 5′digoxin-labeled HIFAL probe (Exiqon, Vedbaek, Danmark) at 55 °C overnight, then subsequently incubated with anti-digoxingenin antibody (Abcam, ab419) overnight at 4 °C. The staining scores were calculated on the basis of both the proportion and intensity of positive cells in 10 random fields under a 20 × objective^21^. The cells at different staining intensity were scored on a grade of 0 (no staining), 1 (light blue), 2 (blue), and 3 (dark blue). The percentage of positive staining of tumor cells on the slides was scored as follows: 0, no positive cells; 1, <20%; 2, 10–50%; and 3, >50%. The staining index (SI) was determined as follows: SI = percentage of positively stained cells × staining intensity. The expression was assessed using SI and recorded as 0, 1, 2, 3, 4, 6, or 9 according to this method. On the basis of the distribution of SI score frequency for HIFAL expression level and the evaluation of heterogeneity with Kaplan–Meier statistical analysis on overall survival, a cut-off value was regarded as SI score 3, which were defined as low (SI < 3) or high (SI > 3) for HIFAL expression.

To perform FISH assay, cells were gently rinsed in RNAase-free PBS and then fixed in 4% RNAase-free formaldehyde solution (pH 7.4) at room temperature (RT) for 40 min. Then the fixed cells were digested for 3 min at RT with 0.05% trypsin and permeabilized in 0.1% Triton X-100 solution with RNAase inhibitor on ice for 10 min; washed 3 times for 10 min each time with RNAase-free PBS and fixed again in 4% RNAase-free formaldehyde for 5 min. Subsequently, the fixed and permeabilized cells were prehybridized for two hours at 52 °C with the hybridization solution. At the same time, the anti-HIFAL oligodeoxynucleotide probe which had been conjugated with DIG (Exiqon) was diluted 1:1000 to a final concentration 25 nM by the hybridization solution. Then the cells were hybridized with the diluted probe for 16 h at 52 °C in the wet chamber. The hybridized cells were washed for 5 min in 2 × SSC at 52 °C, then for 25 min in 50% deionized formamide which was diluted in 4 × SSC at 52 °C. The cells were incubated with the antibody against DIG (Roche) which had been conjugated with fluorescence to perform confocal microscopy for overnight at 4 °C. Hochest33342 was used for nucleus staining, images were acquired by laser confocal microscopy (Zeiss).

### Immunofluorescence and immunohistochemistry

Immunofluorescence staining in cultured cells was performed as follows. Cells were gently rinsed twice with cold PBS and then fixed for 40 min in 4% formaldehyde solution (pH 7.4) at RT. Then the fixed cells were permeabilized in 0.1% Triton X-100 solution on ice for 10 min, and incubated with the primary antibodies against PKM2 (4053 S, CST), PHD3 (ab30782, Abcam), hnRNPK (sc53620, Santa Cruz), hnRNPF (ab50982, Abcam) overnight at 4 °C, followed by staining with the secondary antibody which had been linked to Alexa Fluor 594 (A-11032, Invitrogen)^58^. Cells were then stained with hochest33342. Images were obtained using laser confocal microscopy (Leica Microsystems).

Immunohistochemistry was performed as follows: After dewaxing and rehydration, Endogenous peroxidase was eliminated by hydrogen peroxide (3%). The slides were then incubated at 4 °C overnight with primary antibodies against HIF-1α (610959, BD), PKM2 (4053 S, CST), PHD3 (ab30782, Abcam), GLUT1 (ab115730, Abcam), Ki67 (ab15580, Abcam) and LDHA (3582, CST)^59^. The signal was detected by the EnVision/HRP Kit (Dako, Carpinteria, CA). The immunostaining was detected by an Olympus BX51 microscope (Olympus, Tokyo, Japan). The staining scores were calculated according to the proportion of the staining-positive area in 10 random fields under a 40 × objective. The percentage of positive staining of tumor cells on the slides was scored as follows: 0, no positive stained area; 1, <25%; 2, 25%–50%; and 3, >50%. The exhibited staining score was the total number of the 10 random fields.

### Glucose consumption and lactate production assays

When the cultured cells grew to ~40% confluence, the fresh culture medium was added. After 24 hr, the culture medium was harvested for glucose consumption and lactate production with the kits from biovision (ab136955, ab65331). Cell counting was carried out by cell counter (Beckman Coulter).

### In vitro hydroxylation assay

Preincubation of His-labeled PKM2 (TP721212, Origene) and myc-labeled PHD3 (TP310319, Origene) was for 30 min at room temperature. Then the reaction buffer was incubated for 1 h at 30 °C with the folded HIFAL and 100 mMFeCl2, 5 mMascorbate, and 1 mM a-ketoglutarate.

### Seahorse assay

An XF24 Extracellular Flux Analyzer (Seahorse Bioscience, North Billerica, MA, USA) was applied to detect the effects of the inhibitors on MDA-MB-231 cells. A total of 20,000 cells/well which had been transfected with LNA-NC, HIFAL LNA-1, and HIFAL LNA-2 were seeded into the Seahorse XF24 culturing plates within medium overnight. Cells were gently washed once in PBS and then cultured for one hour at 37 °C in Seahorse incubation medium containing 1 μM glucose and 2 mM L-glutamine. To ensure accurate detection of extracellular pH, cells were cultured in a CO2-free incubator. The detection of extracellular acidification rate (ECAR) were performed at baseline and following sequential injections of glucose (10 mM), oligomycin (1.0 μM), 2-DG (50 mM). Glucose fuels glycolysis. ECAR is presented as the mean ± SD of experimental triplicates.

### Chromatin-binding protein enrichment

A total of 10 cm dish cells were harvested and washed, then transferred to 1.5 ml EP tubes quickly and added buffer A (1.5 mM MgCl2, 10 mM KCl, 10% glycerol, 0.34 M sucrose, 1 mM DTT, 0.1% triton X-100). Supernatant (none chromatin-binding proteins) from pellet (nuclei) were separated and pellet were resuspended in Buffer B **(**0.2 mM EDTA, 1 mM DTT, 3 mM EDTA), washed, sonicated, and centrifuged, and chromatin-binding protein were harvested^60^.

### Animal experiments

A total of 2 × 10^6^ of MDA-MB-231 cells after hypoxia for 24 h were injected orthotopically into the mammary fat pad of mice in 100 μl sterile PBS. When tumors reached 200 mm^3^, 10 mg/kg of LNA-1 or LNA-NC were injected intraperitoneally every three days for three weeks. According to the following formula V (mm^3^) = 0.5 × (length × width^2^), xenograft volume was determined every three days. Tumor were collected, weighed, and snap-frozen when LNA treatments were ended.

In FDG-PET scan animal experiment, when tumors reached 200 mm^3^, 10 mg/kg of LNA-1, LNA-2, or LNA-NC were injected intraperitoneally every day for 3 days. Before PET-CT Scanning, mice were fasted for 8 h. Then, for FDG-PET Scanning, a total of 0.4 mCi per mouse of FDG was administered through the tail vein injection of the anesthetized mouse. After a “uptake” for 1 h, a static scan was performed using a NanoPET/CT scanner (Bioscan/Mediso). Three-dimensional image was acquired. The mice maintained their supine position during the procedure. Then a CT scan was performed before the whole-body FDG-PET image was picked up across the same range. Counts which were obtained per minute (cpm) were converted to becquerels (Bq), and values of which were normalized based on the injected dose and the volume of the interesting region. FDG-uptake rate was determined in the light of the following formula: (activity in tumor in Bq)/(injected activity in Bq)/(mouse weight in cm^3^) in order to adjust the injected and metabolic activity changes between inspections and to obtain tumor-specific uptake.

### Statistics and reproducibility

Graphpad Prism 7 and SPSS 16.0 statistical software package were used to perform all statistical analysis. *P* values in most of in vitro and animal experiments were determined by one-way ANOVA and unpaired two-sided Student’s t test. The difference between groups was determined by post hoc tests. The relationship between HIFAL expression levels and clinicopathological status was analyzed by Chi-square test. The Kaplan-Meier method and the log-rank test were used to plot and compare survival curves. Wald test was used in multivariate Cox proportional hazard analysis of HIFAL expression levels and disease-free survival (DFS). Data were presented as mean ± S.D. of independent experiments triplicates. In all experiments, *p < 0.05, **p < 0.01 and ***p < 0.001.

Results in Figs. 2a, d–e, f–j, l–n, p–q, 3a–c, e–I, 4b–c, e, g–i, k–n, 6g and 7j–l, supplementary Figs. 1b, m–n, p and 2a–b, d–e, g–h, j, n, q–r and 3b–c, e–h, j–k, n–o and 4a, c–I and 5a–b and 6i–j and 7o are representative data of three independent repeats. And there were similar results in three independent repeats.

### Reporting summary

Further information on research design is available in the Nature Research Reporting Summary linked to this article.

## Supplementary information

Supplementary Information

Reporting summary

## Data Availability

The expression profiling microarray data for breast cancer tissues and cells and CHIP-seq have been deposited in public database under the accession code GSE159490 and CRA003355. The microarray and CHIP-seq data referenced during the study are available in a public repository from the website (https://www.ncbi.nlm.nih.gov/geo/query/acc.cgi?acc=GSE159490 and https://bigd.big.ac.cn/gsa/s/Z9K8598l). All the other data supporting the findings of this study are available within the manuscript and its supplementary information files and from the corresponding author upon reasonable request. A reporting summary for this article is available as a Supplementary Information file. Source data are provided with this paper.

## Source data

Source data

## References

[1] Sutherland RM (1998). Tumor hypoxia and gene expression–implications for malignant progression and therapy. Acta Oncol..

[2] Sullivan R, Graham CH (2007). Hypoxia-driven selection of the metastatic phenotype. Cancer Metastasis Rev..

[3] Schofield CJ, Ratcliffe PJ (2004). Oxygen sensing by HIF hydroxylases. Nat. Rev. Mol. cell Biol..

[4] Maxwell PH, Pugh CW, Ratcliffe PJ (2001). The pVHL-hIF-1 system. A key mediator of oxygen homeostasis. Adv. Exp. Med. Biol..

[5] Wheaton WW, Chandel NS (2011). Hypoxia. 2. Hypoxia regulates cellular metabolism. Am. J. Physiol. Cell Physiol..

[6] Luo W (2011). Pyruvate kinase M2 is a PHD3-stimulated coactivator for hypoxia-inducible factor 1. Cell.

[7] Masoud GN, Li W (2015). HIF-1alpha pathway: role, regulation and intervention for cancer therapy. Acta Pharm. Sin. B.

[8] Mathupala SP, Rempel A, Pedersen PL (2001). Glucose catabolism in cancer cells: identification and characterization of a marked activation response of the type II hexokinase gene to hypoxic conditions. J. Biol. Chem..

[9] Seagroves TN (2001). Transcription factor HIF-1 is a necessary mediator of the pasteur effect in mammalian cells. Mol. Cell. Biol..

[10] Lee K (2009). Acriflavine inhibits HIF-1 dimerization, tumor growth, and vascularization. Proc. Natl Acad. Sci. USA.

[11] Greenberger LM (2008). A RNA antagonist of hypoxia-inducible factor-1alpha, EZN-2968, inhibits tumor cell growth. Mol. Cancer Ther..

[12] Jeong W (2014). Pilot trial of EZN-2968, an antisense oligonucleotide inhibitor of hypoxia-inducible factor-1 alpha (HIF-1alpha), in patients with refractory solid tumors. Cancer Chemother. Pharm..

[13] Chen W (2016). Targeting renal cell carcinoma with a HIF-2 antagonist. Nature.

[14] Scheuermann TH (2013). Allosteric inhibition of hypoxia inducible factor-2 with small molecules. Nat. Chem. Biol..

[15] Wang HJ (2014). JMJD5 regulates PKM2 nuclear translocation and reprograms HIF-1alpha-mediated glucose metabolism. Proc. Natl Acad. Sci. USA.

[16] Yang W (2012). ERK1/2-dependent phosphorylation and nuclear translocation of PKM2 promotes the Warburg effect. Nat. Cell Biol..

[17] Guttman M, Rinn JL (2012). Modular regulatory principles of large non-coding RNAs. Nature.

[18] Wang KC, Chang HY (2011). Molecular mechanisms of long noncoding RNAs. Mol. cell.

[19] Li W (2013). Functional roles of enhancer RNAs for oestrogen-dependent transcriptional activation. Nature.

[20] Xiang JF (2014). Human colorectal cancer-specific CCAT1-L lncRNA regulates long-range chromatin interactions at the MYC locus. Cell Res..

[21] Liu B (2015). A cytoplasmic NF-kappaB interacting long noncoding RNA blocks IkappaB phosphorylation and suppresses breast cancer metastasis. Cancer cell.

[22] Yang F, Zhang H, Mei Y, Wu M (2014). Reciprocal regulation of HIF-1alpha and lincRNA-p21 modulates the Warburg effect. Mol. cell.

[23] Chen F (2019). Extracellular vesicle-packaged HIF-1alpha-stabilizing lncRNA from tumour-associated macrophages regulates aerobic glycolysis of breast cancer cells. Nat. Cell Biol..

[24] Wang, K., Chen, Y., Ferguson, S. D. & Leach, R. E. MTA1 and MTA3 Regulate HIF1a Expression in Hypoxia-Treated Human Trophoblast Cell Line HTR8/Svneo. *Med J Obstet Gynecol* **1** (2013). PMC4332396 25705708

[25] Bonello S (2007). Reactive oxygen species activate the HIF-1alpha promoter via a functional NFkappaB site. Arterioscler Thromb. Vasc. Biol..

[26] Uchida T (2004). Prolonged hypoxia differentially regulates hypoxia-inducible factor (HIF)-1alpha and HIF-2alpha expression in lung epithelial cells: implication of natural antisense HIF-1alpha. J. Biol. Chem..

[27] Rossignol F, Vache C, Clottes E (2002). Natural antisense transcripts of hypoxia-inducible factor 1alpha are detected in different normal and tumour human tissues. Gene.

[28] Wu R (2018). Long non-coding RNA HIF1A-AS2 facilitates adipose-derived stem cells (ASCs) osteogenic differentiation through miR-665/IL6 axis via PI3K/Akt signaling pathway. Stem Cell Res Ther..

[29] Chen D (2017). Comparison of HIF1AAS1 and HIF1AAS2 in regulating HIF1alpha and the osteogenic differentiation of PDLCs under hypoxia. Int J. Mol. Med.

[30] Hu CJ, Wang LY, Chodosh LA, Keith B, Simon MC (2003). Differential roles of hypoxia-inducible factor 1alpha (HIF-1alpha) and HIF-2alpha in hypoxic gene regulation. Mol. Cell Biol..

[31] Tang Y, Zhou T, Yu X, Xue Z, Shen N (2017). The role of long non-coding RNAs in rheumatic diseases. Nat. Rev. Rheumatol..

[32] Cockman, M. E. et al. Lack of activity of recombinant HIF prolyl hydroxylases (PHDs) on reported non-HIF substrates. *Elife* **8**, e46490 (2019). 10.7554/eLife.46490 PMC6739866 31500697

[33] Zuker M (2003). Mfold web server for nucleic acid folding and hybridization prediction. Nucleic acids Res..

[34] Hofacker IL (2003). Vienna RNA secondary structure server. Nucleic acids Res..

[35] Li, B., Zhang, X. & Dong, Y. Nanoscale platforms for messenger RNA delivery. *Wiley Interdiscip Rev Nanomed Nanobiotechnol*, e1530 (2018). 10.1002/wnan.1530 PMC6443240 29726120

[36] Lubelsky Y, Ulitsky I (2018). Sequences enriched in Alu repeats drive nuclear localization of long RNAs in human cells. Nature.

[37] Dominguez C, Fisette JF, Chabot B, Allain FH (2010). Structural basis of G-tract recognition and encaging by hnRNP F quasi-RRMs. Nat. Struct. Mol. Biol..

[38] Arany Z (1996). An essential role for p300/CBP in the cellular response to hypoxia. Proc. Natl Acad. Sci. USA.

[39] Huertas P (2010). DNA resection in eukaryotes: deciding how to fix the break. Nat. Struct. Mol. Biol..

[40] Berra E (2003). HIF prolyl-hydroxylase 2 is the key oxygen sensor setting low steady-state levels of HIF-1alpha in normoxia. EMBO J..

[41] Kaelin WG, Ratcliffe PJ (2008). Oxygen sensing by metazoans: the central role of the HIF hydroxylase pathway. Mol. cell.

[42] Bedford DC, Kasper LH, Fukuyama T, Brindle PK (2010). Target gene context influences the transcriptional requirement for the KAT3 family of CBP and p300 histone acetyltransferases. Epigenetics.

[43] Christofk HR, Vander Heiden MG, Wu N, Asara JM, Cantley LC (2008). Pyruvate kinase M2 is a phosphotyrosine-binding protein. Nature.

[44] Fujita N (2012). Expression of prolyl hydroxylases (PHDs) is selectively controlled by HIF-1 and HIF-2 proteins in nucleus pulposus cells of the intervertebral disc: distinct roles of PHD2 and PHD3 proteins in controlling HIF-1alpha activity in hypoxia. J. Biol. Chem..

[45] Yang W (2011). Nuclear PKM2 regulates beta-catenin transactivation upon EGFR activation. Nature.

[46] Kopp F, Mendell JT (2018). Functional classification and experimental dissection of long noncoding RNAs. Cell.

[47] Lee JT (2012). Epigenetic regulation by long noncoding RNAs. Science.

[48] Geuens T, Bouhy D, Timmerman V (2016). The hnRNP family: insights into their role in health and disease. Hum. Genet.

[49] Harris AL (2002). Hypoxia–a key regulatory factor in tumour growth. Nat. Rev. Cancer.

[50] Semenza GL (2010). Defining the role of hypoxia-inducible factor 1 in cancer biology and therapeutics. Oncogene.

[51] Fraga A, Ribeiro R, Medeiros R (2009). [Tumor hypoxia: the role of HIF]. Actas urologicas espanolas.

[52] Mitchell PS (2008). Circulating microRNAs as stable blood-based markers for cancer detection. Proc. Natl Acad. Sci. USA.

[53] Tsai MC (2010). Long noncoding RNA as modular scaffold of histone modification complexes. Science.

[54] Langmead B, Trapnell C, Pop M, Salzberg SL (2009). Ultrafast and memory-efficient alignment of short DNA sequences to the human genome. Genome Biol..

[55] Galbraith MD (2013). HIF1A employs CDK8-mediator to stimulate RNAPII elongation in response to hypoxia. Cell.

[56] Heinz S (2010). Simple combinations of lineage-determining transcription factors prime cis-regulatory elements required for macrophage and B cell identities. Mol. cell.

[57] Ran FA (2013). Genome engineering using the CRISPR-Cas9 system. Nat. Protoc..

[58] Zheng F (2012). The putative tumour suppressor microRNA-124 modulates hepatocellular carcinoma cell aggressiveness by repressing ROCK2 and EZH2. Gut.

[59] Howat WJ (2014). Antibody validation of immunohistochemistry for biomarker discovery: recommendations of a consortium of academic and pharmaceutical based histopathology researchers. Methods.

[60] Kustatscher G, Wills KL, Furlan C, Rappsilber J (2014). Chromatin enrichment for proteomics. Nat. Protoc..

[61] Colaprico A (2016). TCGAbiolinks: an R/Bioconductor package for integrative analysis of TCGA data. Nucleic acids Res..

[62] Tang H (2014). Multiplexed parallel reaction monitoring targeting histone modifications on the QExactive mass spectrometer. Anal. Chem..

