# Supplementary material for: The HIF-1α antisense long non-coding RNA drives a positive feedback loop of HIF-1α mediated transactivation and glycolysis
Source: Nat Commun. 2021 Feb 26;12:1341. doi: 10.1038/s41467-021-21535-3 (PMC7910558; doi:10.1038/s41467-021-21535-3)
Supplement: Supplementary file 1 — Supplementary Information [file 41467_2021_21535_MOESM1_ESM.pdf]

Supplementary Information

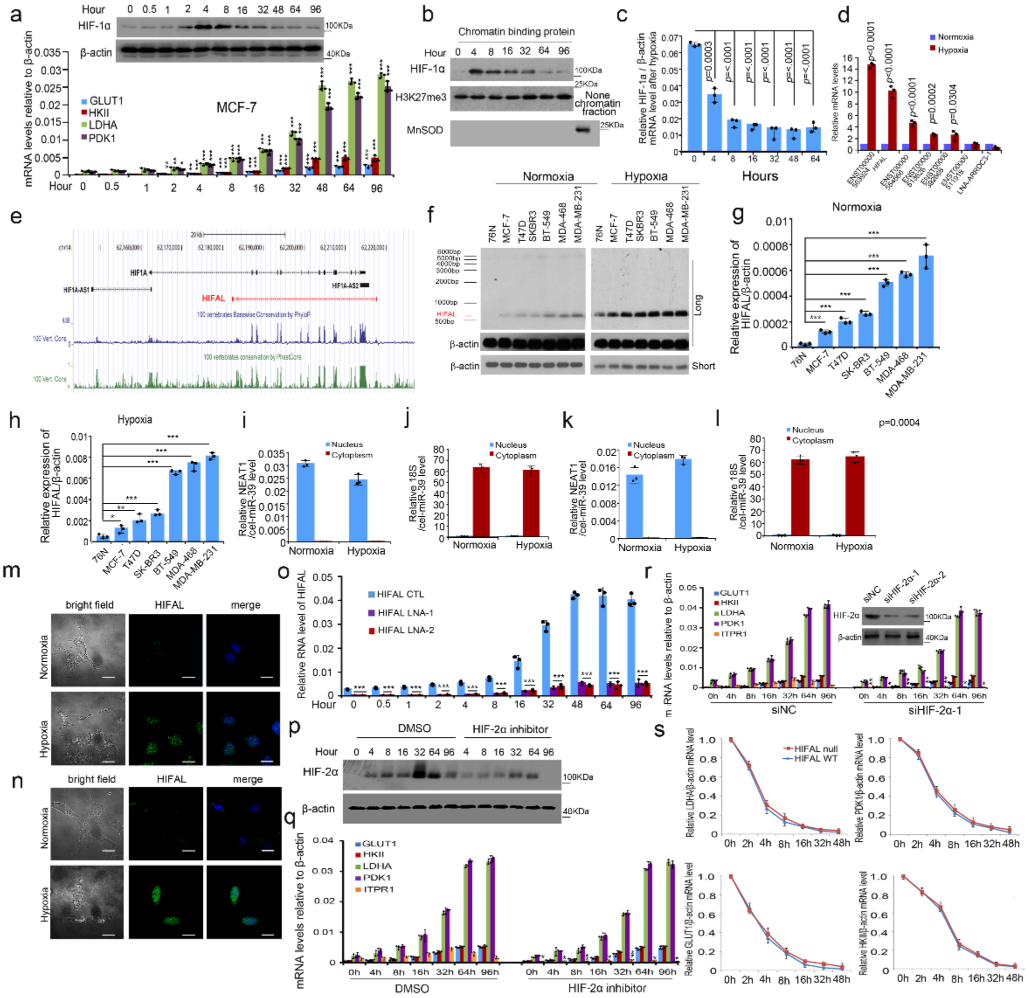

Supplementary Fig. 1. Hypoxia-induced HIFAL expression in breast cancer cell lines, related to Fig 1.

a, The kinetics of HIF-1α protein level under hypoxia, determined by immunoblotting, was not parallel with that of its target gene transcription revealed by qRT-PCR in MCF-7 cells.

b, The chromatin associated HIF-1α levels within the longation of hypoxic time.

c, HIF1 mRNA level declines with time following hypoxia. MDA-MB-231 cells were cultured under hypoxia and harvested at indicated time points for RNA preparation. HIF-1α mRNA level was measured by qRT-PCR and was normalized to β-actin mRNA level..

d,Differential expression of lncRNAs in MDA-MB-231 under normoxia and hypoxia. Experiments were performed three times, assayed by qRT-PCR.

e, Schematic graph for HIFAL as well as other HIF-1α antisense lncRNAs in HIF-1α genomic loci from UCSC browser.

f-h, HIFAL expression in breast cell lines under normoxia or hypoxia, assayed by northern blotting (f) and quantified in (g, h). The samples were derived from the same experiment and that gels/blots were processed in parallel.

i-l, Subcellular distribution of NEAT1 and 18S RNA，which shows that NEAT1 RNA is mostly localized in the nuclei and 18S RNA is mostly localized in the cytoplasm, demonstrating the quality of cytoplasmic or nuclear fractionation. The total RNA was prepared when cells were cultured in normoxia or hypoxia. The level of the indicated RNA was quantified by qRT-PCR and normalized to cel-miR, which was directly added

in to the prepared total RNA from cytoplasmic or nuclear fraction as an independent control. (i,j) for MCF-7 cells, (k,l) for MDA-MB-231 cells.

m,n, Confocal FISH images showing the localization of HIFAL in MCF7 (m) and MDA-MB-231(n) cells under normoxia and hypoxia. Scale bars, 10 m.

o, HIFAL knockdown is maintained during the time course of hypoxic condition.

p, The HIF-2α inhibitor (CAS882268-69-1) suppresses the protein levels of HIF-2α.

q,r, The knockdown and inhibitor of HIF-2α does not stimulate glycolytic gene expression in MDA-MB-231. ITPR as positive control.

s, HIFAL knockout does not affect the mRNA stability of the HIF-1 target genes. The RNA was prepared following indicated time points. qPCR was used to determine the mRNA levels of indicated genes.

For **a**, **c**, **d**, **g-l**, **o**, **q-s**, *p* values were determined by two-sided unpaired t-test. Graphs show means ± SD of experimental triplicates. **$p < 0.05$, **$p < 0.01$, ***$p<0.001$. Source data are provided as a Source Data file.

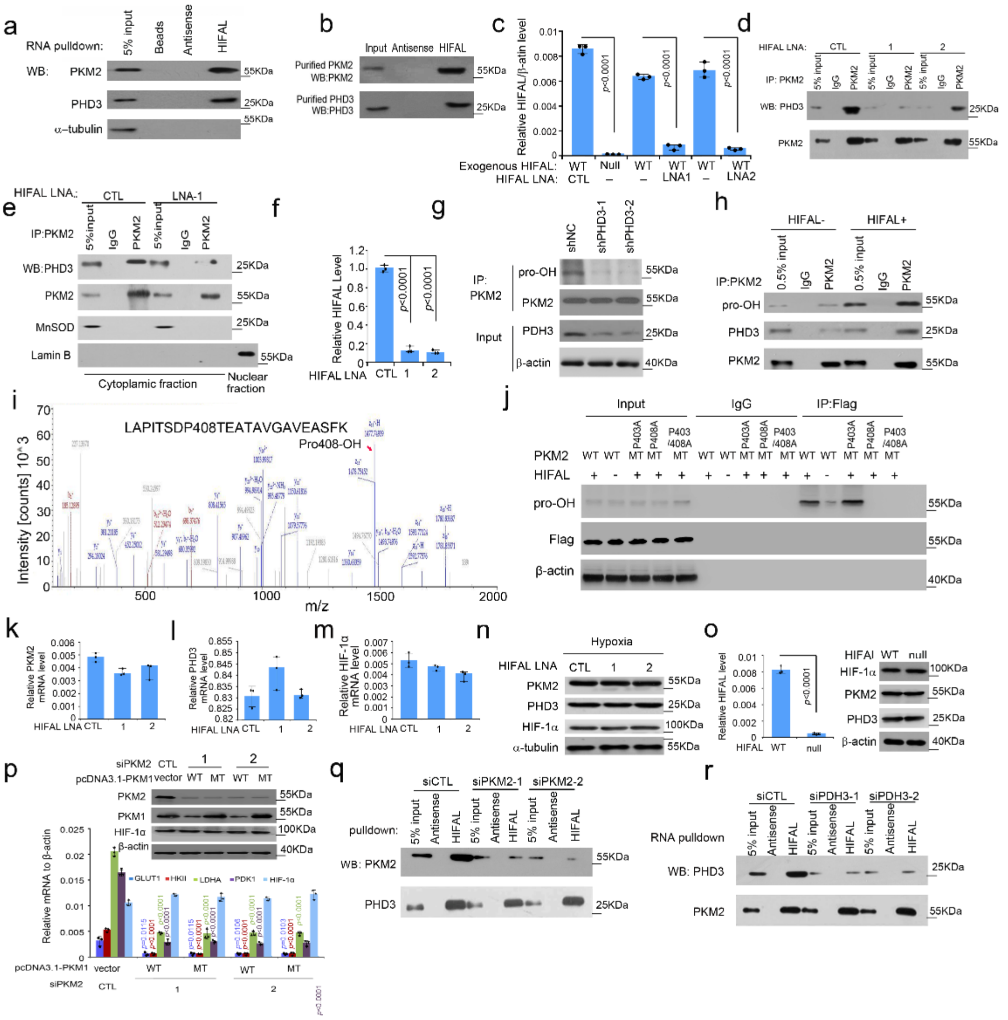

a
RNA pulldown:
5% input
Beads
Antisense
HIFAL
WB: PKM2
PHD3
α-tubulin
55KDa
25KDa
b
Input Antisense HIFAL
Purified PKM2 WB:PKM2
Purified PHD3 WB:PHD3
c
Relative HIFAL/β-atin level
p<0.0001
Exogenous HIFAL: WT Null WT WT WT WT
HIFAL LNA: CTL – – LNA1 – LNA2
d
HIFAL LNA: CTL 1 2
IP: PKM2
5% input IgG PKM2
WB: PHD3
PKM2
e
HIFAL LNA: CTL LNA-1
IP:PKM2
5%input IgG PKM2
WB:PHD3
PKM2
MnSOD
Lamin B
Cytoplamic fraction
Nuclear fraction
f
Relative HIFAL Level
p<0.0001
HIFAL LNA CTL 1 2
g
shNC shPHD3-1 shPHD3-2
IP: PKM2
pro-OH
PKM2
Input
PDH3
β-actin
40KDa
h
HIFAL- HIFAL+
IP:PKM2
0.5% input IgG PKM2
pro-OH
PHD3
PKM2
i
LAPITSDP408TEATAVGAVEASFK
Pro408-OH
Intensity [counts] 10^3
m/z
j
Input IgG IP:Flag
PKM2 WT WT P403A MT P408A MT P403/408A MT
HIFAL + - + + +
pro-OH
Flag
β-actin
k
Relative PKM2 mRNA level
HIFAL LNA CTL 1 2
l
Relative PHD3 mRNA level
HIFAL LNA CTL 1 2
m
Relative HIF-1α mRNA level
HIFAL LNA CTL 1 2
n
Hypoxia
HIFAL LNA CTL 1 2
PKM2
PHD3
HIF-1α
α-tubulin
100KDa
o
Relative HIFAL level
p<0.0001
HIFAL WT null
HIFAl WT null
HIF-1α
PKM2
PHD3
β-actin
p
siPKM2 CTL 1 2
pcDNA3.1-PKM1 vector WT MT WT MT
PKM2
PKM1
HIF-1α
β-actin
Relative mRNA to β-actin
GLUT1 HKII LDHA PDK1 HIF-1α
pcDNA3.1-PKM1 vector WT MT WT MT
siPKM2 CTL 1 2
q
siCTL siPKM2-1 siPKM2-2
pulldown:
5% input Antisense HIFAL
WB: PKM2
PHD3
r
siCTL siPDH3-1 siPDH3-2
RNA pulldown
5% input Antisense HIFAL
WB: PHD3
PKM2

Supplementary Fig. 2. HIFAL binds to PKM2 and PHD3 under hypoxia, related to Fig. 2.

a, RNA pulldown shows that HIFAL specifically binds with PKM2, PHD3. The MDA-MB-231 cells were cultured under hypoxia and the cell lysates were prepared for RNA pulldown assay, followed by immunoblotting of PKM2 and PHD3. α-tubulin was used as controls.

b, In vitro interaction between HIFAL and PKM2, or PHD3, shown by RNA pulldown. Purified PKM2 and PHD3 were used in this assay.

c, The HIFAL levels in the HIFAL KO MDA-MB-231 cells transfected with exogenous HIFAL or HIFAL LNAs.

d, Knockdown of HIFAL reduces the binding of PKM2 to PHD3. Cells were transiently transfected with HIFAL-LNAs and then cultured under hypoxia and lysed for PKM2 precipitation. The precipitation was probed with anti-PHD3 or PKM2 antibodies by western blotting.

e, Knockdown of HIFAL decreases the cytoplasmic interaction of PHD3 with PKM2. Cells were transiently transfected with HIFAL-LNAs for 48 hours, and then cultured under hypoxia, cytoplasmic and nuclear protein was extracted. The PKM2 precipitation using cytoplasmic fraction was probed with PHD3 or PKM2 antibodies by western blotting.

f, Quantified HIFAL levels in (d) and (e).

g, Knockdown of PHD3 reduces the hydroxylation of PKM2. Cells were transiently transfected with shPHD3 for 48 hours, and then cultured under hypoxia and lysed for

PKM2 precipitation. The precipitation was probed with anti-hydroxylation or PKM2 antibodies by western blotting.

h, Enforced expression of HIFAL extremely increases prolyl hydroxylation of the purified PHD3 towards PKM2. HIFAL plasmid was transiently transfected into HIFAL KO MDA-MB-231cells for 48 hours, and then cultured under hypoxia and lysed for PKM2 precipitation.

i, The purified recombinant PKM2 and PHD3 mixture was incubated without/with HIFAL and were analyzed by mass spectrometry. The fragmentation spectrum of 401LAPITSDPTEATAVGAVEASFK422 revealed the hydroxylation of Pro-408 within this peptide.

j, HIFAL recruits PHD3 to promotes the proxyl hydroxylation of Pro408 in PKM2. Hela cells were transfected with Flag-PKM2 WT or mutant (P403A, P408A, P403/408A) and HIFAL.

k-m, mRNA levels of PKM2, PHD3 and HIF-1α following HIFAL knockdown in MDA-MB-231 cells under hypoxia for 48 hr. Experiments were performed three times, assayed by qRT-PCR.

n, Protein expression of PKM2, PHD3 and HIF-1α in HIFAL knockdown MDA-MB-231 cells under hypoxia for 48 hr.

o, Protein expression of PKM2, PHD3 and HIF-1α in HIFAL knockout or WT MDA-MB-231 cells under hypoxia for 48 hr.

p, The mRNA level of GLUT1, HK2, LDHA, PDK1 and HIF-1α in PKM2 knockdown or PKM1 overexpression MDA-MB-231 cells under hypoxia for 48 hr.

q, r, Knockdown of PKM2 (q) or PHD3 (r) does not reduce the binding of HIFAL to PHD3 or PKM2. Cells were transiently transfected with siRNA targeting PKM2 or PHD3 for 48 hours, and then cultured under hypoxia and lysed for HIFAL RNA pulldown. The precipitation was probed with anti-PHD3 or PKM2 antibodies by western blotting as indicated.

For **c**,**g**, **k-m**, **o**, **p,** *p* values were determined by two-sided unpaired t-test. Graphs show means ± SD of experimental triplicates. Source data are provided as a Source Data file

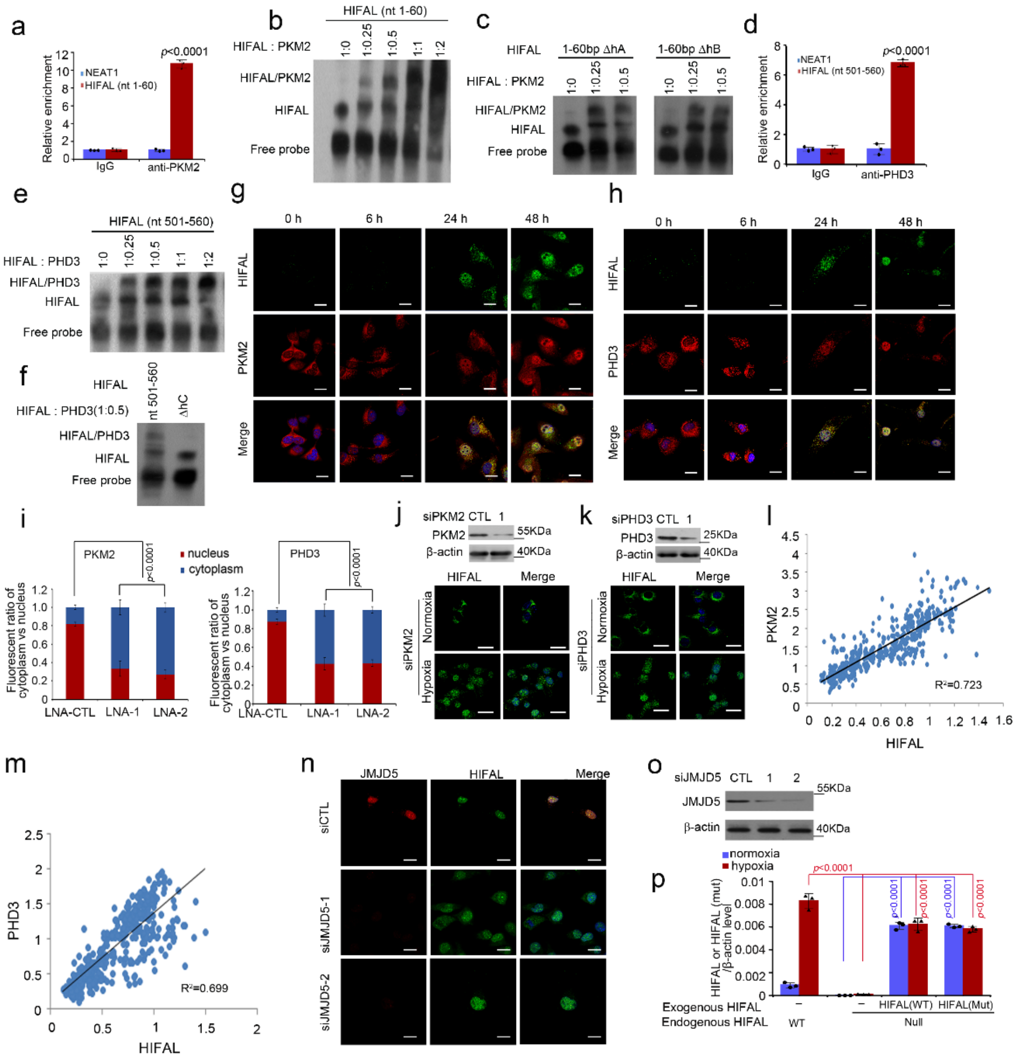

a
Relative enrichment
NEAT1
HIFAL (nt 1-60)
p<0.0001
IgG
anti-PKM2
b
HIFAL (nt 1-60)
HIFAL : PKM2
1:0
1:0.25
1:0.5
1:1
1:2
HIFAL/PKM2
HIFAL
Free probe
c
HIFAL
1-60bp ΔhA
1-60bp ΔhB
HIFAL : PKM2
HIFAL/PKM2
HIFAL
Free probe
d
Relative enrichment
NEAT1
HIFAL (nt 501-560)
p<0.0001
IgG
anti-PHD3
e
HIFAL (nt 501-560)
HIFAL : PHD3
HIFAL/PHD3
HIFAL
Free probe
f
HIFAL
HIFAL : PHD3(1:0.5)
nt 501-560
ΔhC
HIFAL/PHD3
HIFAL
Free probe
g
0 h
6 h
24 h
48 h
HIFAL
PKM2
Merge
h
0 h
6 h
24 h
48 h
HIFAL
PHD3
Merge
i
PKM2
PHD3
nucleus
cytoplasm
p<0.0001
Fluorescent ratio of cytoplasm vs nucleus
LNA-CTL
LNA-1
LNA-2
j
siPKM2 CTL 1
PKM2
β-actin
55KDa
40KDa
HIFAL
Merge
Normoxia
Hypoxia
siPKM2
k
siPHD3 CTL 1
PHD3
β-actin
25KDa
40KDa
HIFAL
Merge
Normoxia
Hypoxia
siPHD3
l
PKM2
HIFAL
R²=0.723
m
PHD3
HIFAL
R²=0.699
n
JMJD5
HIFAL
Merge
siCTL
siJMJD5-1
siJMJD5-2
o
siJMJD5 CTL 1 2
JMJD5
β-actin
55KDa
40KDa
p
normoxia
hypoxia
p<0.0001
HIFAL or HIFAL (mut) /β-actin level
Exogenous HIFAL
Endogenous HIFAL
HIFAL(WT)
HIFAL(Mut)
WT
Null

Supplementary Fig. 3. HIFAL binds to PKM2/PHD3 and promotes the translocation of PKM2/PHD3 into the nucleus, related to Fig. 2 and 3.

a, RNA immunoprecipitation assay shows that HIFAL fragment (nt 1-60) binds to PKM2. MCF-7 cells were transiently transfected with HIFAL fragment nt 1-60, and then cultured under hypoxia. Cell lysates were immunoprecipitated with anti-PKM2 antibody. The level of HIFAL fragment in the precipitate was examined by qRT-PCR.

b, RNA EMSA shows the interaction of HIFAL fragment (nt 1-60) with PKM2. HIFAL:PKM2 indicates the molar ratio of HIFAL vs PKM2.

c, RNA EMSA demonstrating the binding capacity of PKM2 to mutant HIFAL segments (nt 1-60) deleting hairpin A or hairpin B. HIFAL:PKM2 indicates molar ratio.

d, RNA immunoprecipitation assay demonstrates that HIFAL fragment (nt 501-560) binds toPHD3. MCF-7 cells were transiently transfected with HIFAL fragment, and then cultured under hypoxia. Cell lysates were immunoprecipitated with anti-PHD3 antibody. The level of HIFAL fragment in the precipitate was examined by qRT-PCR.

e, RNA EMSA demonstrates the interaction of HIFAL fragment (nt 501-560) with PHD3. HIFAL:PHD3 indicates the molar ratio of HIFAL vs PHD3.

f, RNA EMSA demonstrating the binding capacity of PHD3 to mutant HIFAL (nt 501-560) deleting hairpin C. HIFAL:PHD3 indicates molar ratio.

g, h, HIFAL promotes the translocation of PKM2 and PHD3 into the nucleus. MDA-MB-231 cell were immunostained with anti-PKM2 (g) or anti-PHD3 (h) (red), anti-digoxin for HIFAL probe (green) and DAPI (blue) at indicated time points following hypoxia. Scale bars, 10 μm.

i, Quantification of nuclear and cytoplasmic distribution of PKM2 and PHD3 in HIFAL knockdown and control cells in the immunofluorescence experiment. Error bars shows mean ± SD of fifteen random fields. *P* values were determined by two-sided unpaired t-test

j, k, Knockdown of PKM2 (j) or PHD3 (k) does not affect the nuclear translocation of HIFAL. Cells were transiently transfected with PKM2 or PHD3-siRNAs for 48 hours, and then cultured under hypoxia. The upper panel shows the knockdown efficiency of PKM2 or PHD3. Scale bars, 10 μM.

l, m, Correlation of nuclear HIFAL expression with nuclear PKM2 (l) and PDH3 (m).

n, o, Silencing JMJD5 does not affect the nuclear translocation of HIFAL. Cells were transiently transfected with JMJD5-siRNAs for 48 hours, and then cultured under hypoxia. The immunostaining of JMJD5 and HIFAL hybridized with digoxin labeled probes was shown in (n) and knockdown efficiency of JMJD5 was shown in (o). Scale bars, 10 μM.

p, The HIFAL levels in the HIFAL WT or null MDA-MB-231 cells transfected with exogenous WT HIFAL or Mutant HIFAL RNAs (deletion of nt 240-300).

For a, d, p, *p* values were determined by two-sided unpaired t-test. Error bars shows mean ± SD of experimental triplicates. Source data are provided as a Source Data file

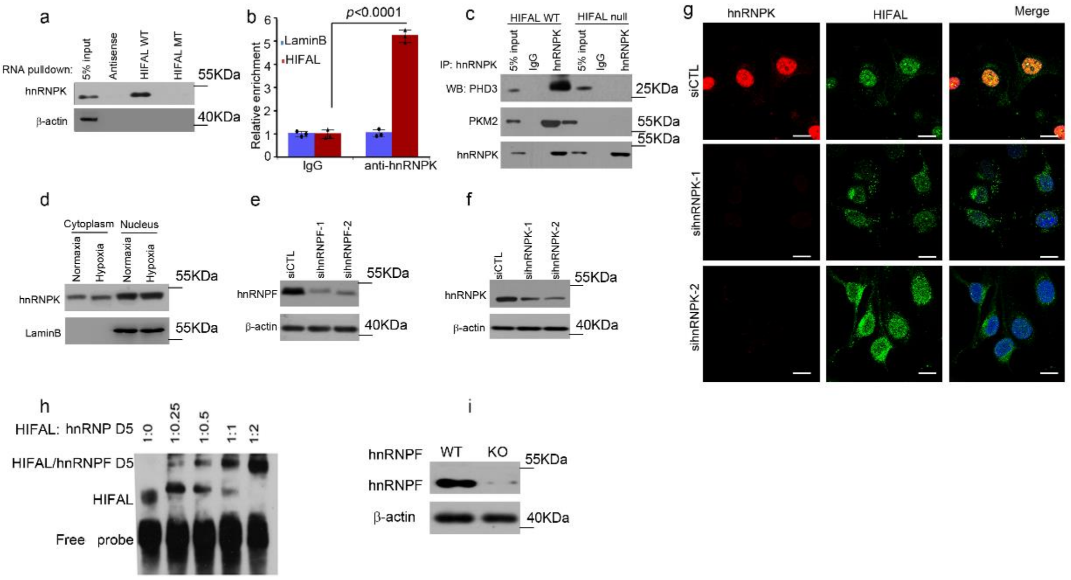

a
RNA pulldown:
5% input
Antisense
HIFAL WT
HIFAL MT
55KDa
hnRNPK
β-actin
40KDa
b
p<0.0001
LaminB
HIFAL
Relative enrichment
IgG
anti-hnRNPK
c
HIFAL WT
HIFAL null
IP: hnRNPK
5% input
IgG
hnRNPK
WB: PHD3
25KDa
PKM2
55KDa
hnRNPK
55KDa
g
hnRNPK
HIFAL
Merge
siCTL
sihnRNPK-1
sihnRNPK-2
d
Cytoplasm
Nucleus
Normaxia
Hypoxia
hnRNPK
LaminB
55KDa
e
siCTL
sihnRNPF-1
sihnRNPF-2
hnRNPF
β-actin
55KDa
40KDa
f
siCTL
sihnRNPK-1
sihnRNPK-2
hnRNPK
β-actin
55KDa
40KDa
h
HIFAL: hnRNP D5
1:0
1:0.25
1:0.5
1:1
1:2
HIFAL/hnRNPF D5
HIFAL
Free probe
i
hnRNPF
WT
KO
55KDa
hnRNPF
β-actin
40KDa

Supplementary Fig. 4. HIFAL promotes the translocation of PKM2 and PHD3 to the nucleus, related to Fig. 4.

a, b, RNA pulldown and RNA immunoprecipitation assay demonstrates that hnRNPK binds with WT HIFAL but not mutated HIFAL without nuclear translocation domain (without nt 240-300). Bar graphs represent means ± SD of experimental triplicates. p< 0.0001 versus IgG precipitation. two-sided unpaired t-test.

c, The binding of PKM or PHD3 with hnRNPK depends on HIFAL. The immunoprecipitation of hnRNPK with PKM2 or PHD3 was performed in HIFAL WT cells or HIFAL null MDA-MB-231 cells. hnRNPK does not bind with PKM2 or PHD3 in HIFAL-null MDA-MB-231 cells.

d, hnRNPK expression and nuclear translocation does not rely on hypoxia. MDA-MB-231 cells were cultured under normoxia or hypoxia and then the cytoplasmic or nuclear fractions were prepared. Western blot showed that nuclear hnRNPK was upregulated under hypoxia.

e, f, Immunoblotting for hnRNPF (e) and hnRNPK (f) expression in MDA-MB-231 cells transfected with siRNAs.

g, HIFAL nuclear translocation is not affected by silencing hnRNPK. MDA-MB-231 cells were transiently transfected with hnRNPK-siRNAs for 24 hours, and then cultured under hypoxia for 48 hours. HIFAL (green) was detected by digoxin labeled probes. hnRNPK were immunostained in red with antibodies. Nuclei were stained with DAPI (blue). Scale bars, 10 μM.

h, RNA EMSA shows the interaction of HIFAL fragment (nt 240-300) with hnRNPF.

HIFAL: hnRNPF indicates the molar ratio of HIFAL vs hnRNPF.

i, The hnRNPF levels in the hnRNPF WT or KO MDA-MB-231 cells are determined by immunoblotting. Source data are provided as a Source Data file

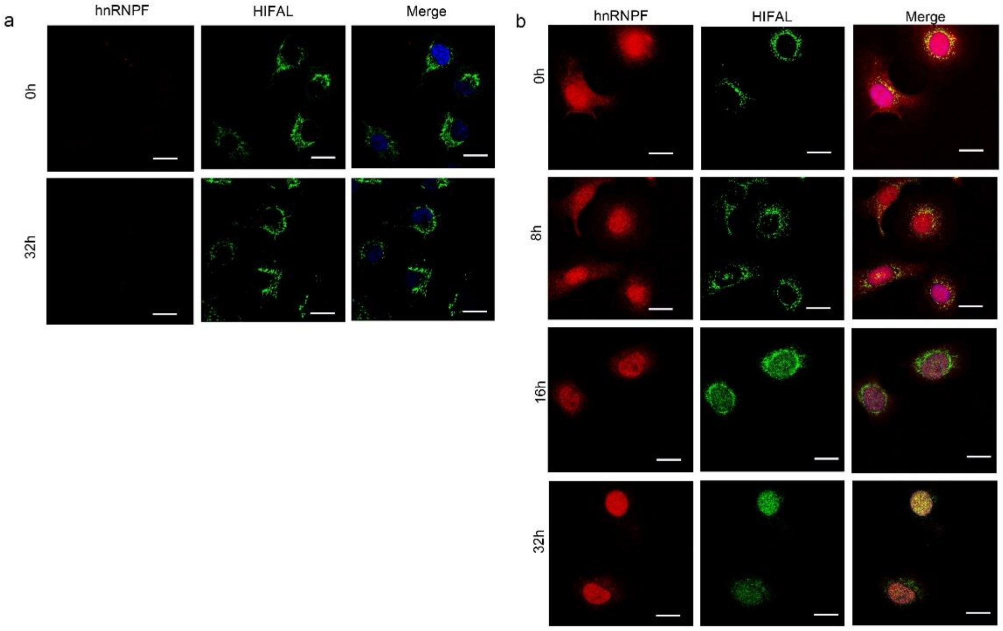
a
hnRNPF
HIFAL
Merge
0h
32h
b
hnRNPF
HIFAL
Merge
0h
8h
16h
32h

Supplementary Fig. 5. The exogenous HIFAL can be translocated into the nucleus in MDA-MB-231 WT cells, related to Fig. 4.

a, b, The exogenous HIFAL were transfected into hnRFNP WT (a) or knockout (b) MDA-MB-231 cells and cultured under hypoxia for indicated hours. HIFAL (green) was detected by biotin labeled probes. hnRFNP was immunostained in red with antibodies. Nuclei were stained with DAPI (blue). Scale bars, 10 μM. Source data are provided as a Source Data file.

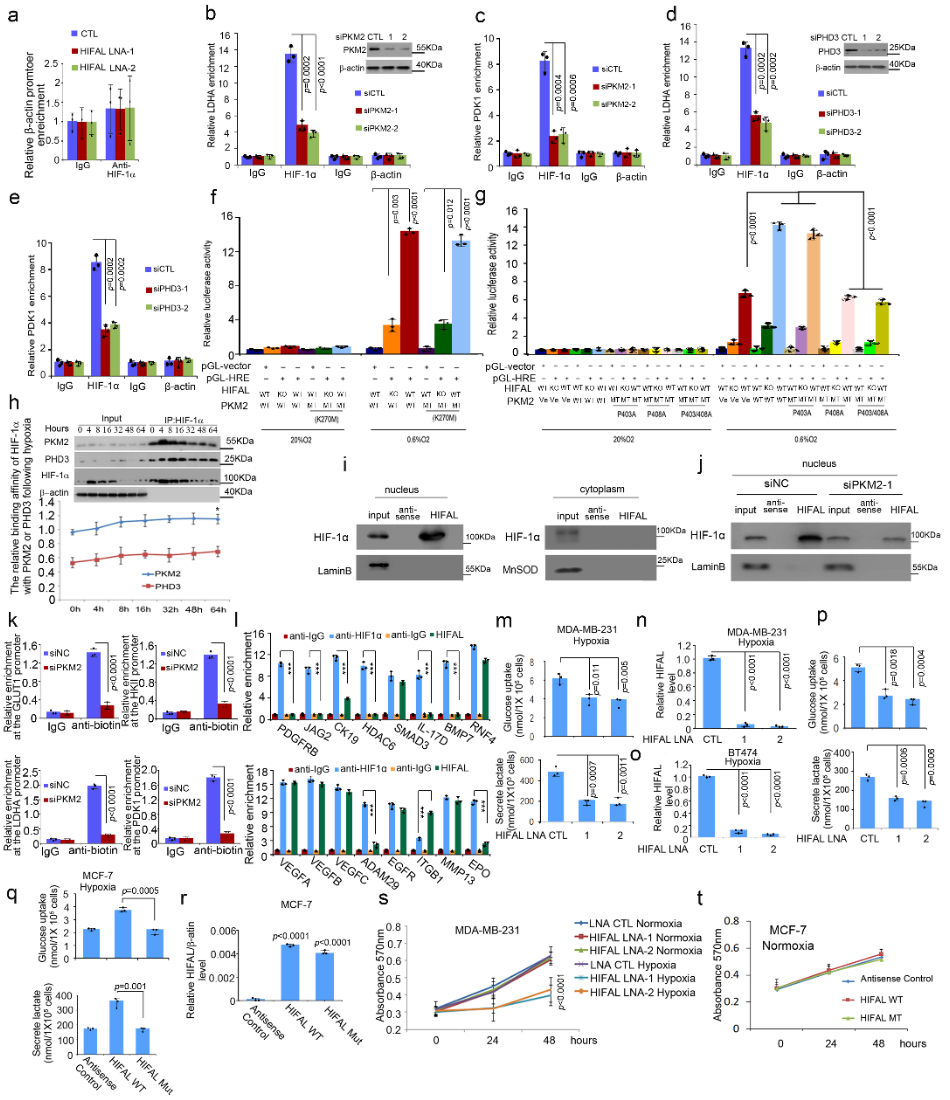

a
CTL
HIFAL LNA-1
HIFAL LNA-2
Relative β-actin promtoer enrichment
IgG
Anti-HIF-1α
b
siPKM2 CTL 1 2
PKM2
β-actin
55KDa
40KDa
Relative LDHA enrichment
siCTL
siPKM2-1
siPKM2-2
IgG
HIF-1α
β-actin
c
Relative PDK1 enrichment
d
siPHD3 CTL 1 2
PHD3
25KDa
40KDa
siPHD3-1
siPHD3-2
e
f
Relative luciferase activity
pGL-vector
pGL-HRE
HIFAL
PKM2
(K270M)
20%O2
0.6%O2
g
P403A
P408A
P403/408A
h
Hours
Input
IP:HIF-1α
PKM2
PHD3
HIF-1α
β-actin
100KDa
The relative binding affinity of HIF-1α with PKM2 or PHD3 following hypoxia
0h
4h
8h
16h
32h
48h
64h
i
nucleus
cytoplasm
input
anti-sense
HIFAL
LaminB
MnSOD
j
siNC
siPKM2-1
k
Relative enrichment at the GLUT1 promoter
Relative enrichment at the HK1 promoter
Relative enrichment at the LDHA promoter
Relative enrichment at the PDK1 promoter
siNC
siPKM2
anti-biotin
l
anti-IgG
anti-HIF1α
Relative enrichment
PDGFRB
JAG2
CK19
HDAC6
SMAD3
IL-17D
BMP7
RNF4
VEGFA
VEGFB
VEGFC
ADAM29
EGFR
ITGB1
MMP13
EPO
m
MDA-MB-231
Hypoxia
Glucose uptake (nmol/1X 10⁵ cells)
Secrete lactate (nmol/1X10⁵ cells)
HIFAL LNA
n
Relative HIFAL level
o
BT474
Hypoxia
p
q
MCF-7
Hypoxia
Antisense Control
HIFAL WT
HIFAL Mut
r
MCF-7
Relative HIFAL/β-atin level
s
MDA-MB-231
Absorbance 570nm
LNA CTL Normoxia
HIFAL LNA-1 Normoxia
HIFAL LNA-2 Normoxia
LNA CTL Hypoxia
HIFAL LNA-1 Hypoxia
HIFAL LNA-2 Hypoxia
hours
t
MCF-7
Normoxia
Antisense Control
HIFAL WT
HIFAL MT

Supplementary Fig. 6. The binding affinity of HIF1α, PKM2 to HIF1α target genes and the glycolysis and proliferation of breast cancer cells were affected by HIFAL, related to Figure 6.

a, HIF-1α ChIP experiments demonstrate that HIF-1α does not effectively enrich the promoter of β-actin, which is not affected by knocking down HIFAL as well. CTL vs LNA-1 p=0.98, CTL vs LNA-2 p=0.97, two-sided unpaired t-test.

b-e,Silencing PKM2 or PHD3 results in the decreased enrichment of HIF-1α to its target genes. MDA-MB-231cells were transiently transfected with siPKM2 or siPHD3 and cultured under hypoxia for 48 hours, and then collected for ChIP analyses. Real-time PCR data were calibrated to IgG control and normalized with sample inputs of chromatin harvested prior to immunoprecipitation. The PKM2 or PHD3 levels are shown in the upper panel of (b) and (d).

f, PKM2 (K270M) increases HIF-1α transcriptional activity similarly to WT PKM2. HIFAL WT or KO MDA-MB-231 cells were co-transfected with pGL-vector or pGL-HRE, HIFAL, PKM2 WT or PKM2(K270M) and were exposed to 20% or 0.6% O2 for 24h.

g, The mutation of Pro408A and the double mutation of Pro403/408A significantly reduce the HIFAL-mediate HIF-1α transactivation, whereas the mutation of Pro403A does not. HIFAL WT or KO MDA-MB-231 cells were co-transfected with pGL-vector or pGL-HRE, HIFAL, PKM2 WT or PKM2 (P403A, P408A, P403/408A) and were exposed to 20% or 0.6% O2 for 24h.

h, The binding capacity of HIF-1α to PKM2 and PHD3 does not significantly increase

with time following hypoxia upon HIFAL knockout. Co-immunoprecipitation (Co-IP) of HIF-1α with PKM2 or PHD3 was performed at indicated time points following hypoxia in the HIFAL-null MDA-MB-231 cells, and normalized by the corresponding HIF-1α protein levels. PKM2 0h vs 64h p=0.0143, PHD3 0h vs 64h p=0.0585.

i, The interaction of HIFAL with HIF-1α in nucleus and cytoplasm under hypoxic condition.

j, Knockdown of PKM2 decreased the interaction of HIFAL with HIF-1α in nucleus.

k, Knockdown of PKM2 decreased the interaction of HIFAL with the promoter of HIF-1α targeting genes GLUT1, HKII, LDHA and PDK1. PKM2 were silenced in HIFAL KO MDA-MB-231 cells. Then the biotin labeled HIFAL were transfected into MDA-MB-231 cells and cultured under hypoxia for 48hs, and collected for HIF1α target gene promoter ChIP-PCR analyses with anti-biotin antibody or control IgG.

l, HIFAL-negative/HIF1a-positive genes (up) and eight HIFAL/HIF1a-double positive genes (down) in ChIP-seq assays were chosen to validate the binding of HIFAL or HIF-1α with indicated genes promoters using ChIP-PCR.

m,n, HIFAL knockdown decreases glucose uptake and lactate secretion in MDA-MB-231 cells under hypoxia condition. Cells were transfected with LNAs and cultured under hypoxia for 24 hr. Levels of glucose uptake and lactate secretion into the culture media were measured and normalized to cell number. The relative HIFAL levels are shown in (n). .

o,p, HIFAL knockdown decreases glucose uptake and lactate secretion under hypoxia conditions in BT474. Cells were transfected with LNAs and cultured under normoxia

and hypoxia for 24 hr. Levels of glucose uptake and lactate secretion into the culture media were measured and normalized to cell number. The relative HIFAL levels are shown in (**o**)..

q, r, HIFAL overexpression increases glucose uptake and lactate secretion in MCF-7 cells under hypoxia. MCF-7 cells were transfected by the HIFAL plasmid, and the expression of HIFAL WT or mutant was detected by qRT-PCR (**r**). Levels of glucose uptake and lactate secretion into the culture medium were measured and normalized to cell number.

s, The cell viability of MDA-MB-231 decreases upon HIFAL knockdown under hypoxia but is not affected under normoxia. Cells were cultured under normoxic or hypoxic condition and measured by MTT assay at 0hr, 24hr, and 48hr.

t, HIFAL overexpression does not affect the viability of MCF-7 cells under normoxia.

For **a**-**h**, **k**-**t**, *p* values were determined by unpaired two-tailed *t* test. Graphs show means ± SD of experimental triplicates. *p<0.05,**p<0.01， ***p<0.001. Source data are provided as a Source Data file

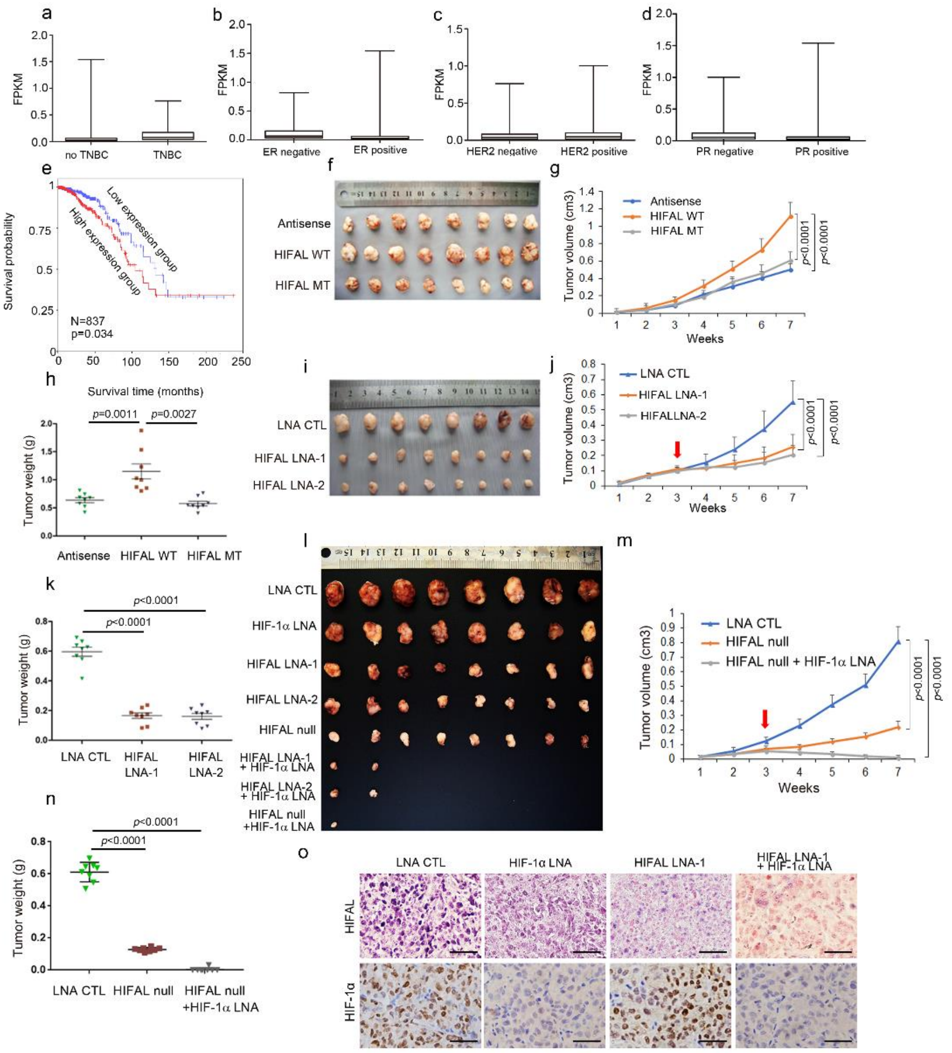
a
b
c
d
FPKM
no TNBC
TNBC
ER negative
ER positive
HER2 negative
HER2 positive
PR negative
PR positive
e
Survival probability
Low expression group
High expression group
N=837
p=0.034
Survival time (months)
f
Antisense
HIFAL WT
HIFAL MT
g
Tumor volume (cm3)
Weeks
p<0.0001
h
Tumor weight (g)
p=0.0011
p=0.0027
i
LNA CTL
HIFAL LNA-1
HIFAL LNA-2
j
HIFALLNA-2
k
p<0.0001
l
HIF-1α LNA
HIFAL null
HIFAL LNA-1 + HIF-1α LNA
HIFAL LNA-2 + HIF-1α LNA
HIFAL null +HIF-1α LNA
m
HIFAL null + HIF-1α LNA
n
o
HIFAL
HIF-1α

Supplementary Fig. 7. Inhibiting HIFAL and HIF-1α synergistically reduces tumor burden in nude mice, related to Fig. 7.

a-d, HIFAL expression in different subtypes of breast cancer in TCGA breast cancer dataset. (**a**: no TNBC n=644, TNBC n=84; **b**: ER negative n=174, ER positive n=598; **c**: HER2 negative n=424, HER2 positive n=127; **d**: PR negative n=244, PR positive n=524). Alteration of expression is shown as box plot presentations, with the y axis indicating HIFAL expression.

e, High HIFAL expression correlates with poor survival in the TCGA breast cancer dataset (N=837, *p*=0.034, Kaplan–Meier, log-rank test).

f-h, Enforced expression of wild-type, but not mutant HIFAL, promotes the growth of MCF-7 xenografts. MCF-7 cells with ectopic expression of HIFAL antisense control(antisense), wild-type (WT) or mutant (MT) HIFAL with the PKM2 binding motif (hairpin A or B) deleted were implanted subcutaneously into the mammary fat pads of nude mice (n=8 per group). Data shown are mean ± SD (n = 8 per group).*P* values were determined by two-sided unpaired t-test.

i-k, Knockdown of HIFAL suppresses the growth of MDA-MB-231 xenografts. MDA-MB-231 cells were implanted subcutaneously into the mammary fat pads of nude mice. Mice (n=8 per group) were treated with intraperitoneal injection of LNAs (CTL, LNA-1, LNA-2) after the tumor volumes reached 100 mm^3. Data shown are mean ± SD. *P* values were determined by two-sided unpaired t-test.

l-n, HIF-1α-LNA synergistically suppresses the growth of MDA-MB-231 xenografts with HIFAL knockout or knockdown. Mice (n=8 per group) were treated with LNA

CTL(CTL), HIF-1α-LNA   HIFAL null (HIFAL null) or/and   HIF-1α-LNA (HIFAL null+ HIF-1α-LNA) after the tumor volumes reached 100 mm^3. Data shown are mean ± SD (n = 8 per group). *P* values were determined by one-way ANOVA+ Dunnett's post hoc tests.

o, Representative images for the in-situ hybridization of HIFAL and immunostaining of HIF-1α on the paraffin-embedded sections of xenografts. Scale bars, 20 μm. Source data are provided as a Source Data file.

Supplementary Table 1. Sequences of HIFAL, siRNAs, LNAs, primers sgRNAs and mutants

**HIFAL sequence characterized by RACE**
TCATGTAGCGCCAGCCACACCAGGAACAGAAGGGTGCCGGGTACCTTCCGCATGCTTGGTATTCTCCCCGCGGGGCTCTGACCGCTGCCGCTCTCAGGCACCTGTCTTTCCTCTCCGTCCCAGAATGGAGCCAAGACAAGGGAATAAACGAAATTCAATAGTACACGGAGATCGGGTGTCTGGGCAGCGTCTTGGAAAAACTATCCACTACAGGCTGGAGTGCAATGGCGAGATCTTGGTTCGCTGCAACCTCCGCTTCCCGGGTTCAAGCGGTTCTCCTGCCTCAGCCTCCTGAGTAGCTGGGATTGAGTACAGGTCAAGTGAAGTTCTTCTGCTGATATGTTCTGCTACTGCATAAGAGACGGAATCTATGTTGCCTCGACTGGTCCTGAACTCCTGGGCTAAAGCGATCCTTCTGCCTTGGCCTCCCAAAGTGCGAGGATTATAGGTGTGAGCCACCCAACCTGGCCTTTGCCAGTATTTTTAAATCCAAACACCTATGCATGGTGCTTACTAATAACACAGTGGTATTCTAAACATTTTTCACCTATTATTTAATCTTTATTATTATCACCATGTTACAGATAAGGACCCTGGCTAAATAGCTTACCCAACATTACTCCGTTAATAAATGGTAGAACCTGTATTTCACTCAAAAA

**siRNA**	**sense**	**antisense**
siHIF-1α-1	CGAGGAAGAACUAUGAACATT	UGUUCAUAGUUCUUCCUCGTT
siHIF-1α-2	GAUGAAAGAAUUACCGAAUTT	AUUCGGUAAUUCUUUCAUCTT
siPKM2-1	GGCUGGACUACAAGAACAUTT	AUGUUCUUGUAGUCCAGCCTT
siPKM2-2	GUGGUGAUCUAGGCAUUGATT	UCAAUGCCUAGAUCACCACTT
siPHD3-1	GGGAAAUGGAACAGGUUAUTT	AUAACCUGUUCCAUUUCCCTT
siPHD3-2	GCAUCUACUAUCUGAACAATT	UUGUUCAGAUAGUAGAUGCTT
siJMJD5-1	GAUUUGAGCUUCUCGGUCATT	UGACCGAGAAGCUCAAAUCTTUG
siJMJD5-2	AAGUUGGUUCGAGGUACATT	UACCUCGAACCAACUUCTT
siHNRNPK-1	GGGUUGUAGAGUGCAUAAATT	UUUAUGCACUCUACAACCCTT
siHNRNPK-2	GUCAGCGGAUUAAACAAAUTT	AUUUGUUUAAUCCGCUGACTT
siHNRNPF-1	CCGCAGGUGUCCAUUUCAUTT	AUGAAAUGGACACCUGCGGTT
siHNRNPF-2	GGUACAUUGAGGUGUUCAATT	UUGAACACCUCAAUGUACCTT
siHIF-2α-1	CUCCUCAGUUUGCUCUGAATT	UUCAGAGCAAACUGAGGAGTT
siHIF-2α-2	CAGAACUGAUUGGUUACCATT	UGGUAACCAAUCAGUUCUGTT

**LNA GapmeR sequences for the knockdown of HIFAL and HIF-1α**	
HIFAL LNA1	CATTCTGGGACGGAGA
HIFAL LNA2	GTAACATGGTGATAAT
HIF1α-1 LNA	TGGCAAGCATCCTGTA
Control LNA	AACACGTCTATACGC

Primers for qRT-PCR

**Name**	**Forward (5’-3’)**	**Reverse (5’-3’)**
Actin	AACTCCATCATGAAGTGTGACG	GATCCACATCTGCTGGAAGG
HIF-1α	TCACCACAGGACAGTACAGGATGC	CCAGCAAAGTTAAAGCATCAGGTTC
HIFAL	GTACACGGAGATCGGGTGTC	GGAGTTCAGGACCAGTCGAG
Neat1	GCTGGAGTCTTGGGCACGGC	TCAACCGAGGCCGCTGTCTC
ENST00000	CTCTTCCGGGGAAGTCTGA’	AAGAAGGGCAGCAAATTCAC

563924		
ENST00000554566	GCCAGAAACCCCAATAACCT	TTCGTTTAAGGGTCGGAATG
ENST00000513626	AGTCCTGGAGGCCGTATTTT	CCATATTCCATTCCCCACTG
ENST00000392668	AGGATCCTGTGGAAGGGTCT	CAGCTCCAGCTATCCACCTC
ENST00000511918	TGCACTTTTCCAGCACAAAC	AAGATGCTGCTGTTGCTCCT
ARRDC3-1	CTCCGTTTTCCTCCATTTCA	ACCTTGAAGGAGGTGGGACT
GLUT1	TGGCATCAACGCTGTCTTCT	CTAGCGCGATGGTCATGAGT
PKM2	GTCTGGGAGGAAAGTCGCTC	GGCGGAAGGACACAGATTCA
PHD3	CCTGTCTGCACGAGGCAAT	GCACTTCGTGTGGGTTCCTA
PDK1	TGCTGTATGGCCTGCAAGAT	ACATTCTGGCTGGTGACAGG
HKII	GCAACTACCTGGAACCTGCAA	AGTCATGCACAGGGTTGGTAGA
LDHA	GGAGGACCCAGCAATTAGTC	GCCATTAACTGCCACAAAGC
18S	GCCGCTAGAGGTGAAATTCTTG	CTTTCGCTCTGGTCCGTCTT
Neat1	CAGGGTGTCCTCCACCTTTA	AAACCAGCAGACCCCTTTTT
Cel-miR-39	TCACCGGGTGTAAATCAGCTTG	UNIVERSAL REVERSE PRIMER
HIF-2α	GTGCTCCCACGGCCTGTA	TTGTCACACCTATGGCATATCACA
ITPR1	GTGACAGGAAACATGCAGACTC	CAGCAGTTGCACAAAGACAGGC
PRIMERS FOR CHIP ASSAY (5'-3')		
**Name**	**Forward (5'-3')**	REVERSE (5'-3')
HIFAL(B)	CGTACACGAAAGTCGCCTT	GCTACATGATGAGGATGCGAAA
HFAL(A)	TTTCGCATCCTCATCATGTA	TTATTCTTACCTGTAGTGGATA
HIFAL(C)	AAGTATTATATAACTAACCA	TTTGTCTTCTGGGACTTGTCAAA
LDHA(A)	TTGGAGGGCAGCACCTTACTTAGA	GCCTTAAGTGGAACAGCTATGCTGA
LDHA(B)	CGTCTCTACTAAAAATTACAA	TTGAAACGGAGTCGCTCTGTC
LDHA(C)	CAGAGATTGAAGTGAGCCAAGAT	ATACGCTTTATAGCTGTGTCCAAC
PDK1	CGCGTTTGGATTCCGTG	CCAGTTATAATCTGCCTTCCCTATC
GLUT1	CTCAGTGGCTCACGCCTGTAAT	CCTCCCGAGTAGCTGGGATTACA
HKII	GCCCCGCAGGTAGTCAGG	GACCACGATTCTCTCCACG
VEGFA	ATCCATCCTGTCTTGATATAGAA	AACAGCTCTCTCCTGTCTCCT
VEGFB	ATTGCAGGAAGGTGAAGCAGAT	TTGTCGCTCCTGCTGAAGGAT
VEGFC	TATCATGAAGACACTGATCTTT	TGATTCACTTGGTCATAAATT
ADAM29	TTAGTAATGTGGTAACCTGGACT	TTTCATATATGCTTCATCTA
ITGB1	GAGAGATAATAGGAAAACAAGCT	AATCTCTATCTCCATTATCTCCT
MMP13	TTCTACCTCTGTCTGAATCTGTA	TTCTCTAGAATAACTACAGAGA
EPO	TGGTCAATAAGGTGGCTCCATT	AGAGACCAAAGAAGAGGCAGAAA
EGFR	TAAGCATCTATACTAGCCTGGTAT	TTCCAGGTCTCCTCAGGCAGGA
RNF4	ACCAAGGAGCTGTCTGCTCA	AGTGCTGACTCCCACTCAGA
BMP7	TAGGCTCGACTACAAAGGTCCT	ATTTGCACTGGACAGTGTCAGC
IL-17D	AGGAAATGTCCTTTATTCACATGC	ACAGGAATGTGTTCTAGACTTA

SMAD3	AGATATACCTTGACCAATGATGT	AATGGCACTGCAGAGATAGCAT
HDAC6	AAGGCAAGAAAACTAGCAGGAAG	TTGAGCTGCCAAAGCAATTCTCA
CK19	AGATTTAGTTTTCTTTCTACTC	CTCTACCATGTAGTTGCTGTGT
PDGFRB	TTAACTGTGTGAAGGCTGGTT	AATTTGAATTTGTTGAACT
JAG2	TTCTCCATTGTATTGAGCCA	AGTCATGGTGTACACGACTCCTCA
Primers for 5’ RACE and 3’RACE (5’-3’)		
5’-1	CCAGGGTCCTTATCTGT	
5’-2	TTCGTTTATTCCCTTGTCTTGGCT	
3’-1	ATGGAGCCAAGACAAGGGAATAA	
3’-2	GGCAGCGTCTTGGAAAAACTATC	
3’-3	ACAGATAAGGACCCTGGCTAAAT	
**LNA probe for FISH, ISH and northern blot**		
HIFAL	ACGGAGTAATGTTGGGTAAGCT	
**Sequences of ΔmA, ΔmB, ΔmC**		
ΔmA	TCAGCCACCAGGAACAGAAGGGTGCCGGGTACTTCCGCATGCTTGGT	
ΔmB	TCATGTAGCGCCAGCCACACCAGGAACAGTACCTGGT	
ΔmC	TGCATGTCTAAACATTTTTCACCTATTATTTAATC	
**Sequences of sgRNA for the knockout of HIFAL**		
SgRNA1	CACCGGAAGTTTCTCGGGTTGGCTT	
SgRNA2	CACCGGCTCGGGAAGTTTCTCGGGT	
SgRNA3	CACCGCTGCGCCCGAGCACGTACTG	
SgRNA4	CACCGATTTGCTTGGGAATTTCGGG	
The sequence of mutant HIFAL without PKM2 binding motif		
	TCAGCCACCAGGAACAGTACCTGGTATTCTCCCCGCGGGGCTCTGACCGCT<br>GCCGCTCTCAGGCACCTGTCTTTCCTCTCCGTCCCAGAATGGAGCCAAGAC<br>AAGGGAATAAACGAAATTCAATAGTACACGGAGATCGGGTGTCTGGGCAG<br>CGTCTTGGAAAAACTATCCACTACAGGCTGGAGTGCAATGGCGAGATCTTGG<br>TTCGCTGCAACCTCCGCTTCCCGGGTTCAAGCGGTTCTCCTGCCTCAGCCTCCT<br>GAGTAGCTGGGATTGAGTACAGGTCAAGTGAAGTTCTTCTGCTGATATGTTC<br>TGCTACTGCATAAGAGACGGAATCTATGTTGCCTCGACTGGTCCTGAACTC<br>CTGGGCTAAAGCGATCCTTCTGCCTTGGCCTCCCAAAGTGCGAGGATTATA<br>GGTGTGAGCCACCCAACCTGGCCTTTGCCAGTATTTTTAAATCCAAACACC<br>TATGCATGGTGCTTACTAATAACACAGTGGTATTCTAAACATTTTTCACCT<br>ATTATTTAATCTTTATTATTATCACCATGTTACAGATAAGGACCCTGGCTAA<br>ATAGCTTACCCAACATTACTCCGTTAATAAATGGTAGAACCTGTATTTCACT<br>CAAAAA	
The sequence of hnRNPF mutant		
D RRM	ATGATGCTGGGCCCTGAGGGAGGTGAAGGCTTTGTGGTCAAGCTCCGTGGCCTGCC<br>CTGGTCCTGCTCTGTTGAGGACGTGCAGAACTTCCTCTCTGACTGCACGATTCATG<br>ATGGGGCCGCAGGTGTCCATTTCATCTACACTAGAGAGGGCAGGCAGAGTGGTGA<br>GGCTTTTGTTGAACTTGGATCAGAAGATGATGTAAAAATGGCCCTGAAAAAAGAC<br>AGGGAAAGCATGGGACACAAGTCCCACAGAACCGAGATGGATTGGGTGTTGAAGC<br>ACAGTGGTCCCAACAGTGCCGACAGCGCCAACGATGGCTTCGTGCGGCTTCGAGG	

	ACTCCCATTTGGATGCACAAAGGAAGAAATTGTTCAGTTCTTCTCAGGGTTGGAAATTGTGCCAAACGGGATCACATTGCCTGTGGACCCCGAAGGCAAGATTACAGGGGAAGCGTTCGTGCAGTTTGCCTCGCAGGAGTTAGCTGAGAAGGCTCTAGGGAAACACAAGGAGAGGATAGGGCACAAGAGCAGCCAGGAGGAAGTTAGGTCATACTCAGATCCCCCTCTGAAGTTCATGTCCGTGCAGCGGCCAGGGCCCTATGACCGGCCCGGGACTGCCAGGAGGTACATTGGCATCGTGAAGCAGGCAGGCCTGGAAAGGATGAGGCCTGGTGCCTACAGCACAGGCTACGGGGGCTACGAGGAGTACAGTGGCCTCAGTGATGGCTACGGCTTCACCACCGACCTGTTCGGGAGAGACCTCAGCTACTGTCTCTCCGGAATGTATGACCACAGATACGGCGACAGTGAGTTCACAGTGCAGAGCACCACAGGCCACTGTGTCCACATGAGGGGCCTGCCGTACAAAGCGACCGAGAACGACATTTACAACTTCTTCTCTCCTCTCAACCCTGTGAGAGTCCATATTGAGATTGGCCCAGATGGAAGAGTGACGGGTGAAGCAGATGTTGAGTTTGCTACTCATGAAGAAGCTGTGGCAGCTATGTCCAAAGACAGGGCCAATATGCAGCACTTGAATTCAACAACAGGGGCCAGCAATGGGGCGTATAGCAGCCAGGTGATGCAAGGCATGGGGGTGTCTGCTGCCCAGGCCACTTACAGTGGCCTGGAGAGCCAGTCAGTGAGTGGCTGTTACGGGGCCGGCTACAGTGGGCAGAACAGCATGGGTGGCTATGACTAG
D1	ATGATGCTGGGCCCTGAGGGAGGTGAAGGCTTTGTGGTCAAGCTCCGTGGCCTGCCCTGGTCCTGCTCTGTTGAGGACGTGCAGAACTTCCTCTCTGACTGCACGATTCATGATGGGGCCGCAGGTGTCCATTTCATCTACACTAGAGAGGGCAGGCAGAGTGGTGAGGCTTTTGTTGAACTTGGATCAGAAGATGATGTAAAAATGGCCCTGAAAAAAGACAGGGAAAGCATGGGACAC
D2	AAGTCCCACAGAACCGAGATGGATTGGGTGTTGAAGCACAGTGGTCCCAACAGTGCCGACAGCGCCAACGATGGCTTCGTGCGGCTTCGAGGACTCCCATTTGGATGCACAAAGGAAGAAATTGTTCAGTTCTTCTCAGGGTTGGAAATTGTGCCAAACGGGATCACATTGCCTGTGGACCCCGAAGGCAAGATTACAGGGGAAGCGTTCGTGCAGTTTGCCTCGCAGGAGTTAGCTGAGAAGGCTCTAGGGAAACACAAGGAGAGGATAGGGCAC
D3	AAGAGCAGCCAGGAGGAAGTTAGGTCATACTCAGATCCCCCTCTGAAGTTCATGTCCGTGCAGCGGCCAGGGCCCTATGACCGGCCCGGGACTGCCAGGAGGTACATTGGCATCGTGAAGCAGGCAGGCCTGGAAAGGATGAGGCCTGGTGCCTACAGCACAGGCTACGGGGGCTACGAGGAGTACAGTGGCCTCAGTGATGGCTACGGCTTCACCACCGACCTGTTCGGGAGAGACCTCAGCTACTGTCTCTCCGGAATGTATGACCACAGATACGGCGACAGTGAGTTCACAGTGCAGAGCACCACAGGCCACTGTGTCCACATGAGGGGCCTGCCGTACAAAGCGACCGAGAACGACATTTACAACTTCTTCTCTCCTCTCAACCCTGTGAGAGTCCATATTGAGATTGGCCCAGATGGAAGAGTGACGGGTGAAGCAGATGTTGAGTTTGCTACTCATGAAGAAGCTGTGGCAGCTATGTCCAAAGACAGGGCCAATATGCAGCACTTGAATTCAACAACAGGGGCCAGCAATGGGGCGTATAGCAGCCAGGTGATGCAAGGCATGGGGGTGTCTGCTGCCCAGGCCACTTACAGTGGCCTGGAGAGCCAGTCAGTGAGTGGCTGTTACGGGGCCGGCTACAGTGGGCAGAACAGCATGGGTGGCTATGACTA
D4	AAGTCCCACAGAACCGAGATGGATTGGGTGTTGAAGCACAGTGGTCCCAACAGTGCCGACAGCGCCAACGATGGCTTCGTGCGGCTTCGAGGACTCCCATTTGGATGCACAAAGGAAGAAATTGTTCAGTTCTTCTCAGGGTTGGAAATTGTGCCAAACGGGATCACATTGCCTGTGGAC
D5	ATCACATTGCCTGTGGACCCCGAAGGCAAGATTACAGGGGAAGCGTTCGTGCAGT

	TTGCCTCGCAGGAGTTAGCTGAGAAGGCTCTAGGGAAACACAAGGAGAGGATAGG<br>GCAC
D6	ATGATGCTGGGCCCTGAGGGAGGTGAAGGCTTTGTGGTCAAGCTCCGTGGCCTGCC<br>CTGGTCCTGCTCTGTTGAGGACGTGCAGAACTTCCTCTCTGACTGCACGATTCATG<br>ATGGGGCCGCAGGTGTCCATTTCATCTACACTAGAGAGGGCAGGCAGAGTGGTGA<br>GGCTTTTGTTGAACTTGGATCAGAAGATGATGTAAAAATGGCCCTGAAAAAAGAC<br>AGGGAAAGCATGGGACACCGGTACATTGAGGTGTTCAAGTCCCACAGAACCGAGA<br>TGGATTGGGTGTTGAAGCACAGTGGTCCCAACAGTGCCGACAGCGCCAACGATGG<br>CTTCGTGCGGCTTCGAGGACTCCCATTTGGATGCACAAAGGAAGAAATTGTTCAGT<br>TCTTCTCAGGGTTGGAAATTGTGCCAAACGGGAGGTACATTGAGGTGTTTAAGAGC<br>AGCCAGGAGGAAGTTAGGTCATACTCAGATCCCCCTCTGAAGTTCATGTCCGTGCA<br>GCGGCCAGGGCCCTATGACCGGCCCGGGACTGCCAGGAGGTACATTGGCATCGTG<br>AAGCAGGCAGGCCTGGAAAGGATGAGGCCTGGTGCCTACAGCACAGGCTACGGGG<br>GCTACGAGGAGTACAGTGGCCTCAGTGATGGCTACGGCTTCACCACCGACCTGTTC<br>GGGAGAGACCTCAGCTACTGTCTCTCCGGAATGTATGACCACAGATACGGCGACA<br>GTGAGTTCACAGTGCAGAGCACCACAGGCCACTGTGTCCACATGAGGGGCCTGCC<br>GTACAAAGCGACCGAGAACGACATTTACAACTTCTTCTCTCCTCTCAACCCTGTGA<br>GAGTCCATATTGAGATTGGCCCAGATGGAAGAGTGACGGGTGAAGCAGATGTTGA<br>GTTTGCTACTCATGAAGAAGCTGTGGCAGCTATGTCCAAAGACAGGGCCAATATGC<br>AGCACAGATATATAGAACTCTTCTTGAATTCAACAACAGGGGCCAGCAATGGGGC<br>GTATAGCAGCCAGGTGATGCAAGGCATGGGGGTGTCTGCTGCCCAGGCCACTTAC<br>AGTGGCCTGGAGAGCCAGTCAGTGAGTGGCTGTTACGGGGCCGGCTACAGTGGGC<br>AGAACAGCATGGGTGGCTATGACTAG
HIFAL binding mut	ATGATGCTGGGCCCTGAGGGAGGTGAAGGCTTTGTGGTCAAGCTCCGTGGCCTGCC<br>CTGGTCCTGCTCTGTTGAGGACGTGCAGAACTTCCTCTCTGACTGCACGATTCATG<br>ATGGGGCCGCAGGTGTCCATTTCATCTACACTAGAGAGGGCAGGCAGAGTGGTGA<br>GGCTTTTGTTGAACTTGGATCAGAAGATGATGTAAAAATGGCCCTGAAAAAAGAC<br>AGGGAAAGCATGGGACACCGGTACATTGAGGTGTTCAAGTCCCACAGAACCGAGA<br>TGGATTGGGTGTTGAAGCACAGTGGTCCCAACAGTGCCGACAGCGCCAACGATGG<br>CTTCGTGCGGCTTCGAGGACTCCCATTTGGATGCACAAAGGAAGAAATTGTTCAGT<br>TCTTCTCAGGGTTGGAAATTGTGCCAAACGGGATCACATTGCCTGTGGACAGGTAC<br>ATTGAGGTGTTTAAGAGCAGCCAGGAGGAAGTTAGGTCACCTCTGAAGTTCATGTC<br>CGTGCAGCGGCCAGGGCCCTATGACCGGCCCGGGACTGCCAGGAGGTACATTGGC<br>ATCGTGAAGCAGGCAGGCCTGGAAAGGATGAGGCCTGGTGCCTACAGCACAGGCT<br>ACGGGGGCTACGAGGAGTACAGTGGCCTCAGTGATGGCTACGGCTTCACCACCGA<br>CCTGTTCGGGAGAGACCTCAGCTACTGTCTCTCCGGAATGTATGACCACAGATACG<br>GCGACAGTGAGTTCACAGTGCAGAGCACCACAGGCCACTGTGTCCACATGAGGGG<br>CCTGCCGTACAAAGCGACCGAGAACGACATTTACAACTTCTTCTCTCCTCTCAACC<br>CTGTGAGAGTCCATATTGAGATTGGCCCAGATGGAAGAGTGACGGGTGAAGCAGA<br>TGTTGAGTTTGCTACTCATGAAGAAGCTGTGGCAGCTATGTCCAAAGACAGGGCCA<br>ATATGCAGCACAGATATATAGAACTCTTCTTGAATTCAACAACAGGGGCCAGCAAT<br>GGGGCGTATAGCAGCCAGGTGATGCAAGGCATGGGGGTGTCTGCTGCCCAGGCCA<br>CTTACAGTGGCCTGGAGAGCCAGTCAGTGAGTGGCTGTTACGGGGCCGGCTACAG<br>TGGGCAGAACAGCATGGGTGGCTATGACTAG

Sequences of sgRNA for the knockout of hnRNPF	
SgRNA1	CACCGAGTGCCGACAGCGCCAACGA

Supplementary Table 2. Univariate and multivariate Cox proportional hazard analysis of prognostic variables in 493 cases of invasive breast cancer patients

**Variable**	**HR (95% CI)**	**χ2**	**P value**
Univariate analysis			
Tumor size			
≤2	1.655(1.067-2.565)	5.066	0.024
>2			
HIFLA	1.875(1.201-2.927)	7.646	0.006
SI≤3			
SI＞3			
Her2	1.326(0.852-2.063)	1.567	0.211
(-)			
(+)			
Ki67	1.195(0.751-1.902)	0.568	0.451
≤14%			
>14%			
PCNA	1.544(0.938-2.541)	2.92	0.087
≤50%			
>50%			
Histological grade	1.347(1.015-1.789)	4.242	0.039
I-II			
III			
Stage	1.406(1.062-1.862)	5.651	0.017
I-II			
III			
LN metastasis	1.228(1.05-1.437)	6.575	0.01
(-)			
(+)			
Mutivariate analysis			
HIFAL(SI>3 or ≤3)	1.702(1.081-2.68)	5.265	0.022
LN metastasis	1.182(1.007-1.387)	4.201	0.04

Supplementary Table 3. Correlation of HIFLA expression with clinicopathologic status in 493 cases of patients with invasive breast cancer

		HIFAL			
**Variables**	**All cases**	**SI≤3**	**SI＞3**	**χ2**	***P* value***
Tumor size (cm)				5.169	0.023
≤2	299	170	129		
>2	194	90	104		
Stage				40.469	0.000
I	219	149	70		
II	182	81	101		
III	92	30	62		
Histological grade				30.901	0.000
I	234	153	81		
II	180	80	100		
III	79	27	52		
ER				2.732	0.098
(-)	201	97	104		
(+)	292	163	129		
Her2				0.662	0.416
(-)	295	160	135		
(+)	198	100	98		
Ki67				7.147	0.008
≤14%	187	113	74		
>14%	306	147	159		
PCNA				5.327	0.021
≤50%	163	98	65		
>50%	330	162	168		
LN metastasis				9.327	0.009
0	158	93	65		
1-3	226	123	103		
4	109	44	65		
Metastasis				8.239	0.004
(-)	466	253	213		
(+)	27	7	20		

Supplementary Table 4. Correlation of HIFLA expression with clinicopathologic status in 292 cases of patients with luminal invasive breast cancer

	HIFLA				
**Variables**	**All cases**	**SI≤3**	**SI＞3**	**$\chi 2$**	***P* value***
Tumor size (cm)				4.353	0.037
≤2	178	108	70		
>2	114	55	59		
Stage				16.354	0.000
I	126	87	39		
II	115	55	60		
III	51	21	30		
Histological grade				13.822	0.001
I	140	93	47		
II	103	51	52		
III	49	19	30		
Ki67				4.659	0.031
≤14%	113	72	41		
>14%	179	91	88		
PCNA				6.821	0.009
≤50%	96	64	32		
>50%	196	99	97		
LN metastasis				9.079	0.011
0	91	60	31		
1-3	130	73	57		
4	71	30	41		
Metastasis				5.916	0.016
(-)	279	160	119		
(+)	13	3	10		

Supplementary Table 5. Correlation of HIFLA expression with clinicopathologic status in 99 cases of patients with basal-like invasive breast cancer

	HIFLA				
**Variables**	**All cases**	**SI≤3**	**SI＞3**	**χ2**	***P* value***
Tumor size (cm)				8.614	0.003
≤2	62	39	23		
>2	37	12	25		
Stage				17.146	0.000
I	40	30	10		
II	37	16	21		
III	22	5	17		
Histological grade				16.227	0.000
I	33	26	7		
II	44	19	25		
III	22	6	16		
Ki67				4.55	0.033
≤14%	33	22	11		
>14%	66	29	37		
PCNA				3.607	0.058
≤50%	34	22	12		
>50%	65	29	36		
LN metastasis				8.677	0.013
0	24	12	12		
1-3	50	32	18		
4	25	7	18		
Metastasis				6.639	0.01
(-)	87	49	38		
(+)	12	2	10		
