# Supplementary material for: The HIF-1α antisense long non-coding RNA drives a positive feedback loop of HIF-1α mediated transactivation and glycolysis
Source: Nat Commun. 2021 Feb 26;12:1341. doi: 10.1038/s41467-021-21535-3 (PMC7910558; doi:10.1038/s41467-021-21535-3)
Supplement: Supplementary file 2 — Reporting summary [file 41467_2021_21535_MOESM2_ESM.pdf]

nature research

Corresponding author(s): Hai Hu, Erwei Song

Last updated by author(s): Jan 8, 2021

Reporting Summary

Nature Research wishes to improve the reproducibility of the work that we publish. This form provides structure for consistency and transparency in reporting. For further information on Nature Research policies, see our Editorial Policies and the Editorial Policy Checklist.

Statistics

For all statistical analyses, confirm that the following items are present in the figure legend, table legend, main text, or Methods section.

n/a	Confirmed	
☐	☒	The exact sample size (*n*) for each experimental group/condition, given as a discrete number and unit of measurement
☐	☒	A statement on whether measurements were taken from distinct samples or whether the same sample was measured repeatedly
☐	☒	The statistical test(s) used AND whether they are one- or two-sided<br>*Only common tests should be described solely by name; describe more complex techniques in the Methods section.*
☒	☐	A description of all covariates tested
☐	☒	A description of any assumptions or corrections, such as tests of normality and adjustment for multiple comparisons
☐	☒	A full description of the statistical parameters including central tendency (e.g. means) or other basic estimates (e.g. regression coefficient) AND variation (e.g. standard deviation) or associated estimates of uncertainty (e.g. confidence intervals)
☐	☒	For null hypothesis testing, the test statistic (e.g. *F*, *t*, *r*) with confidence intervals, effect sizes, degrees of freedom and *P* value noted<br>*Give P values as exact values whenever suitable.*
☒	☐	For Bayesian analysis, information on the choice of priors and Markov chain Monte Carlo settings
☒	☐	For hierarchical and complex designs, identification of the appropriate level for tests and full reporting of outcomes
☒	☐	Estimates of effect sizes (e.g. Cohen's *d*, Pearson's *r*), indicating how they were calculated

Our web collection on statistics for biologists contains articles on many of the points above.

Software and code

Policy information about availability of computer code

Data collection	Fluorescent images: Laser scanning confocal microscopy (LSM710 or 780, Zeiss).<br>qRT-PCR: LightCycler 480 instrument (Roche). Seahorse XFe24 Analyzer: Wave 2.4.<br>DNA fragments were end-repaired using the DNA End-Repair Kit. Then a single “A” base was added using Klenow fragment (New England Biolabs). The fragments were ligated to Illumina Indexed adaptors (NEBNext® Multiplex Oligos for Illumina kit) using T4 DNA ligase (New England Biolabs). The ligated products were enriched by magnetic bead to remove the unligated adaptors, and were subjected to 16 PCR cycles (NEBNext® Multiplex Oligos for Illumina Index Primers). PCR product was purified by magnetic bead. Libraries were quantified using a Qubit fluorometer (Invitrogen) and by quantitative PCR (TAKARA Biosystems). Two barcoded libraries were pooled and sequenced to 150bp in a single lane on an Illumina HiSeq2000 using standard procedures for cluster amplification and sequencing by synthesis.
Data analysis	Data representation and statistical analysis: Graphpad Prism 7.0; Kaplan-Meier prognostic analysis: SPSS (version 16.0). Laser scanning confocal microscopy: ZEN 2012,IVIS Lumina Imaging: Living Image software ver. 3.0.<br>Raw 150-nucleotide ChIP-seq reads were subjected to quality assessment (97% bases ≥Q30) using FASTQC v0.11.8 (https://www.bioinformatics.babraham.ac.uk/projects/fastqc/) and adapter sequences (~13%) were filtered out using the FASTX toolkit (http://hannonlab.cshl.edu/fastx_toolkit/index.html). Reads were then aligned to the reference human genome (GRCh37/hg19) using Bowtie 0.12.7 with zero-mismatches and discarding non-unique alignments . Enriched HIF1a and biotin ChIP peak regions were determined using MACS2 v2.1.1.3 with both ChIP and control (input) samples . Motif enrichment was performed using HOMER 52. Overlapped unique peaks were identified by using BED tools v2.29.0. Metagenes were created by taking the mean number of reads per 150 bp bin across all regions as indicated.

For manuscripts utilizing custom algorithms or software that are central to the research but not yet described in published literature, software must be made available to editors and reviewers. We strongly encourage code deposition in a community repository (e.g. GitHub). See the Nature Research guidelines for submitting code & software for further information.

Data

Policy information about availability of data

All manuscripts must include a data availability statement. This statement should provide the following information, where applicable:

- Accession codes, unique identifiers, or web links for publicly available datasets
- A list of figures that have associated raw data
- A description of any restrictions on data availability

The expression profile microarray data of breast cancer cells and tissues and CHIP-seq have been desposited in public database under the accession code GSE159490 and CRA003355. The microarray and CHIP-seq data referenced during the study are available in a public repository from the website (https://www.ncbi.nlm.nih.gov/geo/query/acc.cgi?acc=GSE159490 and https://bigd.big.ac.cn/gsa/s/Z9K8598l). The source data underlying Figs. 1a-l, 2a-q, and 3c, d, j, k and 4e-h,k,l,m and 5a-m, 6a-m, 7a,b,d-i, Supplementary Figs.1a-d, f-l,o-s and 2a-r, 3a-f, i-m, o,p and 4a-i, 6a-t and 7a-e, g,h,j,k,m,n e are provided as a Source Data file. All the other data supporting the findings of this study are available within the article and its supplementary information files and from the corresponding author upon reasonable request. A reporting summary for this article is available as a Supplementary Information file.

Field-specific reporting

Please select the one below that is the best fit for your research. If you are not sure, read the appropriate sections before making your selection.

☒ Life sciences ☐ Behavioural & social sciences ☐ Ecological, evolutionary & environmental sciences

For a reference copy of the document with all sections, see nature.com/documents/nr-reporting-summary-flat.pdf

Life sciences study design

All studies must disclose on these points even when the disclosure is negative.

Sample size	Sample sizes of the patients were counted on the basis of sample availability and it is sufficient for analysis. The sample size of experiment in vitro and in vivo was based previously publication(PMID: 30224822). Each experiment in vitro repeated three times under the same condition, and mouse n>5 was used in vivo.experiment according to our experimental purpose.
Data exclusions	No data were excluded, and all samples were included in data analysis.
Replication	All experiments were experimental triplicates or independently repeated over three times with similar results as stated in the Figure Legends. Most of attempt at replication were successful. A minority of results was discouraged because the anaerobic tank leaked.
Randomization	For cell experiments, all cells were allocated from the same parental cells and treated with the same indicated methods. For all animals in each group of the same gender, age, weight and genetic background were randomized . For Figures 7i and 7l animal studies,MDA-MB-231 cells were injected into 115 mice ,the number of which is more than the actual used mice number in order to remove the mice with the biggest and smallest tumors. When tumors reached 100 mm3, remaining mice with tumors were randomized according to tumor volume, body weight, and so on.
Blinding	In vitro CHIP -seq and Animal immunohistochemical staining were blinded by one group. The data analysis and Immunohistochemical scores were processed and analyzed by another blinded co-author mentioned in Author contributions.

Reporting for specific materials, systems and methods

We require information from authors about some types of materials, experimental systems and methods used in many studies. Here, indicate whether each material, system or method listed is relevant to your study. If you are not sure if a list item applies to your research, read the appropriate section before selecting a response.

Materials & experimental systems

n/a	Involved in the study	
☐	☒	Antibodies
☐	☒	Eukaryotic cell lines
☒	☐	Palaeontology and archaeology
☐	☒	Animals and other organisms
☐	☒	Human research participants
☒	☐	Clinical data
☒	☐	Dual use research of concern

Methods

n/a	Involved in the study	
☐	☒	ChIP-seq
☒	☐	Flow cytometry
☒	☐	MRI-based neuroimaging

Antibodies

Antibodies used	Primary antibodies against HIF-1α (BD, 610959,1:1000), PKM2 (Cell Signaling Technology, 4053S,1:1000) , PHD3 (Abcam, ab30782,1:1000) , hnRNPK(Santa cruz, Sc53620,1:1000) , hnRNPF(Abcam, Ab50982,1:1000), JMJD5(Abcam, Ab10639,1:1000), Rabbit Hydroxyproline(Abcam, Ab37067,1:1000), Rabbit LaminB1(Cell Signaling Technology, 12255S,1:1000), MnSOD2(BD, 611580,1:1000), Rabbit HIF-2α(CST,59973S,1:1000,Rabbit H3K27me3(CST,9733S,1:1000),Flag(Abcam,ab205606,1:1000),, Digoxin-antibody(Abcam, Ab419,1:100), Mouse β-actin(Abcam, ab6276,1:1000), Rabbit PKM1(proteintech, 15821-1-AP,1:1000) were used.. Standard procedures were used for immunoblotting.<br>Immunohistochemistry (IHC).primary antibodies were used: HIF-1α (BD, 610959,1:50), PKM2 (Cell Signaling Technology, 4053S,1:50) , PHD3 (Abcam, ab30782,1:50),LDHA (Cell Signaling Technology, 3582,1:50), Ki67(Abcam, Ab155809,1:50), GLUT1(Abcam, Ab115730,1:50).<br>Immunofluorescence primary antibody:PKM2 (Cell Signaling Technology, 4053S,1:50) , PHD3 (Abcam, ab30782,1:50), hnRNPK(Santa cruz, Sc53620,1:50) , hnRNPF(Abcam, Ab50982,1:50), JMJD5(Abcam, Ab10639,1:50),Biotin-antibody(Invitrogen, 033700,1:100),secondary antibody Alexa Fluor Goat anti-mouse 594(Invitrogen, A11032,1:500),Alexa Fluor 555(Invitrogen, A28180,1:500).<br>CHIP antibody:HIF-1α (BD, 610959,1:50),p300(abcam,ab54984,1:50),H3K9ac (abcam,ab4441, 1:50),IgG (Abcam,ab172730,1:50)
Validation	All antibodies are commercially available and have been tested for species reactivity and validated by the manufacturers and supplier. The statements of validation of each primary antibody for the species and application are available on the manufacturer's websit

Eukaryotic cell lines

Policy information about cell lines

Cell line source(s)	MDA-MB-231, MDA-MB-468, T47D, BT-474, SKBR3, MCF-7 breast cancer cells, 76N breast epithelial cells and human embryonic kidney cell 293T were obtained from American Type Culture Collection (ATCC) and grown according to standard protocols.
Authentication	All cell lines were authenticated by short tandem repeat (STR)-profiling.
Mycoplasma contamination	All cell lines were tested negative for mycoplasma contamination.
Commonly misidentified lines<br>(See ICLAC register)	No misidentified cell lines were used in this study

Animals and other organisms

Policy information about studies involving animals; ARRIVE guidelines recommended for reporting animal research

Laboratory animals	Balb/c nu/nu mice(3-4 weeks old female) of 4-6 week old were purchased from Beijing Vitonlihua Laboratory Animal Center Co., Ltd. and housed under standard conditions of the room temperature range between 20-26°C, the relative ambient humidity of 50-70%, the semi-natural light cycle of 12:12 or 10:14 hours light:dark.
Wild animals	Wild animals were not involved in this study
Field-collected samples	Field -collected samples were not involved in this study
Ethics oversight	All animal studies were carried out according to the ethical regulations approved by Sun Yat-sen University Animal Care and Use Committee according to the Institutional Animal Care and Use Committee at the Medical School of Sun Yat-Sen University and laboratory animal facility has been accredited by AAALAC (Association for Assessment and Accreditation of Laboratory Animal Care International) and the IACUC (Institutional Animal Care and Use Committee) of Guangdong Laboratory Animal. Monitoring Institute approved all animal protocols used in this study.

Note that full information on the approval of the study protocol must also be provided in the manuscript.

Human research participants

Policy information about studies involving human research participants

Population characteristics	Breast cancer samples were obtained from 493 female breast cancer patients (22-89 years age, mean=49 years) from Sun Yat-Sen Memorial Hospital, Sun Yat-Sen University (Guangzhou, China) between 2008 and 2016.
Recruitment	Patients without any treatment before surgery were recruited. The normal breast tissue and breast cancers were obtained. The data would been affected when normal breast tissue has been mixed with cancer tissue. All samples were collected from patients with informed consent.
Ethics oversight	The institutional review board (IRB) of Sun Yat-Sen Memorial Hospital, Sun Yat-Sen University.

Note that full information on the approval of the study protocol must also be provided in the manuscript.

ChIP-seq

Data deposition

- [x] Confirm that both raw and final processed data have been deposited in a public database such as GEO.
- [x] Confirm that you have deposited or provided access to graph files (e.g. BED files) for the called peaks.

Data access links<br>*May remain private before publication.*	https://bigd.big.ac.cn/gsa/s/Z9K8598l
Files in database submission	ChIPSeq_MDA-MB-231_HIFAL-WT_HIF1a,ChIPSeq_MDA-MB-231_HIFAL-null_HIF1a,ChIPSeq_MDA-MB-231_HIFAL-WT_HIF1a_input,ChIPSeq_MDA-MB-231_HIFAL-null_HIF1a_input,ChIPSeq_MDA-MB-231_antisense_biotin,ChIPSeq_MDA-MB-231_HIFAL_biotin,ChIPSeq_MDA-MB-231_antisense_biotin_Input,ChIPSeq_MDA-MB-231_HIFAL_biotin_Input
Genome browser session<br>(e.g. UCSC)	https://genome-asia.ucsc.edu/s/zifeng9527/HIFproject

Methodology

Replicates	HIFAL WT and HIFAL null （two duplicate), HIFAL-biotin and antisense-biotin(two duplicate)
Sequencing depth	Sequencing depth for each experiment is 30M, they were paired-end.
Antibodies	HIF-1α (BD, 610959),Biotin-antibody(Invitrogen, 033700)
Peak calling parameters	Enriched HIF1a and biotin ChIP peak regions were determined using MACS2 with both ChIP and control (input) samples,Overlapped unique peaks were identified by using BED tools,. Metagenes were created by taking the mean number of reads per 150 bp bin across all regions.
Data quality	aw 150-nucleotide ChIP-seq reads were subjected to quality assessment (97% bases ≥Q30) ,and adapter sequences (~13%) were filtered out ,<br>Metagenes were created by taking the mean number of reads per 150 bp bin across all regions
Software	FASTQC,MACS2, HOMER,BED tools
